# Development and Integration of Genome-Wide Polymorphic Microsatellite Markers onto a Reference Linkage Map for Constructing a High-Density Genetic Map of Chickpea

**DOI:** 10.1371/journal.pone.0125583

**Published:** 2015-05-14

**Authors:** Yash Paul Khajuria, Maneesha S. Saxena, Rashmi Gaur, Debasis Chattopadhyay, Mukesh Jain, Swarup K. Parida, Sabhyata Bhatia

**Affiliations:** National Institute of Plant Genome Research (NIPGR), Aruna Asaf Ali Marg, New Delhi, India; CSIR-National Botanical Research Institute, INDIA

## Abstract

The identification of informative *in silico* polymorphic genomic and genic microsatellite markers by comparing the genome and transcriptome sequences of crop genotypes is a rapid, cost-effective and non-laborious approach for large-scale marker validation and genotyping applications, including construction of high-density genetic maps. We designed 1494 markers, including 1016 genomic and 478 transcript-derived microsatellite markers showing *in-silico* fragment length polymorphism between two parental genotypes (*Cicer arietinum* ICC4958 and *C*. *reticulatum* PI489777) of an inter-specific reference mapping population. High amplification efficiency (87%), experimental validation success rate (81%) and polymorphic potential (55%) of these microsatellite markers suggest their effective use in various applications of chickpea genetics and breeding. Intra-specific polymorphic potential (48%) detected by microsatellite markers in 22 *desi* and *kabuli* chickpea genotypes was lower than inter-specific polymorphic potential (59%). An advanced, high-density, integrated and inter-specific chickpea genetic map (ICC4958 x PI489777) having 1697 map positions spanning 1061.16 cM with an average inter-marker distance of 0.625 cM was constructed by assigning 634 novel informative transcript-derived and genomic microsatellite markers on eight linkage groups (LGs) of our prior documented, 1063 marker-based genetic map. The constructed genome map identified 88, including four major (7–23 cM) longest high-resolution genomic regions on LGs 3, 5 and 8, where the maximum number of novel genomic and genic microsatellite markers were specifically clustered within 1 cM genetic distance. It was for the first time in chickpea that *in silico* FLP analysis at genome-wide level was carried out and such a large number of microsatellite markers were identified, experimentally validated and further used in genetic mapping. To best of our knowledge, in the presently constructed genetic map, we mapped highest number of new sequence-based robust microsatellite markers (634) which is an advancement over the previously documented (~300 markers) inter-specific genetic maps. This advanced high-density map will serve as a foundation for large-scale marker validation and genotyping applications, including identification and targeted mapping of trait-specific genes/QTLs (quantitative trait loci) with sub-optimal use of resources and labour in chickpea.

## Introduction

Microsatellites or simple sequence repeats (SSRs) are well distributed tandem repeats of one- to six- nucleotide long DNA motifs present in coding and non-coding sequence components of crop genomes [1–3]. Variation in the number of microsatellite repeats at a locus among individuals results in different sized amplicons, thereby making them a hypervariable class of PCR-based genetic markers [4]. Microsatellite markers are highly informative and have gained considerable importance over other marker systems for genetic analysis in crop plants because of their abundance, multi-allelic nature, co-dominant inheritance, reproducibility and wide genomic distribution. These markers are also amenable to large-scale genotyping and thus suitable for many applications in structural, functional, and comparative genomics, including construction of high-density genome maps, gene/QTL (quantitative trait loci) mapping and marker-assisted selection [5–16]. In chickpea, the currently available draft genome sequences of both its *desi* (dark coloured small seeds with rough seed coats)and *kabuli* (light coloured large seeds with smoother seed coat) cultivars representing diverse plant architectures and gene pools have generated about 30000 and 81000 microsatellite markers *in silico*, respectively [17,18]. However, experimental validation of such huge numbers of microsatellite markers and selection of smaller sets of informative markers from these larger marker databases that show successful amplification as well as requisite polymorphism in chickpea, is an immensely laborious and tedious task. Moreover, the narrow genetic base in chickpea may impede use of a larger proportion (~70%) of the identified markers in genotyping applications because of their low intra-specific polymorphism among chickpea genotypes [7,9,10,13,19–21]. Further, even the option of selecting the remaining ~30% informative polymorphic microsatellite markers from the huge numbers available in the microsatellite marker database, involves high cost and excessive labor, time and resources. In order to achieve the desired polymorphic potential and for enriching the informative-ness of microsatellite markers, one would also require advanced infrastructural facilities like high resolution genotyping assays (automated fragment analyzer) for precise marker allele sizing and determining their accurate intra-specific allelic variations among chickpea genotypes.

To overcome the limitations involved in individual validation and genotyping of the huge number of available microsatellite markers at a genome-wide scale, alternative strategies using *in silico* analysis may be utilized. This would involve the identification and validation of a smaller set of polymorphic genomic and transcript-derived microsatellite markers showing *in silico* fragment length polymorphism (FLP) between chickpea genotypes based on variation in their number of microsatellite repeats. Similar approaches of identifying *in silico* polymorphic microsatellite markers by comparing the whole genome sequences between two rice sub-species *indica* (*cv*. 93–11) and *japonica* (*cv*. Nipponbare) with 90% experimental validation success rate and their implication in marker-assisted breeding have been well demonstrated in rice [22,23]. In chickpea, similar efforts have also been initiated recently to identify *in silico* polymorphic microsatellites by comparing the available whole genome and transcript sequences among *desi*, *kabuli* and wild chickpea genotypes [17,21,24,25].

From these studies, it is clearly evident that instead of assaying whole genome large-scale microsatellite markers, a smaller set of freely accessible *in silico* polymorphic genomic and transcript-derived microsatellite markers can be targeted primarily for their rapid validation and further utilization in chickpea genome analysis and breeding with sub-optimal use of resources as well as cost, labour and time. Above all, such a strategy would provide flexibility to users for classifying the *in silico* polymorphic microsatellite markers into different groups based on repeat-length variation for accurate resolution and rapid validation of their polymorphic amplified fragments in the available genotyping assays.

Chickpea, being a large genome (~740 Mb), requires numerous informative, chromosome-wise well distributed genomic and transcript-derived microsatellite markers for construction of a high-resolution genetic linkage map in order to identify and map useful candidate genes/QTLs controlling important agronomic traits. In chickpea, saturated genetic linkage maps for an internationally recognized reference mapping population integrating specifically about 300 genomic and genic microsatellite and about a thousand of SNP (single nucleotide polymorphism) markers are available [5,7,9–11,13,15,26]. In the perspective of excellent genetic attributes of microsatellite markers coupled with the basic requirement of only a simple, cost-effective agarose gel-based assay for their efficient genotyping applications, as compared to other random and sequence-based markers, assigning more number of such informative markers to enhance the resolution of the existing chickpea genetic linkage map is always desirable. It would thus expedite and enrich the possibility of identification and targeted mapping of trait-specific genes/QTLs with minimal expense of cost, labor and resources in chickpea.

Keeping the above in view, in the present study, we developed 1494 genomic and transcript-derived microsatellite markers showing *in silico* FLP (based on repeat-unit variation) in the genomic and transcript sequences of chickpea *desi* (*C*. *arietinum cv*. ICC4958) and wild (*C*. *reticulatum cv*. PI489777) genotypes (parents of an internationally recognized reference mapping population). Eight hundred seventy-three microsatellite markers showing ≥ 2 bp FLP between the above genotypes were validated experimentally using gel-based assays and fluorescent-dye labeled automated fragment analyzer in order to assess the potential of these markers for detecting intra-specific polymorphism among *desi* and *kabuli* genotypes. Of these, 636 parental polymorphic microsatellite markers were genotyped and the genotyping information was combined with that of our previously constructed 1063 marker-based genetic map (ICC4958 x PI489777) to develop a high-density improved version of the integrated and inter-specific genetic linkage map of chickpea.

## Materials and Methods

### Development and validation of *in silico* polymorphic microsatellite markers in chickpea

To identify *in silico* polymorphic microsatellites at a genome-wide scale, 200 bp genomic and transcript sequences flanking the either side (5’ and 3’) of microsatellite repeat-motifs were extracted from *C*. *arietinum* cv. *desi* ICC4958, which were further compared with that of flanking sequences of assembled contigs as well as individual reads of wild *C*. *reticulatum* cv. PI489777 using BLASTN. The matching 3´ and/or 5´ flanking sequences (*E*-value cut-off: <1e-500 with more than 90% sequence homology) of ICC4958 showing expansion/contraction of similar microsatellite repeats in the assembled contigs (>80% of individual reads representing similar microsatellite allele types) of PI489777 and were considered as *in silico* polymorphic microsatellites. The remaining ~20% of individual reads of PI489777 with different alleles of same microsatellite repeat-motifs as compared to ICC4958 were not included in our analysis. All these aforementioned individual steps to be followed for detection of *in silico* polymorphic microsatellites were integrated in our developed custom-based Perl Scripts (provided in S1 Table). Nevertheless, one can make use of these defined methods without Perl Scripts as well for identification of polymorphic microsatellites at a genome-wide scale in diverse crop plants, including chickpea. Based on these analyses, 2075 genomic microsatellites showing *in silico* FLP (2 to >200 bp) between whole genome sequences of ICC4958 and PI489777 based on variations in the number of microsatellite repeats were obtained [17]. The forward and reverse primers based on the high-quality genomic sequences of ICC4958 flanking these polymorphic microsatellite repeat-motifs were designed using the Primer3 interface tool of MISA (Microsatellite, http://pgrc.ipk-gatersleben.de/misa/) and BatchPrimer3 (http://probes.pw.usda.gov/cgi-bin/batchprimer3/batchprimer3.cgi). Further, our previously reported information on 561 transcript-derived microsatellites showing *in silico* FLP between transcript sequences of ICC4958 and PI489777 [25] was also used. The forward and reverse primers from the high-quality transcript sequences of ICC4958 flanking these 561 polymorphic microsatellite repeat-motifs were designed following the procedures as described above. Primer-pairs could be designed for 1494, including 1016 genomic and 478 transcript-derived perfect and compound microsatellites showing well-defined repeat-unit (2 to 200 bp) expansion/contraction between ICC4958 and PI489777. These 1494 identified microsatellite markers were characterized into perfect (di- to hexa-nucleotides) and compound microsatellite types based on characteristics of their repeat-motifs. The compound microsatellites were classified into non-interrupting [for example, CaGMS1164: (AG)_9_(GT)_6_] and interrupting [maximum 100 nucleotides interrupting two microsatellites, e.g. CaGMS1125: (AT)_8_TGCTTTTTATTATTTATAA(AT)_7_] types. According to length of the microsatellite repeat-motifs, the microsatellites were categorized into hypervariable class I (≥ 20 bp) and class II (12–20 bp). The remaining 1142, including 1059 genomic and 83 transcript-derived microsatellites could not be analyzed due to their inefficient primer designing potential (409 microsatellites) and/or longer (>200 bp) repeat-unit expansion/contraction between ICC4958 and PI489777 (733 microsatellites). A set of 1180, including 702 genomic and 478 transcript-derived perfect microsatellite markers showing *in silico* FLP between ICC4958 and PI489777 based on 2 to 150 bp variation in the repeat- units were selected for their validation through gel based assay and fluorescent dye-labeled automated fragment analyzer. The rest 314 microsatellite markers with greater degree of expansion/contraction of their repeat-units (150 to 200 bp) between ICC4958 and PI489777 were excluded from experimental validation. To evaluate the amplification and experimental validation success rate of designed *in silico* polymorphic microsatellite markers, the primer-pairs for the selected 1180 (702 genomic and 478 transcript-derived) microsatellite markers were synthesized (BIONEER Corporation, Korea). PCR amplification of genomic DNA of ICC4958 and PI489777 using touchdown thermal cycling profiling and standard PCR constituents was carried out in an Applied Biosystems (ABI, Illinois, USA) thermal cycler. The PCR constituents, including 10 μl volume containing 1 μl of 10x Paq buffer A, 0.5 μl of 10 mM dNTPs, 0.1 μl of 5 unit of Paq5000 DNA polymerase (Stratagene, Agilent Technologies, USA), 1 μl (25–30 ng) of template genomic DNA and 1 μl (5 μM) each of forward and reverse microsatellite primers (normal and/or fluorescent dye labeled) were used for amplification, following the methods as described by Jhanwar et al. [25]. The PCR products amplified by each microsatellite marker in the two chickpea genotypes (ICC4958 and PI489777) were resolved on 3.5% metaphor agarose gel and/or fluorescent dye-labeled automated fragment analyzer. For automated fragment analysis, the amplified fluorescent dye-labeled PCR products were resolved in automated 96 capillary ABI3730xl DNA Analyzer and the electrophoregram containing trace files were analyzed using GeneMapper V4.0. The GC-content (%) of genomic and transcript sequences (from which the primers were designed and showed successful amplification) were estimated according to Jain et al. [17] and Jhanwar et al. [25]. The actual allele size (bp) and FLP (bp) detected by the microsatellite markers between ICC4958 and PI489777 using both gel-based assay and automated fragment analyzer was determined and correlated.

To confirm that the designed microsatellite markers amplified the expected repeat-motifs and to derive the correspondence of FLP with repeat length expansion/contraction between two chickpea genotypes, the amplified PCR products of size variant amplicons generated from selected polymorphic markers were purified and cloned in pGEM-T Easy Vector (Promega, USA). Twelve clones of each marker amplicon were further sequenced in both forward and reverse directions twice on a capillary-based Automated DNA Sequencer (Applied Biosystems, ABI 3730xl DNA Analyzer) using BigDye Terminator v3.1 sequencing kit and M13 forward and reverse primers. The high-quality consensus sequences obtained for each marker were aligned using CLUSTALW multiple alignment tool and compared between two genotypes.

### Assessment of polymorphic potential of microsatellite markers

To evaluate the polymorphic potential of developed microsatellite markers, 100 each of validated genomic and transcript-derived microsatellite markers showing polymorphism between ICC4958 and PI489777 in the gel based assay were used to further amplify the genomic DNA isolated from 22 *desi* and *kabuli* chickpea genotypes using the aforesaid methods. The amplified PCR products were resolved in 3.5% metaphor agarose gel and FLP (bp) detected by these markers among genotypes was determined. The genotyping data of all microsatellite markers were used to estimate the average polymorphic alleles per marker, percent polymorphism and polymorphism information content (PIC) among *desi* and *kabuli* chickpea genotypes.

### Genotyping of informative microsatellite markers and construction of an integrated inter-specific chickpea genetic linkage map

The validated 636, including 175 genomic and 461 transcript-derived microsatellite markers showing FLP (≥6 bp) between ICC4958 and PI489777 either by gel-based assay or automated fragment analyzer were PCR amplified and genotyped using the genomic DNA of 94 RILs (recombinant inbred lines) derived from an internationally well recognized inter-specific mapping population (ICC4958 x PI489777) along with their parents. The genotyping information of all the 636 microsatellite markers was utilized for linkage analysis and construction of genetic map using JoinMap V4.1 (http://www.kyazma.nl/index.php/mc) at higher logarithm of odds (LOD) threshold with Kosambi function. We recently constructed a 1063 marker (genic EST-derived and genomic microsatellite and SNP markers and intron targeted primers)-based saturated genetic linkage map of chickpea [26] using the same inter-specific mapping population (ICC4958 x PI489777). To further develop a high-density, more advanced and integrated genetic map of chickpea, the genotyping information of 1063 markers allocated on the already available genetic linkage map, was integrated with that obtained from our newly genotyped 636 microsatellite markers using the procedures as described by Gaur et al. [26].

## Results and Discussion

### Large-scale validation of *in silico* polymorphic genomic and transcript-derived microsatellite markers in chickpea

**Fig 1:**
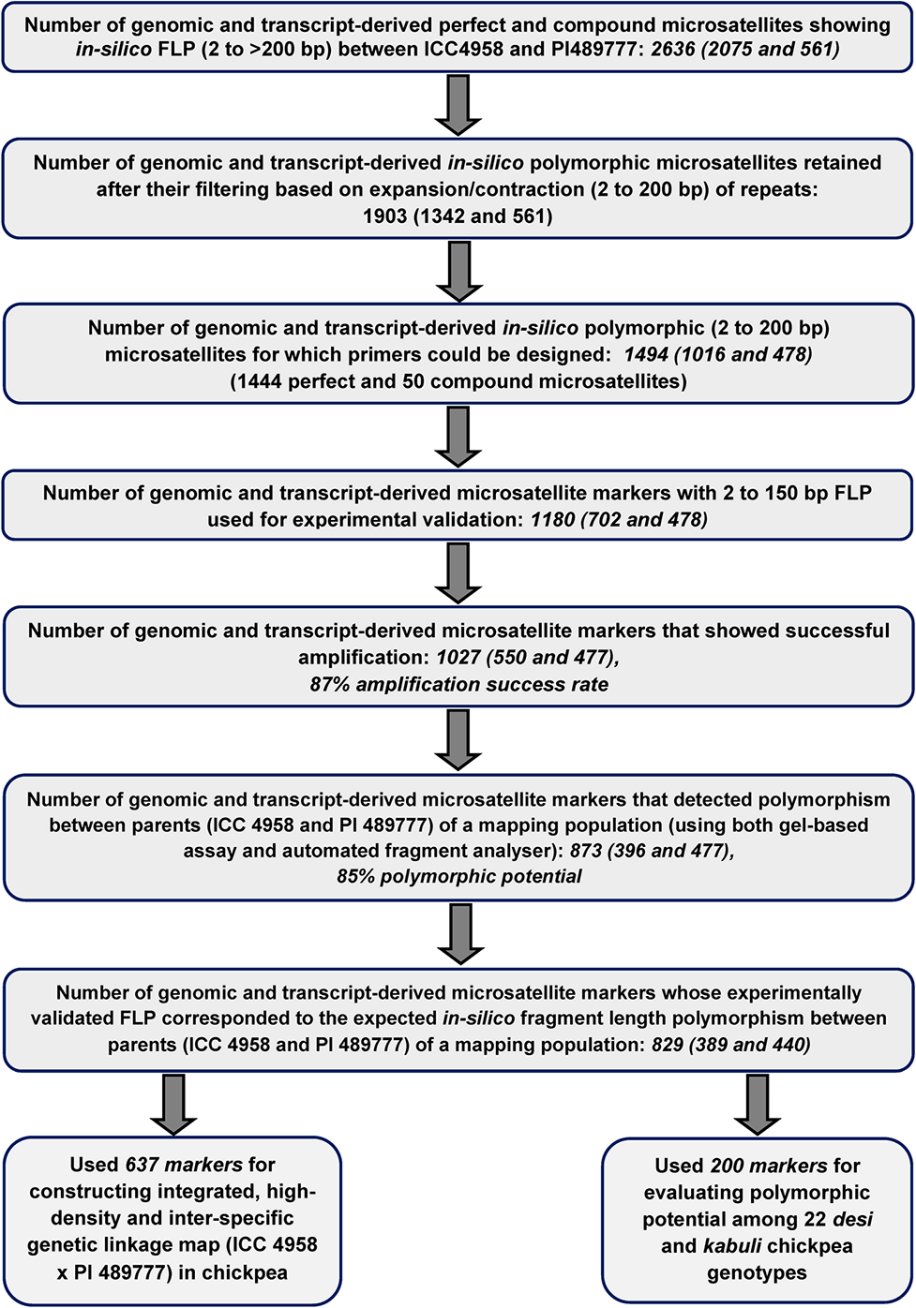
Summary of genomic and transcript-derived in *silico* polymorphic microsatellite markers used in the present study.

**Fig 2:**
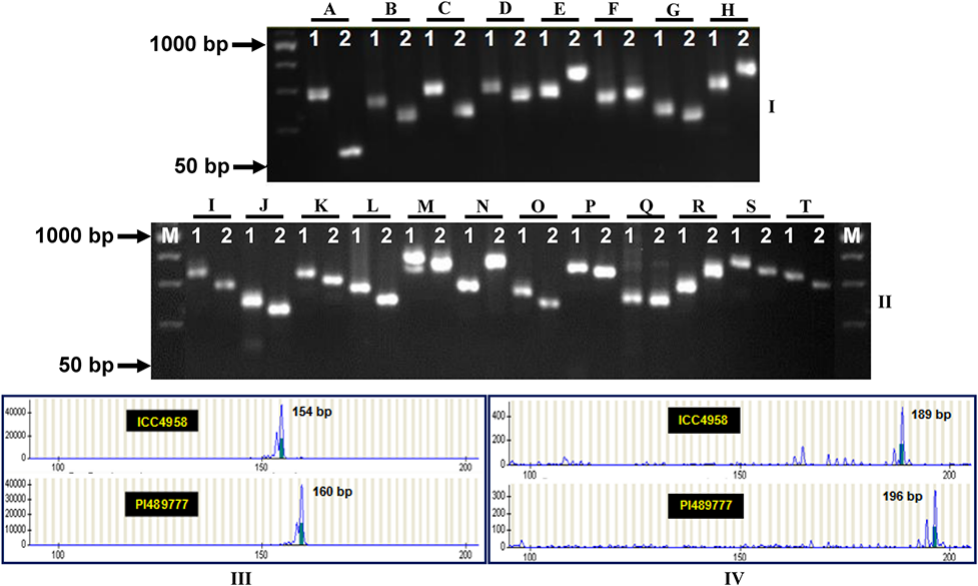
Validation of a representative set of novel transcript-derived (I) and genomic (II) microsatellite markers showing *in silico* FLP between ICC4958 (1) and PI489777 (2) using the gel-based assay (I and II) and fluorescent dye labeled automated fragment analyzer (III and IV). The fragment sizes (bp) of the amplified polymorphic alleles are indicated. The identities of markers (A: CaTMS616, B: CaTMS654, C: CaTMS715, D: CaTMS716, E: CaTMS561, F: CaTMS577, G: CaTMS783, H: CaTMS651, I: CaGMS1, J: CaGMS3, K: CaGMS13, L: CaGMS16, M: CaGMS18, N: CaGMS19, O: CaGMS24, P: CaGMS23, Q: CaGMS20, R: CaGMS41, S: CaGMS43 and T: CaGMS45) with their detailed information are provided in the S2 Table. The primers CaTMS606 and CaGMS40 were used for automated fragment analysis (III and IV). M: 50 bp DNA ladder size standard.

One thousand four hundred ninety-four, including 1016 genomic and 478 transcript-derived microsatellite markers showing *in silico* FLP (2 to 200 bp) between ICC4958 and PI489777 based on variation in the repeats were developed in chickpea (Fig 1, S2 Table) by comparing their genomic and transcriptomic sequences, respectively. Of these, 1180 markers showing minimum 2 to 150 bp FLP between ICC4958 and PI489777 (Fig 1, S2 Table) were selected for experimental validation using gel-based assay and fluorescent dye-labeled automated fragment analysis. To evaluate the potential of markers to amplify the target microsatellite repeat sequences and validate polymorphism experimentally as predicted, all 1180 microsatellite markers were used to PCR amplify the genomic DNA of the two chickpea genotypes (ICC4958 and PI489777). Of these, 1027 genomic and transcript-derived microsatellite markers (Fig 2) produced single reproducible PCR amplicons in 3.5% metaphor agarose gel (with a range of around 55–60°C annealing temperatures) with an average amplification success rate of 87%. Higher amplification efficiency of transcript-derived microsatellite markers (99.8%) in contrast to genomic markers (78.3%) was possibly due to the greater primer-binding potential of markers designed from the more conserved transcript sequences with balanced GC content (50–55%). Higher null-allelic amplification in case of 21.7% genomic microsatellite markers could be due to insertion/deletion in the corresponding flanking non-conserved genomic (intronic) sequences with low GC content (30–40%) and thus resulted in alteration of the primer-binding sites of markers. The average amplification efficiency (99.8%) of transcript-derived microsatellite markers estimated in this study was significantly higher than that observed in an earlier study (88%) of Hiremath et al. [21], but comparable to that obtained (98%) by Jhanwar et al. [25] and Agarwal et al. [24]. Overall, it indicated the utility of available whole genome and transcriptome sequence resources for developing large-scale more informative genomic and transcript-derived microsatellite markers in chickpea.

**Fig 3:**
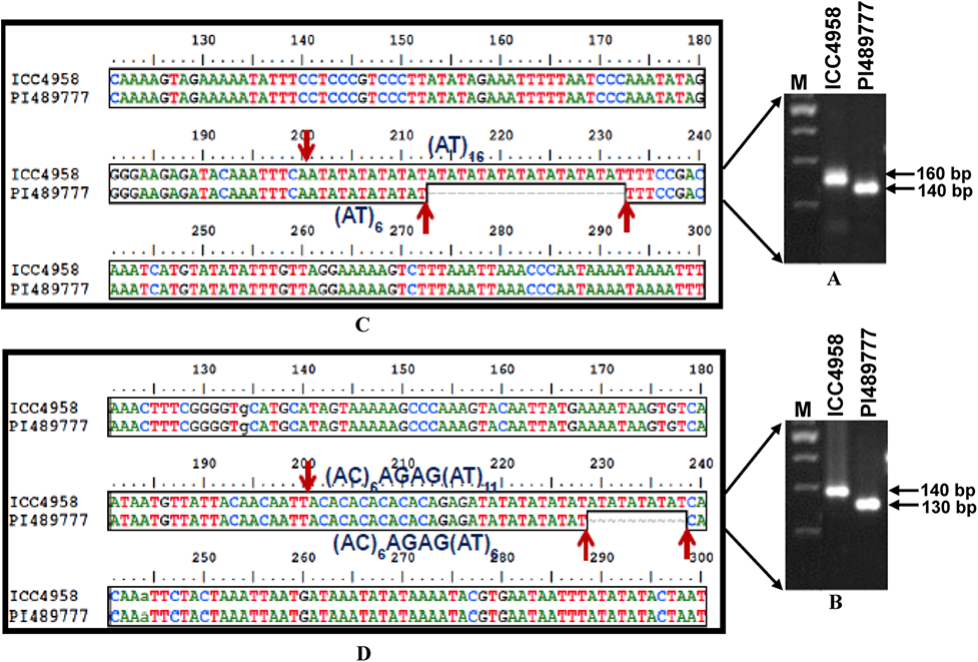
The sequencing of cloned amplicons from different perfect transcript-derived (I) (CaTMS1055) and compound genomic (II) (CaGMS1156) microsatellite markers showing FLP between ICC4958 and PI489777 and their multiple sequence alignment (C and D) validated the presence of expected microsatellite repeat-motifs which corresponded well with our *in silico* prediction of expansion and contraction of a number of microsatellite repeat-units. The fragment sizes (bp) of the amplified polymorphic alleles are indicated. M: 50 bp DNA ladder size standard. The identities of two markers with their detailed information are provided in the S2 Table.

Eight hundred seventy-three (85%) of 1027 amplified *in silico* polymorphic markers that got experimentally validated (using both gel-based assay and automated fragment analyzer) were found to be polymorphic between the two parental genotypes (ICC4958 and PI489777) of mapping population (Figs 1 and 2, S2 Table). Microsatellite markers showing ≥10 bp and 2–9 bp *in silico* FLP between ICC4958 and PI489777 were resolved on metaphor agarose gels and automated fragment analyzer, respectively. The rest 154 (15%) of 1027 amplified microsatellite markers revealing 2 to 4 bp FLP between ICC4958 and PI489777 could not be resolved even in high-resolution automated fragment analyzer. Very interestingly, in case of 829 (95% of 873 markers) markers, the actual FLP observed between ICC4958 and PI489777 by experimental validation corresponded with their predicted *in silico* polymorphism as expected based on variation in the number of microsatellite repeat-units (Fig 1, S2 Table). The correspondence of FLP of markers between ICC4958 and PI489777 with expansion and contraction of microsatellite repeats was clearly evident from the cloning and sequencing of size variant amplicons of a selected set of microsatellite markers from the chickpea genotypes (Fig 3). The experimental validation success rate estimated particularly for *in silico* polymorphic transcript-derived microsatellite markers (92.2%) is comparable to that reported recently by Jhanwar et al. [25] and Agarwal et al. [24]. Therefore, the development of polymorphic microsatellite markers *in silico* by comparing their repeat length variation in the genomic and transcript sequences between ICC4958 and PI489777 proved to be a non-laborious, cost-effective and time saving approach for their large-scale validation and genotyping applications in chickpea. This fast strategy of identifying informative microsatellite markers (85%) would be most relevant in case of chickpea where only about 20–30% markers are expected to be polymorphic while assaying genome-wide intra- and inter-specific random microsatellite markers. Necessarily, the larger chickpea genome (~740 Mb) requires huge number of informative microsatellite markers to construct a high-density genetic linkage map and identify markers tightly linked to the genes/QTLs controlling important agronomic traits for its efficient marker-assisted genetic improvement. In this perspective, a large number of experimentally validated *in silico* polymorphic microsatellite markers developed in this study (Fig 1) from the whole genome, including transcript sequences of chickpea, would have wider utility as informative markers for many applications in chickpea genetics, genomics and breeding.

### Polymorphic potential of transcript-derived and genomic microsatellite markers

**Fig 4:**
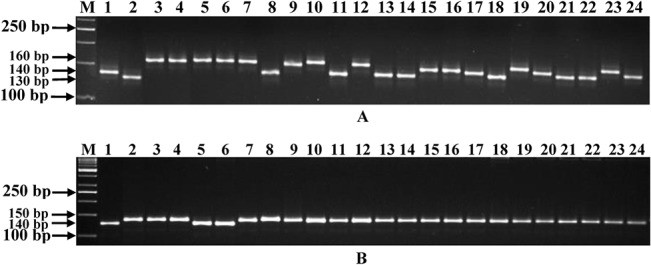
Allelic variations detected among 22 *desi* and *kabuli* genotypes along with controls (ICC4958 and PI489777) using a representative set of novel genomic (A) (CaGMS24) and transcript-derived (CaTMS561) (B) microsatellite markers in gel-based assay. A maximum number of four polymorphic alleles were amplified by the genomic microsatellite markers, while transcript-derived microsatellite markers produced two alleles among genotypes. The fragment sizes (bp) of the amplified polymorphic alleles are indicated. The genotypes used are, 1: ICC4958, 2: PI489777, 3: Pusa362, 4: Himchana1, 5:ICCC4, 6: Dilaji, 7: JG11, 8: Bharathi, 9: BGD112, 10: Pusa256, 11: JG62, 12: Kranthi, 13: JAKI9218, 14:Swetha, 15: JGK3, 16: PKV Kabuli2, 17: Vihar, 18: LBeG7, 19: BG5023, 20: Pusa1088, 21: BGD1105, 22: PG515, 23: Pusa1108 and 24: JGK2. M: 50 bp DNA ladder size standard. The identities of two markers with their detailed information are provided in the S2 Table.

A set of 100 each of experimentally validated, polymorphic genomic and transcript-derived microsatellite markers were used (S2 Table) to study the polymorphic potential among 22 *desi* and *kabuli* genotypes. One hundred ten (55%) of 200 markers were found to be polymorphic (with average PIC of 0.61) between at least two combinations of genotypes (Fig 4) and included 27 of 38 (71%, with average PIC of 0.69) hypervariable class I and 38 of 72 (52.8%, 0.43) class II markers. Higher polymorphic potential of class I microsatellite markers could be due to higher length dependent replication slippage of their longer (≥ 20 bp) hypervariable repeat-motifs [2,27–29]. The potential of polymorphism detected by genomic microsatellite markers (62 of 100 markers, 62% polymorphism, mean PIC 0.62 and 1 to 4 alleles) in *desi* and *kabuli* chickpea genotypes was higher than that obtained with the transcript-derived microsatellite markers (48 of 100 markers, 48%, 0.54 and 1 to 3). Lower polymorphic potential of transcript-derived microsatellite markers as compared to genomic markers is possibly due to their derivation from the conserved expressed component of the genome, which are under strong selection pressure [2,3,28,29]. Sixty seven (61%) of the 110 markers showed polymorphism among 11 *desi* chickpea genotypes (varied from 1 to 4 alleles and mean PIC of 0.60), whereas 43 (39%) markers detected polymorphism in the remaining 11 *kabuli* genotypes (1 to 3 and 0.42). The extent of polymorphism detected by markers between *desi* and *kabuli* genotypes (59%) was higher than that obtained within *desi* and *kabuli* genotypes (48%). Two hundred genomic and transcript-derived microsatellite markers overall produced a total of 364 alleles in 22 genotypes. The number of alleles detected by these markers varied from 1 to 4 alleles with an average of 3 alleles per marker. The intra-specific polymorphism detected by genomic and transcript-derived microsatellite markers among 22 chickpea genotypes (55%) based on gel based assay was comparable to that estimated earlier using *in silico* polymorphic genic microsatellite markers (50–60%) [21,24]. Overall, the *in silico* polymorphic genomic and transcript-derived microsatellite markers designed and experimentally validated in this study revealed higher intra-specific polymorphic potential as compared to that estimated with random genome-wide microsatellite markers (~35%) [7,10,19–21] among *desi* and *kabuli* chickpea genotypes. It thus suggests the utility of developing such kinds of highly informative *in silico* polymorphic microsatellite markers and their efficient utilization in large-scale and high-throughput genotyping applications in chickpea. Specifically, the *in silico* polymorphic transcript-derived microsatellite markers with relatively high intra-specific polymorphic potential developed in this study could be useful as functional markers for rapidly establishing marker-trait linkages and identifying genes/QTLs for many important agronomic traits in chickpea.

### Construction of a high-density, integrated and inter-specific chickpea genetic linkage map

**Fig 5:**
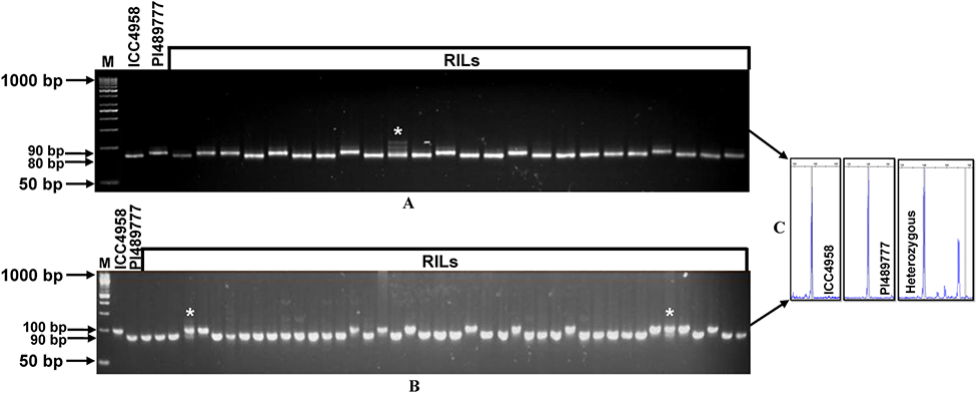
Segregation pattern of transcript-derived (A) (CaTMS651) and genomic (B) (CaGMS16) microsatellite markers in a representative set of mapping individuals of a RIL population derived from the inter-specific cross between ICC4958 and PI489777 along with parental genotypes. The amplified microsatellite marker alleles are resolved using agarose gel-based assay and fluorescent dye-labeled automated fragment analyzer. The fragment sizes (bp) of the amplified parental polymorphic alleles are indicated. M: 50 bp DNA ladder size standard. *indicates the heterozygous alleles amplified by microsatellite markers which are further confirmed through automated fragment analysis (C). The identities of two markers with their detailed information are provided in the S2 Table.

**Fig 6:**
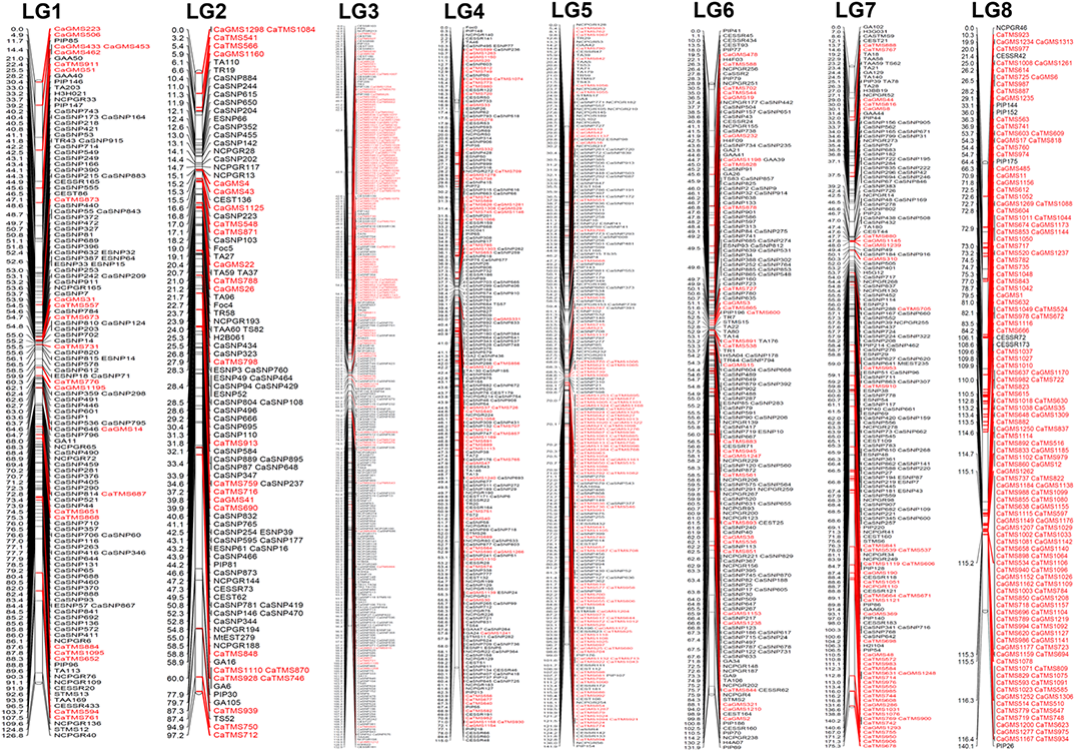
An advanced inter-specific, high-resolution and integrated genetic map (ICC4958 x PI489777) of chickpea constructed by assigning 634 novel genomic and transcript-derived microsatellite markers on eight LGs of a previously reported similar 1063 marker-based genetic map (Gaur et al. [26]). The genetic distance (cM) and identity of the marker loci integrated are indicated on the left and right side of eight LGs, respectively. The earlier reported markers are considered as anchor markers to define eight LGs. The LGs are specified with *Arabic* numerals on the upper-side corresponding with the genetic map as reported by Gaur et al. [26]. The newly integrated genomic and transcript-derived microsatellite markers in this study are highlighted with red colour. Markers designated as CaGMS represent *Cicer arietinum* genomic microsatellite markers, whereas CaTMS represent genic *C*. *arietinum* transcript-derived microsatellite markers.

**Fig 7:**
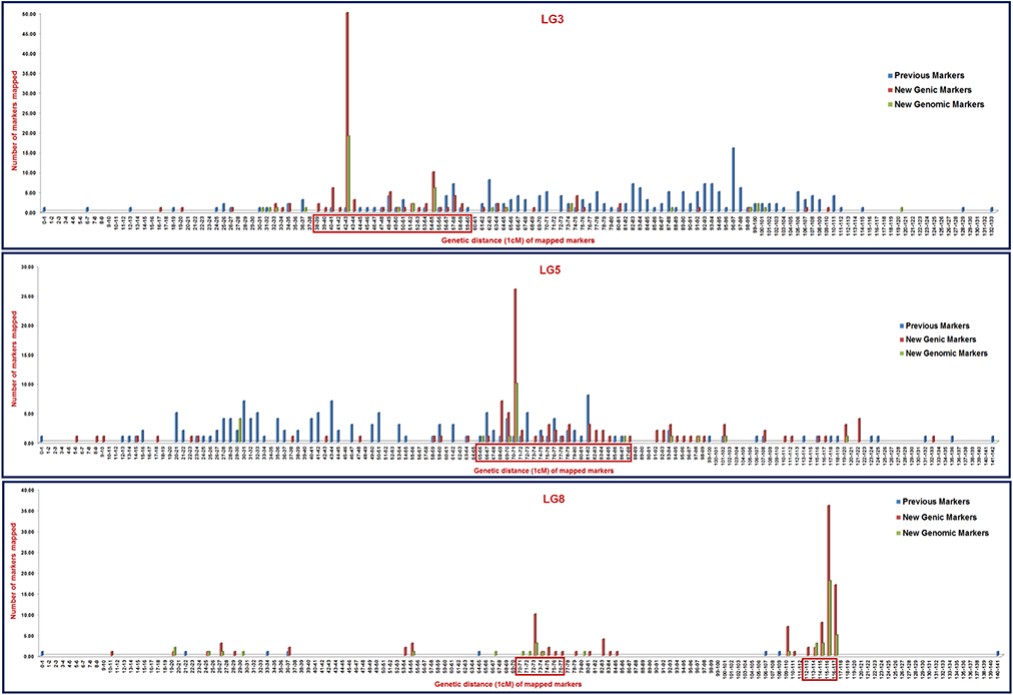
Frequency distribution of novel genomic and transcript-derived microsatellite markers mapped within 1 cM genetic distance on eight LGs of an integrated and inter-specific chickpea genetic linkage map (ICC4958 x PI489777) identified four major high-resolution genomic regions on LG3, LG5 and LG8 with higher marker map density (1–50 markers within 1 cM genetic distance). These four longest high-resolution genomic regions showed a persistent distribution of mapped markers up to 5–23 cM genetic distance. The hot spot regions (cM) on LG3, LG5 and LG8 showing higher map density of microsatellite markers are marked with boxes.

For construction of a saturated and inter-specific genetic linkage map, 636 novel (non-overlapping with previous reported [26] microsatellite markers) microsatellite markers including 175 genomic and 461 transcript-derived microsatellite markers showing parental polymorphism (≥6 bp repeat-unit variation) between ICC4958 and PI489777 (S2 Table) were genotyped among 94 individuals of an internationally recognized reference RIL mapping population (ICC4958 x PI489777) (Fig 5). The genotyping information obtained from these 636 microsatellite markers was combined with that of our previously reported 1063 (238 genomic and 52 EST-derived microsatellites, 27 gene/EST-based, 51 intron targeted primers and 696 SNPs) marker-based genetic map [26] to develop an advanced, integrated and highly saturated inter-specific genetic map of chickpea. Linkage analysis of the 1699 marker (636 + 1063) genotyping data mapped 1697 (634 including 460 transcript-derived and 174 genomic microsatellite markers, S2 Table and 1063 earlier reported markers) marker loci onto eight linkage groups (LGs) to generate an integrated genetic map of chickpea (Fig 6). The remaining two of 636 microsatellite markers exhibiting segregation distortion could not be mapped on an inter-specific genetic linkage map. The LGs were designated with *Arabic* numerals (LG1 to LG8, haploid chromosome numbers) according to their common marker positions and groupings shared between corresponding LGs as documented by earlier studies [5,7,10,11,13,15,21,26]. The integrated genetic map constructed for eight LGs spanned a total map length of 1061.156 cM with an average inter-marker distance of 0.625 cM (Fig 6, Table 1). Assuming 740 Mb genome size of chickpea, the integrated 1697 marker-based genetic linkage map constructed in this study had average marker density of one marker per 436 kb. Maximum number of 376 markers were mapped on LG3 followed by LG5 (276) and LG4 (251) and minimum on LG2 (105) (Table 1). More detailed analysis revealed mapping of highest number of 164 genomic and transcript-derived microsatellite markers (validated in the present study) on LG3, followed by LG8 (154) and LG5 (124) and least on the LG1 (23) (Table 1). Based on genetic distance, the LG7 revealed longest map length spanning 175.335 cM, while the LG2 had shortest map length of 97.218 cM. The average inter-marker distance varied from 0.350 cM for LG3 to 1.080 cM in LG2. The LG3 was the most saturated linkage group, while LG2 was the least saturated (Table 1). Overall, one LG of the genetic map on an average contained 212 markers with total mean map length of 132.64 cM. Remarkably, we identified 88 major high-resolution target genomic regions on eight LGs (on an average 11 high-resolution regionsper LG) of chickpea genetic map where more than two markers were mapped and clustered within 1 cM genetic distance. It comprised of 19 long high-resolution mapped genomic regions on eight LGs showing persistent distribution of markers (ranging from 18–169 markers) up to 5 cM genetic distance. Longest high-resolution mapped genomic regions were identified on LG3 (covered 31.7 cM with 130 markers), followed by LG4 (28.8 cM, 169 markers) and shortest on LG7 (5.5 cM, 18 markers). More interestingly, we identified one longest high-resolution mapped genomic region (38–60 cM) on LG3, one (65–88 cM) on LG5 and two (70–77 cM and 112–117 cM) on LG8 covered specifically with maximum number of genomic and transcript-derived microsatellite markers validated in this study (Fig 7). The useful characteristics of our newly constructed high-density genetic linkage map in contrast to that of previously reported inter-specific genetic maps are highlighted in the Table 2.

**Table 1:** Markers mapped on the eight LGs of an integrated and inter-specific genetic map of chickpea.

Linkage groups (LGs)	Novel genomic and transcript-derived microsatellite markers mapped + previously mapped markers^a^	Map length covered (cM)	Average inter-marker distance (cM)
LG1	23 + 117 = 140	126.774	0.899
LG2	27 + 78 = 105	97.218	1.080
LG3	164 + 212 = 376	132.884	0.350
LG4	65 + 186 = 251	114.998	0.450
LG5	124 + 152 = 276	141.884	0.514
LG6	30 + 159 = 189	131.940	0.690
LG7	47 + 151 = 198	175.335	0.885
LG8	154 + 8 = 162	140.123	0.720
**Total**	**634 + 1063 = 1697**	**1061.156**	**0.625**

^a^Markers mapped on the eight LGs of a previously reported inter-specific genetic map (ICC4958 x PI489777) constructed by Gaur et al. [26].

**Table 2:** Comparative overview on key features between presently constructed and previously generated inter-specific genetic linkage map (ICC4958 x PI489777).

Characteristics	Currently constructed genetic linkage map	Previously generated genetic linkage map26
Number of markers genetically mapped on eight LGs	1697 (*higher number of markers mapped*)	1063
Number of (genomic and transcript-derived) microsatellite markers mapped on eight LGs	634 (174 and 460) (*higher number of microsatellite markers mapped*)	290 (238 and 52)
Total map length (cM) covered by eight LGs	1061.156 (*shorter map-length*)	1808.7
Average marker-density (inter-marker distance in cM)	0.625 (*high-density genetic map*)	1.70

The more advanced and integrated transcript-derived and genomic microsatellite and SNP marker-based chickpea inter-specific genetic map comprising of eight LGs constructed in our study using a single reference mapping population (ICC4958 x PI489777) support the previous documentation using large-scale random and sequence-based markers [5,7,10,11,13,15,26]. The average inter-marker distance (0.625 cM) and total map length (1061.156 cM) estimated for the presently generated chickpea genetic map was lower and thus highly saturated as compared to that documented previously by our similar inter-specific (ICC4958 x PI489777) genetic linkage map (1.7 cM, 1808.7 cM) constructed using 1063 markers [26]. Similar studies have also been carried out by Thudi et al. [11] and Hiremath et al. [15] in which the genetic maps spanning 845.56 and 788.6 cM with average inter-marker distance of 0.65 and 0.59 cM were constructed primarily utilizing 1291 and 1328 microsatellite, SNP and DArT (diversity array technology) markers, respectively. However, all of these studies till date have used at most 300 genomic and genic microsatellite markers and additionally other kinds of random and sequence-based markers including RAPDs (random amplified polymorphic DNA), ISSRs (inter simple sequence repeats), CAPS (cleaved amplified polymorphisms), intron-spanning markers, SNPs and DArT markers to construct genetic linkage maps of chickpea. In contrast, in the presently constructed high-density genetic linkage map, we were able to assign 634 novel, co-dominant, sequence-based genomic and transcript-derived microsatellite markers, which is much higher than the number of microsatellite markers (~300 markers) mapped till date in chickpea. Remarkably, the genetic map constructed in our study had a very high map density (0.625 cM) and thus was an advanced, highly saturated chickpea genetic map in comparison to all other intra- and inter-specific genetic linkage maps reported so far in chickpea (Table 2). Considering the desirable genetic attributes of microsatellite markers (multi-allelic, reproducibility and co-dominance) and their relatively simple, non-laborious and cost-effective validation and genotyping applications using the gel-based assay in comparison to other random and sequence-based markers, the large-scale transcript-derived and genomic microsatellite marker-based genetic linkage map constructed in the present study, will be of great practical significance in laboratories where infrastructural facilities (high-throughput SNP and DArT marker genotyping platforms) are lacking. Therefore, the microsatellite marker-based integrated, high-density and inter-specific genetic linkage map would be useful for mapping the whole genome and rapid targeted mapping of genes/QTLs controlling useful agronomic traits in chickpea as well as comparative mapping across legumes.

## Supporting Information

S1 Table Custom-made PERL script for identification of polymorphic SSRs. (PDF)

S2 Table Details of 1494, including 873 experimentally validated novel genomic and genic microsatellite markers showing *in silico* fragment length polymorphism (based on repeat-unit variation) between ICC4958 and PI489777. (PDF)
