# Supplementary material for: Development and Integration of Genome-Wide Polymorphic Microsatellite Markers onto a Reference Linkage Map for Constructing a High-Density Genetic Map of Chickpea
Source: PLoS One. 2015 May 14;10(5):e0125583. doi: 10.1371/journal.pone.0125583 (PMC4431833; doi:10.1371/journal.pone.0125583)
Supplement: S1 Table — (PDF) [file pone.0125583.s001.pdf]

Table S1: Custom-made PERL script for identification of polymorphic SSR

SCRIPT 1 : EXTRACTION OF FLANKING SEQUENCE (5 PRIME)

```
#! usr/bin/perl -w

#use strict;

#use Term::ANSIColor;

open (FILE1, "$ARGV[0]") or die $!;
#GENOME
open (FILE2, "$ARGV[1]") or die $!;
#ID_

open (FILEW2, ">upstream_seq.txt") or die $!;

while ($line1 = <FILE1>)

{ chomp($line1);
if($line1 =~ />(.*)/)
{

$hash{"$id"} = $fasta;

$id = "$1";

print "$id\n";

#print "$id\n";

$fasta="";

}

  else {

$fasta .= $line1;}

}

$hash{"$id"} = $fasta;

@file3=<FILE2>;

$i=0;

#$feature=0;


foreach $line(@file3){
```

```
@char=split('\t',$line);

$gene_id=$char[0];

$location=$char[1];

$start=$char[5];

$end=$char[6];
if(exists ($hash{$location})){

$data=$hash{$location};

$len=$end-$start;

                $p_start=($char[5]-1501);

                $p_end=($char[5]-1);

                $len=$p_end-$p_start;
$seq=substr($data,$p_start-1,$len+1);

                  }

print FILEW2 ">$gene_id\n$seq\n";

$i++;

                  }

}
```

SCRIPT 2 : EXTRACTION OF FLANKING SEQUENCES (3 PRIME)

```
#! usr/bin/perl -w

open (FILE1, "$ARGV[0]") or die $!;#genome

sequence open (FILE2, "$ARGV[1]") or die

$!;#id_list

     open (FILEW2, ">downstream_seq.txt") or die $!;


    while ($line1 =
       <FILE1>)

    {

    chomp($line1);
```

```
    if($line1 =~
    />(.*)/)
        {
        $hash{"$id"} = $fasta;
        $id = "$1";
        print "$id\n";
        $fasta="";
        }
     els
     e {
          $fasta .= $line1;}
}
$hash{"$id"} = $fasta;
  @file3=<FILE2>;
  $i=0;
  #$feature=0;

    foreach $line(@file3){
            @char=split('\t',$line);
            $location=$char[1];
            $start=$char[5];
            $end=$char[6];
            $gene_id=$char[0];
             if(exists ($hash{$location})){
                  $data=$hash{$location};
                  $len=$end-$start;
```

```
$u_start=($char[6]+1);

$u_end=($char[6]+101);
        len=$u_end-$u_start;

        $seq=substr($data,$u_start-1,$len+1);

    }

      print FILEW2 ">$gene_id\n$seq\n";

      $i++;

            }
```

BLAST COMMNADS: TO COMPARE THE SSEQUENCES

```
makeblastdb -in upstream_seq_variety1.txt -out chickpea_database_up -dbtype nucl

blastn -db chickpea_database_up -query upstream_variety_2.txt  -out
upstream_variety1_variety2.txt - outfmt 7

makeblastdb -in downstream_seq_variety1.txt -out chickpea_database_down -dbtype nucl

blastn -db chickpea_database_down -query upstream_variety_2.txt  -
out downstream_variety1_variety2.txt -outfmt 7
```

SCRIPT 3: COMPARE THE FLANKING SEQUENCES (80% COVERAGE, 100% MATCH)

```
#!/usr/bin/perl

 open(fp,'upstream_variety1_variety2.t

 xt');

 open(fout,">matching_flanking_upstream_variety1_variety_2.txt");

@a=<fp>;

chomp @a;
            for ($j=0;$j<scalar(@a);$j++)

                 {

                    @d=split(/\s+/,$a[$j]);
```

```
            $d[0]=~s/\s+//g;

            @e=split(/\:/,$d[0]);

            $coverage=($e[0]*0.8)

        if ($d[0]>=$coverage) and ($d[1]==100)
                {
               print fout "$a[$j]\n";

                }
          }
```

SCRIPT: MATCHING THE SAME FLANKING WITH DIFFERENT REPEAT NUMBER OF SSR

```
#!/usr/bin/perl

  open(fp,'matching_flanking_upstream_variety1_variety_2.tx

  t'); open(fs,'id_variety_1.txt');

  open(fv,'id_variety_2.txt');

  open(fout,">polyssr_list.txt");

@a=<fp>;

@b=<fs>;

@c=<fv>;


chomp    @a;

chomp    @b;

chomp @c;

       for ($j=0;$j<scalar(@a);$j++)
          {
            @d=split(/\s+/,$a[$j]);

            $d[0]=~s/\s+//g;
```

```
for ($i=0;$i<scalar(@b);$i++)
        {
          @e=split(/\s+/,$b[$i]);
           $e[0]=~s/\s+//g;
           $e[1]=~s/\s+//g;

for ($k=0;$k<scalar(@c);$k++)
      {
        @f=split(/\s+/,$c[$k]);
         $f[0]=~s/\s+//g;
         $f[1]=~s/\s+//g;

 if (($d[0] eq $e[0] and ($d[1] eq $f[0])  and ($e[4] ne $f[4]))
              {
          print fout "$d[0]\t$e[4]\t$f[4]\n";

              }
          }
       }
    }
```
