# Supplementary material for: Development and Integration of Genome-Wide Polymorphic Microsatellite Markers onto a Reference Linkage Map for Constructing a High-Density Genetic Map of Chickpea
Source: PLoS One. 2015 May 14;10(5):e0125583. doi: 10.1371/journal.pone.0125583 (PMC4431833; doi:10.1371/journal.pone.0125583)
Supplement: S2 Table — (PDF) [file pone.0125583.s002.pdf]

Table S2: Details of 1,494 including 873 experimentally validated novel genomic and genic microsatellite markers showing *in silico* fragment length polymorphism (based on repeat-unit variation) between ICC4958 and PI489777

*Markers identity	Microsatellite repeat-motifs in ICC4958	Microsatellite repeat-motifs in PI489777	Forward primer sequences (5'-3')	Reverse primer sequences (5'-3')	Actual annealing temperature (0C)	Size (bp) of alleles amplified	Linkage groups (LGs)	Genetic positions (cM)	Markers used for polymorphism survey	Polymorphism information content (PIC)	Number of alleles amplified	Markers used in Figures	Markers types	GC%
CaGMS223	(AT)15	(AT)6	CGCTTGATAAATGGGAGGAC	GCAGCCATCAAATCAACCTT	60.0	253	1	0	NA	NA	NA	NA	NA	42.1
CaGMS506	(TA)10	(TA)6	TTGAATATTGTGTTTGATTTCCTTT	CCCTCATATTCAAAACAATCCC	58.0	219	1	4.901	NA	NA	NA	NA	NA	33.3
CaGMS433	(AT)10	(AT)6	ATTTGAAGAGCTCCGTCCAC	TGAAAACGTGGAACATCCAA	59.0	168	1	14.379	NA	NA	NA	NA	NA	42.9
CaGMS453	(ATAA)7	(ATAA)5	TCTCCTTTCCCTCTAGTAATTTGTG	TTTGTTTTAAAATTGTCACTTCAACA	59.0	272	1	14.379	NA	NA	NA	NA	NA	38.1
CaGMS462	(AT)12	(AT)8	GCGGTGAATCTTTTACGAGG	CGCGTTAACAGAGGAAGAGG	60.0	275	1	14.379	NA	NA	NA	NA	NA	42.9
CaTMS911	(TTA)31	(TTA)9	TTTGAACGGTTGACGAATGA	CGTGGAATGTAACACCTAGTCG	60.0	192	1	22.384	NA	NA	NA	NA	NA	50.3
CaGMS51	(AAT)43	(AAT)8	TCTCCTTCAACCGTCATTCC	GAAATGACCACGTGGGAGTC	60.0	220	1	25.52	NA	NA	NA	NA	NA	42.9
CaTMS873	(GAT)6	(GAT)7	CCTAATTGGGCTCAACTCCA	TGTTAACCTGCCATCAACCA	60.0	279	1	47.078	NA	NA	NA	NA	NA	50.3
CaGMS31	(AT)13	(AT)6	GGGATGCATCTGCAAATTTTA	TGAGTCCAATAAAACCCCCA	60.0	249	1	53.85	NA	NA	NA	NA	NA	33.3
CaTMS557	(TCT)9	(TCT)10	GCCATCTTCTACTTCCGCAC	AATTCGAACTCACCACTCGG	60.0	231	1	54.455	NA	NA	NA	NA	NA	50.0
CaTMS673	(TAA)6	(TAA)7	CATGGAATTCCGGTGCTACT	GACGGTGTAGCTGCATGAAA	60.0	112	1	54.655	Used	0.45	2	NA	Class II	54.0
CaTMS731	(TCT)7	(TCT)6	GAAGCATCGTCTTCCCTGAG	AATCGTTGGCGTTGTTTCTC	60.0	192	1	55.458	Used	0.65	3	NA	Class I	50.3
CaTMS776	(AG)8	(AG)13	GAACTGAGCGAGGAAGTTGG	ACATCTCCGAACTCGACCAC	60.0	165	1	60.265	Used	0.64	3	NA	Class I	54.0
CaGMS1195	(TA)11	(TA)8	TGGCCTAAAACAGTCACCTG	TCTTCATTCCCAGAGTTTTCC	59.0	253	1	62.133	Used	0	1	NA	Class I	38.1
CaGMS14	(AT)16	(AT)6	TTCACAAACAACACCACACAA	TCACACGTGTCACAGTTCCA	58.0	174	1	64.644	NA	NA	NA	NA	NA	42.9
CaTMS687	(TTA)8	(TTA)5	CCTCGCCAAATAATCTCAGG	CCGAAGAGCAGAGGAAGATG	60.0	259	1	72.847	Used	0.62	3	NA	Class I	50.0
CaTMS651	(TCT)5	(TCT)7	ACGGTCAGTGAGCTGCTTCT	TCCCAATCCTAGCTACCACG	60.0	151	1	74.487	Used	0.62	3	Figure 2H and 5A	Class II	54.0
CaTMS868	(TGA)5	(TGA)7	ATGTGCGAAGAAGAAGCACA	AGGTGGATCACTTGTTTGCC	60.0	104	1	75.348	NA	NA	NA	NA	NA	50.3
CaTMS884	(AAT)5	(AAT)6	TCACAACCACCTGTAGCAGC	TGACCCTTTTGGGGTCAGTA	60.0	253	1	87.622	NA	NA	NA	NA	NA	50.0
CaTMS1095	(AT)15	(AT)7	TGTGATTGCTTTGTTATACGATCA	CGCATGAGTGGAAACAACAT	60.0	246	1	87.78	NA	NA	NA	NA	NA	50.4
CaTMS652	(ATG)6	(ATG)5	AATGTTTCAATTTTCAATGGGA	GGATCGGTTATCGACTGAGG	60.0	189	1	88.323	NA	NA	NA	NA	NA	54.0
CaTMS594	(CTA)6	(CTA)5	AGACATCCACCACCACCATT	TGGAGAGGATCCATAGCAGG	60.0	260	1	103.692	NA	NA	NA	NA	NA	50.2
CaTMS761	(AGA)7	(AGA)6	CAGATTCCAAACGTGCAGTG	ATTGCAATGTGAACCCACAA	60.0	253	1	107.486	Used	0.69	3	NA	Class I	54.0
CaGMS1298	(TTA)8	(TTA)6	GTCTGGCCTAGCCTGACCTA	TGACTACCAAATATGGAATCGC	59.0	275	2	0	NA	NA	NA	NA	NA	45.0
CaTMS1084	(AT)15	(AT)7	ATTGTCACGACCCGAAAACT	TGGAAACGTTGAAATTACAAATGA	59.0	204	2	0.01	NA	NA	NA	NA	NA	50.0
CaTMS541	(CCA)7	(CCA)6	CCAACTAGGCAAAAGCTTCG	GTTTGCAGGTTTGAGCTTCC	60.0	273	2	3.22	Used	0.67	3	NA	Class I	54.0
CaTMS566	(CTT)6	(CTT)8	TCCTCCAACAACAACACCAA	AGGAGGAACCTTTGAAACCC	59.0	218	2	5.44	Used	0.42	2	NA	Class II	50.0
CaGMS1160	(CT)8ttc(T)10	(CT)8	TCCGTTTAATTTTGCTTGCT	CGTGCACTTCAAAACCATGA	58.0	248	2	5.916	NA	NA	NA	NA	NA	38.1
CaGMS4	(TAT)32	(TAT)5	TTCACTTGTTCAAGTACAACTATTTCA	GGAATGTGCCAACATCAGAA	58.0	273	2	15.168	Used	0	1	NA	Class I	52.6
CaGMS43	(AC)11	(AC)6	ACCGAAGAGTTTGATGGACG	GCTGCACTCATGGATGTGAC	60.0	137	2	15.329	Used	0.7	4	Figure 2S	Class I	42.9
CaGMS1125	(AT)8tgctttttattatttataa(AT)7	(AT)6	TGGAATGAAGAGATCCTCGC	CAAGTGGCAGCAGAAGTTCA	60.0	165	2	16.644	NA	NA	NA	NA	NA	38.1
CaTMS548	(ATG)7	(ATG)9	CAGGCCTTGTTTGTTGAGGT	TTCTCCTCGATTTCAATGGG	60.0	206	2	17.013	Used	0	1	NA	Class I	53.2
CaTMS871	(ACA)5	(ACA)6	GGTATTCCCCAATTTCAAGGA	TTAGCCGTTCTAGGTCACGC	60.0	277	2	17.062	NA	NA	NA	NA	NA	50.3
CaGMS22	(TTA)15	(TTA)9	TCCCTTCCCCTCTTTTGTTT	TGTTTGGAAAACTGTGTTTAGAGT	60.0	203	2	20.392	Used	0	1	NA	Class I	42.9
CaTMS788	(TGT)7	(TGT)5	CAGGAACCAAGATTGCAAGA	GGGAAAGAGTTCAAAACCCA	59.0	192	2	21.013	NA	NA	NA	NA	NA	50.0
CaGMS26	(AT)14	(AT)6	TGGAGAGGAATTGTTACGTGG	GCTTCCAATCAATAAAGTCTTACAA	60.0	222	2	21.185	Used	0.66	3	NA	Class I	33.3
CaTMS798	(TTC)5	(TTC)6	AATGCCTTCACGAATTTTGG	AGGTTACTTCCTGCGGGTTT	60.0	240	2	27.882	Used	0.4	2	NA	Class II	50.7
CaTMS913	(TTA)28	(TTA)6	GCACACAGAATTTTGGTATTCTCTT	AGCAAAAACATATTAATTGTAACAACA	60.0	245	2	31.773	NA	NA	NA	NA	NA	50.4
CaTMS759	(TTC)8	(TTC)11	GTCCCCCGCAGTTACTGTTA	GTAATTGTGAAGCCGGTCGT	60.0	218	2	34.57	Used	0	1	NA	Class I	54.0
CaTMS716	(CCA)7	(CCA)6	ATTGATGACTCCGATTTCGC	ACCAGGTTGTCTACCCCAAA	59.0	148	2	37.246	Used	0.68	3	Figure 2D	Class I	52.6
CaGMS41	(TG)11	(TG)6	GGGGGTAAAGATTTCTCCACA	TCCCATAATTCAGGGATCTTG	60.0	225	2	39.786	Used	0.69	4	Figure 2R	Class I	33.3
CaTMS690	(GAA)5	(GAA)6	GAATGGGAGGAAGTGGTTGA	CAACCCAACACCCCTCTCTA	60.0	198	2	39.897	Used	0.45	2	NA	Class II	50.0
CaTMS848	(GCA)5	(GCA)6	TCTTTGATTGCCTCTGGACC	TGTATCAAATTGGGTGGCAA	60.0	263	2	58.849	NA	NA	NA	NA	NA	50.0
CaTMS1110	(AGA)13	(AGA)8	TTCTTCAAAACCTTCTTTCCCA	AGCAGTTTAAGTCCGCTCCC	60.0	262	2	60	NA	NA	NA	NA	NA	54.0
CaTMS870	(TAT)8	(TAT)7	AATATGCGCAGGAAACCTTG	CTTCAGCACCAAAGGAAAGC	60.0	235	2	60	NA	NA	NA	NA	NA	50.3
CaTMS928	(AAT)25	(AAT)5	TTTCAAGTTTGGGGAGTTGG	TTTGCACACATTGTTGTTTGTC	60.0	258	2	60	NA	NA	NA	NA	NA	53.2
CaTMS746	(AC)6	(AC)7	ATTGCCATGAGAAATGGAGG	CCAGAAGATCCAAAGCCTGA	60.0	236	2	60.008	NA	NA	NA	NA	NA	53.2
CaTMS939	(ATT)27	(ATT)8	CGTATTTCATGTGCCTCACAA	TCCCACATCTGGAATATAGTTGTC	60.0	279	2	87.264	NA	NA	NA	NA	NA	55.0
CaTMS750	(GAA)5	(GAA)6	TGCAAGTTAACACGAGCACC	TTCCAGCAAATTTTGGAACC	60.0	189	2	94.907	Used	0.4	2	NA	Class II	50.0
CaTMS712	(AGA)5	(AGA)6	GAGGAGGTGAATTTGGCAGA	TGTTGGTTCTATTAACCCCCAT	59.0	217	2	97.218	Used	0.4	2	NA	Class II	50.0
CaTMS732	(ATG)6	(ATG)5	TTGAAGGTGGTGATGGTGAA	GGGCACCTTATTAGCCCATT	60.0	169	3	16.732	NA	NA	NA	NA	NA	50.4

*Markers identity	Microsatellite repeat-motifs in ICC4958	Microsatellite repeat-motifs in PI489777	Forward primer sequences (5'-3')	Reverse primer sequences (5'-3')	Actual annealing temperature (0C)	Size (bp) of alleles amplified	Linkage groups (LGs)	Genetic positions (cM)	Markers used for polymorphism survey	Polymorphism information content (PIC)	Number of alleles amplified	Markers used in Figures	Markers types	GC%
CaTMS927	(AAT)27	(AAT)7	TTTGGAAGTTTGGAAGTTGCT	GGGTGAGGTGGTCCAATAAT	59.0	280	3	19.499	NA	NA	NA	NA	NA	53.2
CaTMS830	(GAT)9	(GAT)11	ATGATGCTACTGGAGGTGGC	CCCAAATCCTCCTCTTCCTC	60.0	197	3	26.877	NA	NA	NA	NA	NA	53.3
CaGMS34	(TGG)10	(TGG)6	GGTTTTGAGAGAGAGTGCGG	TCTTCCGCAAAACAAAAACC	60.0	203	3	30.349	NA	NA	NA	NA	NA	42.9
CaGMS24	(ATAA)9	(ATAA)5	TTTGGTTGCTAGACCAAGGG	CGGAATCCGATTGCTTCTAC	60.0	150	3	31.897	Used	0.67	4	Figure 2Q and 4A	Class I	38.1
CaTMS581	(TCA)6	(TCA)8	AACACTTGAACATTTCAAACC	ATGGTGATGTTGTTACCAAAG	55.0	153	3	32.203	NA	NA	NA	NA	NA	50.4
CaTMS579	(CT)8	(CT)9	TGGAGCCCTACATTATCGGA	GCAAGAGCACAGACACTCCA	60.0	156	3	32.493	Used	0.42	2	NA	Class II	50.7
CaGMS1164	(AG)9(GT)6	(AG)8	TAGGTTTGTTGTGTGGGTCG	CCCTCACTCAGCGGTCTATC	59.0	142	3	32.963	NA	NA	NA	NA	NA	47.6
CaTMS1053	(AT)16	(AT)6	TGCAAATGTATAATTTTGTGAGACA	TTGCGTCAACAACTTTCCAG	59.0	266	3	33.75	NA	NA	NA	NA	NA	50.0
CaTMS646	(ACA)6	(ACA)5	CCCAGATACAATGCATACGCT	CCGATTTTCCTTCCTCAACA	60.0	222	3	34.078	NA	NA	NA	NA	NA	50.0
CaTMS1007	(ATT)15	(ATT)5	CGGTTACCTATTTTTAATTGTGC	TTCTCCATCACACTGGGAGA	57.0	247	3	34.143	Used	0.67	3	NA	Class I	51.5
CaGMS1254	(TA)11	(TA)8	GAACCCCTTTCAACAAACGA	CGAGCCTCGTAAAATCCAAA	60.0	158	3	36.822	NA	NA	NA	NA	NA	42.9
CaTMS553	(TCC)6	(TCC)5	TCATCTGGGAAAAGGGAGTG	AAGGACCTCATCAAAACCAGAA	60.0	195	3	38.884	Used	0.41	2	NA	Class II	50.0
CaTMS670	(TTC)13	(TTC)5	ATGAAATTGCTCCGTTGAGG	GGGATTTGATTCGTGGAAGA	60.0	258	3	38.89	Used	0	1	NA	Class I	53.3
CaTMS859	(TCT)10	(TCT)8	GATCAGATGCGGCAATATCA	TAGAGGGGAACAGCAGAGGA	60.0	173	3	39.217	NA	NA	NA	NA	NA	50.4
CaTMS625	(AG)6	(AG)7	CAGAGACCAAGGGAAGCAAG	ACAATACGGCAACAAGAGGG	60.0	267	3	40.134	NA	NA	NA	NA	NA	52.8
CaTMS1062	(TAA)11	(TAA)5	TTGTTAACGTGAAGTTACGATATGC	TCTGACCAATTTTGTACCCCA	60.0	279	3	40.64	NA	NA	NA	NA	NA	52.4
CaTMS989	(TAT)24	(TAT)13	TCACAACATTTCAATTAGAGGAAAA	TGCACAGTAGAGATGATATAGCGA	59.0	147	3	40.694	NA	NA	NA	NA	NA	50.0
CaTMS665	(TGG)7	(TGG)6	TCTAGGCCTGGAAAAATGGTT	TGATTCCCATTTCCTCTTGC	60.0	179	3	40.71	Used	0.7	3	NA	Class I	54.0
CaTMS662	(TTA)6	(TTA)5	CTGGGTGGGACCATTTATTG	GCAACACACACATGCCCTAC	60.0	264	3	40.722	NA	NA	NA	NA	NA	54.0
CaTMS826	(GA)10	(GA)7	CTGGTCCAAAGATCACAGCA	CTTTCACCCCCACAAACACT	60.0	240	3	40.722	NA	NA	NA	NA	NA	54.0
CaTMS645	(TTC)10	(TTC)5	ACCATTTCCTCCTGTGTTGC	CATAGCCACAGCCATTGAAA	60.0	247	3	41.199	NA	NA	NA	NA	NA	50.0
CaTMS971	(TTA)22	(TTA)6	AGCTGCATTGGGACAAACTT	AAGCCACGAGACAATGGCTA	60.0	266	3	42.216	NA	NA	NA	NA	NA	50.3
CaTMS956	(TAA)26	(TAA)8	TTTTTAAACCCTCTCATTGAATTT	ACGTGAAAATTTCGGAATCG	57.0	277	3	42.297	NA	NA	NA	NA	NA	55.0
CaGMS28	(TTA)13	(TTA)8	ACCCAAGAAGCGAAAAAGGT	CCAAGCCCAATAAACCACAT	60.0	230	3	42.352	NA	NA	NA	NA	NA	33.3
CaTMS592	(TC)10	(TC)11	CGCAACTGTTTTGACGTTGT	CCACCGCTGGACATTAACTT	60.0	241	3	42.366	NA	NA	NA	NA	NA	50.3
CaTMS836	(CAG)7	(CAG)6	TGGCATCATCATCACTGTCTT	CCATCCTTTTCAATGCGAGT	60.0	253	3	42.419	NA	NA	NA	NA	NA	50.0
CaGMS1290	(GAA)7	(GAA)5	AACGACAAAATTGAAGCCCT	TTGGAATTTTGAGCTTGAGTTG	59.0	121	3	42.424	NA	NA	NA	NA	NA	38.1
CaTMS1016	(TTA)13	(TTA)5	TCTGAACAAGAATTAGAGGCAAAA	TCCATCCATTACGTCTGTTGA	59.0	215	3	42.424	NA	NA	NA	NA	NA	50.0
CaTMS1021	(AT)18	(AT)6	CAACACTTGATGTTAGATTGGACA	CGACAAAAACGAAAATTAAGGG	59.0	150	3	42.424	NA	NA	NA	NA	NA	50.0
CaTMS1077	(TA)14	(TA)6	CCTATACTTTGGTTTGGTCCATT	CCAGCAAGCAAGTCGGTTTA	58.0	206	3	42.424	NA	NA	NA	NA	NA	53.2
CaTMS611	(CAA)7	(CAA)8	GCATCAAATCCACACCCTTT	TTCGAGACTCGGTGGAAACT	60.0	201	3	42.424	NA	NA	NA	NA	NA	50.0
CaTMS948	(AAT)24	(AAT)6	TTCATTTGAGATTTAATACGACATTTT	GAGGAACAACAACTGCAATTAAAC	59.0	273	3	42.424	NA	NA	NA	NA	NA	50.0
CaGMS1270	(GA)18	(GA)15	GTTAGGGGCCCATTTTGAAC	GATGGCTTCACGACCTTGTT	61.0	210	3	42.424	Used	0.65	4	NA	Class I	38.1
CaGMS1304	(TC)8	(TC)6	CGATCAGTTAAAACCGCACA	TCTTCGACGAAACAAAGCAA	60.0	241	3	42.424	Used	0.43	2	NA	Class II	38.1
CaTMS610	(AG)7	(AG)9	AAAATTGGATTGGGAAAGGG	TTTGCTACCAAACCAAACCC	60.0	131	3	42.424	Used	0.4	2	NA	Class II	50.0
CaGMS1165	(AC)6cct(C)15	(AC)6	AAACGGATCAATGCGAGAAG	CATTACATTGTCATTTGGCCC	60.0	279	3	42.431	NA	NA	NA	NA	NA	52.4
CaGMS1220	(TC)9	(TC)6	CCATGGAAATGCTGCTGATA	TCAACACCCCTAACTCGGAC	60.0	269	3	42.431	NA	NA	NA	NA	NA	42.9
CaTMS1093	(AT)14	(AT)6	AGTGCCTTTTGCATACACACA	CTGGTTGGGAGAATTTTTGC	59.0	198	3	42.431	NA	NA	NA	NA	NA	50.0
CaTMS947	(TTA)27	(TTA)9	AAAACATCTTCAATAAAAACCATGC	AAATAATTTTAAACCAACGGCA	60.0	151	3	42.431	NA	NA	NA	NA	NA	50.0
CaTMS943	(ATT)24	(ATT)5	CACAAGGGTATAGACGGCAG	TGCACATGTCAAATCAAATTCA	58.0	203	3	42.435	NA	NA	NA	NA	NA	52.4
CaTMS605	(TAT)7	(TAT)5	CGCCACAACAACACCATATC	CGAAAGAAATTGATGCGAGA	59.0	202	3	42.435	Used	0.71	3	NA	Class I	50.0
CaGMS1166	(CT)8(CA)7	(CT)8	CGTACGGAGTGTTATTGGACTG	GGATTTCATCCTGGTTTTAGCTC	60.0	186	3	42.436	NA	NA	NA	NA	NA	38.1
CaTMS1072	(TAT)13	(TAT)7	TCCATGATTGCAATTTGCTC	CCACATTAGTCAATATGAGTTTCACC	60.0	134	3	42.436	NA	NA	NA	NA	NA	52.6
CaTMS886	(TGG)8	(TGG)6	TGGAGTTACACCCTTCCTGG	GGCTCTGCAAGATCACCTTC	60.0	159	3	42.436	NA	NA	NA	NA	NA	50.0
CaTMS897	(TTA)31	(TTA)6	TTGCCTTTTAAATTTTCAATCAAT	AAAAAGGAGAATGATACTTTTATGACA	58.0	231	3	42.436	NA	NA	NA	NA	NA	50.0
CaTMS907	(TTA)30	(TTA)7	TACGGGCAAGTTTATACGGG	TCTGTGTACCTGTTATGGGCA	60.0	248	3	42.436	NA	NA	NA	NA	NA	50.0
CaTMS1073	(CTT)13	(CTT)7	GGTCTTCCAACACACCCATC	ACCAAATGAAAAGCTGGTGG	60.0	218	3	42.437	NA	NA	NA	NA	NA	52.8
CaTMS509	(CAA)6	(CAA)7	TCATGGGACTCTGTCATGGA	GAGAAGGAGGTGGAAAAGGG	60.0	279	3	42.441	NA	NA	NA	NA	NA	50.0
CaGMS1130	(AGA)8	(AGA)7A(GAT)5	GATCTTCACCTGCACCCATT	GAGGGACAGCGATACCATGT	60.0	264	3	42.443	NA	NA	NA	NA	NA	42.9
CaTMS1028	(AT)18	(AT)7	TGAGTGCAAAGTTAACAATCTCC	TGAATTTAACTCCGATCATCAA	58.0	236	3	42.443	NA	NA	NA	NA	NA	51.2
CaTMS1120	(TTA)12	(TTA)7	TGTCTCGATAAGAGTTTGTTATTTTTC	CGTTTTGTTTCATATTCAAACTCG	59.0	220	3	42.443	NA	NA	NA	NA	NA	50.0
CaTMS935	(ATA)24	(ATA)5	CCTAACAACCTCACAACCCAA	CAAACAGAGAAACACTTGTTCAGG	60.0	257	3	42.443	NA	NA	NA	NA	NA	55.0
CaGMS1205	(TCT)10	(TCT)8	TCGTCCAAGCAAACACATTC	GGTTCTGCCGAGTATTCCCT	60.0	261	3	42.449	NA	NA	NA	NA	NA	38.1

*Markers identity	Microsatellite repeat-motifs in ICC4958	Microsatellite repeat-motifs in PI489777	Forward primer sequences (5'-3')	Reverse primer sequences (5'-3')	Actual annealing temperature (0C)	Size (bp) of alleles amplified	Linkage groups (LGs)	Genetic positions (cM)	Markers used for polymorphism survey	Polymorphism information content (PIC)	Number of alleles amplified	Markers used in Figures	Markers types	GC%
CaGMS1317	(GA)8	(GA)6	CCAGCTGCTTTTCATGGGTA	TTGGCTCAGTGCATTGCTAA	61.0	276	3	42.453	NA	NA	NA	NA	NA	31.8
CaGMS1171	(GT)9(AT)6	(GT)8	AAGACATTATGAAATGCAACCAAA	TGAGAGATGCATTCAACATTATAGG	60.0	250	3	42.456	NA	NA	NA	NA	NA	47.6
CaGMS27	(AAT)10	(AAT)5	TGGAAGGGCCATACCTATCA	GGCTGCCTTTGTCGTAGTTC	60.0	177	3	42.458	Used	0	1	NA	Class I	33.3
CaGMS1178	(TAT)21	(TAT)19	CGTCAAAATTCATGCAGTCG	GGGACTTTCTGAAGCCGTTT	60.0	199	3	42.462	NA	NA	NA	NA	NA	47.6
CaTMS540	(TTC)7	(TTC)5	TTTCCATCCACAGAAGAGGC	AATGAGGATGATGCAGAGGG	60.0	126	3	42.484	NA	NA	NA	NA	NA	50.0
CaTMS849	(GAA)8	(GAA)9	GGCCACAGCAGTCATCTACA	CACCTCATCGATCAGCTTCA	60.0	172	3	42.49	NA	NA	NA	NA	NA	50.0
CaTMS1063	(TAA)12	(TAA)6	AAAAATTAGTTTTGGTAATTGTTGG	GGAGGTGATGGTGGCTAGAA	57.0	218	3	42.494	NA	NA	NA	NA	NA	52.4
CaTMS643	(CAC)7	(CAC)9	AACCCATTTTGCAATCTGCT	CGCTGAGGAGAGAGTTCCAC	60.0	124	3	42.497	Used	0	1	NA	Class I	53.7
CaGMS1319	(TC)12	(TC)10	CATGCAATCACACCAACACA	CTGTCCTTCAACACCTGCCT	60.0	209	3	42.502	NA	NA	NA	NA	NA	38.1
CaGMS1256	(CT)9	(CT)6	AAAGCAAAGAAGACCCCTCC	TTTGGGATGCAAATTGAACA	60.0	221	3	42.503	NA	NA	NA	NA	NA	33.3
CaTMS1030	(TTA)13	(TTA)6	TGCACTTGGAAGAGATAGTTGTT	CAATATTAGAAATCATGGTTGAGGT	58.0	247	3	42.503	NA	NA	NA	NA	NA	51.5
CaTMS1083	(AT)14	(AT)6	AATCTTCCGAGGCATTGTTG	GAGGTTCGGTGCGGTAGATA	60.0	102	3	42.503	NA	NA	NA	NA	NA	50.0
CaTMS1103	(ATTT)9	(ATTT)5	GCTTCAGAATTCGACGTGGT	TACATTGGGCTGGTACATGG	60.0	105	3	42.503	NA	NA	NA	NA	NA	50.0
CaTMS533	(AG)6	(AG)7	TCCACACAGACATGGTTCATC	CCTCATGGAAGGCAACCTTA	60.0	207	3	42.503	NA	NA	NA	NA	NA	50.2
CaTMS578	(TTC)12	(TTC)5	ATCACTTCTTGCCATGTCCC	GGTGGTGGAATGAGGAGAAA	60.0	106	3	42.503	NA	NA	NA	NA	NA	50.7
CaTMS659	(ACA)5	(ACA)6	ACTGTTGACCCAAAAGTGGC	TCCATGTGAAACTGGTTGGA	60.0	216	3	42.503	NA	NA	NA	NA	NA	54.0
CaTMS669	(AAG)10	(AAG)5	GCTTGTGCATGGAAGACAAA	GCGAGTGATAAATACTGCCCA	60.0	266	3	42.503	NA	NA	NA	NA	NA	53.3
CaTMS777	(GAA)6	(GAA)5	AAACGCAAGCAGAGAAGGAA	CCTGGCAATCGTTTGACTTT	60.0	173	3	42.503	NA	NA	NA	NA	NA	53.2
CaTMS778	(AG)7	(AG)8	AGAGTCAAATTAACAAAGAGCATAGA	AAAATGCCCACAAGAGCAAG	60.0	122	3	42.503	Used	0.42	2	NA	Class II	53.2
CaGMS1274	(TA)9	(TA)6	CATTCCACCTTAAAACTCCTCG	CCGTCTAACTAAGATGAACCCTG	60.0	199	3	42.509	NA	NA	NA	NA	NA	33.3
CaGMS1291	(AT)9	(AT)6	CAGTTCGATCACATACTAACACCA	TGCTCTCGCCAAACTAACAA	59.0	279	3	42.509	NA	NA	NA	NA	NA	42.9
CaTMS602	(TC)12	(TC)10	TGACGTTGGGTGGGTATGTA	CAACAATGGCACGATGAGAG	60.0	158	3	42.509	NA	NA	NA	NA	NA	50.0
CaTMS607	(TTC)6	(TTC)5	GCAGCAACATCTGGTGAGAA	CGTTCAGAGGTCTAGGGTGC	60.0	205	3	42.509	Used	0.4	2	NA	Class II	50.0
CaGMS1131	(AG)6(A)12	(AG)6	TTGAATCGAAATGGGTGTGA	CCCCTTTGCCTTTATTCCAT	60.0	197	3	42.51	NA	NA	NA	NA	NA	33.3
CaTMS1014	(TTA)22	(TTA)14	CAGATTTGCTGGTAGGTGACAG	AACCTCCAAATGCAAAATGA	60.0	247	3	42.517	NA	NA	NA	NA	NA	50.0
CaTMS570	(TTCATT)6	(TTCATT)7	AAACACCAGCAGAAGCCAAT	CACGATGACACCCAAATCAC	60.0	229	3	42.52	NA	NA	NA	NA	NA	50.0
CaTMS808	(TCT)5	(TCT)6	ACAACTCCGTTTTGGAATCG	GAGCTGTTTCAAGAGCCGAC	60.0	229	3	42.52	NA	NA	NA	NA	NA	51.8
CaTMS587	(ACT)6	(ACT)7	CAAAACCCCATAATGCCACT	TTAGCTGAGCTGTTTGGCG	60.0	205	3	42.52	Used	0.45	2	NA	Class II	50.4
CaTMS1019	(TAT)13	(TAT)5	AGTAAAGGGTTTTAAGATGATGGA	TTTTCTTTCTTTACGGTCTTAGAACA	57.0	241	3	42.529	NA	NA	NA	NA	NA	50.0
CaTMS881	(GAA)6	(GAA)5	TCAAAAACAAAATGGGTCCAA	CAAGGGAAGTAGCAACGAGG	60.0	207	3	42.529	NA	NA	NA	NA	NA	50.0
CaTMS967	(ATA)29	(ATA)13	CCTTAACAGCCCCAGATTGA	TTTTCATCAAAACTTTAATTTGAGAG	60.0	239	3	42.529	NA	NA	NA	NA	NA	50.3
CaTMS845	(TC)7	(TC)8	AGCTTCTTTTGGCTTGTGGA	GAAGCATGTTTGCTGCAACT	59.0	266	3	42.534	NA	NA	NA	NA	NA	50.0
CaGMS1267	(AT)9	(AT)6	TGTGAACTTTTCATTTGGCTTG	CCATTCGATCATGGGTAGTAGTT	60.0	205	3	42.537	NA	NA	NA	NA	NA	42.9
CaTMS1070	(AT)15	(AT)6	CTGACCACTGGTTTGCAGTC	CAAAAATTGGTTGCCCAAAG	59.0	119	3	42.539	NA	NA	NA	NA	NA	50.4
CaTMS771	(AG)12	(AG)13	CAACACAAGCAGCAACGAAC	GGCATGGCCTTCATCTTAAA	60.0	169	3	42.541	NA	NA	NA	NA	NA	54.0
CaTMS1097	(AT)15	(AT)7	AAACGGAAAGTCAAATTGAATACAT	GGAGCTATCAACTTTCAAATTCTCA	59.0	226	3	42.657	NA	NA	NA	NA	NA	53.9
CaTMS1079	(AT)14	(AT)6	CAAACATAATCACCGCCTCC	CGATGAAAACAAAGGAAAAACA	60.0	218	3	42.662	NA	NA	NA	NA	NA	53.2
CaGMS1129	(AT)6(AG)6	(AT)6	TCCGAGAAGCATCATGAAAA	TGGAGGAAAGTGGTGCTTCT	59.0	131	3	42.949	NA	NA	NA	NA	NA	42.9
CaTMS628	(CTT)6	(CTT)5	TTCTCTTCCTCCATCAATGACTT	GCGAAAAGTTGGTTGGAAAA	60.0	155	3	43.093	Used	0.45	2	NA	Class II	53.1
CaTMS814	(AG)26	(AG)10	GAAAAGCGGAGAAAGTGTGC	AAACTCTCTCTCCCACGCCT	60.0	176	3	43.396	NA	NA	NA	NA	NA	50.0
CaTMS793	(AG)7	(AG)6	GCTATCGCCGATCAAGAGAA	TTCTAAGCTTTGGAAGAGATCCA	60.0	236	3	43.46	Used	0.4	2	NA	Class II	50.3
CaTMS730	(AG)12	(AG)10	TGAACCTTGTTTGGGGTCTC	TCCTCCCATTCTTTGCATTC	60.0	136	3	46.196	NA	NA	NA	NA	NA	50.0
CaTMS781	(CT)10	(CT)11	TGTCCCCATCTTCTTCATTCA	TTATTCAATGGGGTTCCAGC	60.0	241	3	47.696	Used	0.42	2	NA	Class II	50.0
CaTMS683	(TAT)6	(TAT)8	CCAGTTGTTGCCTCGGTATT	CAGGTTGATGTCCGAATGTG	60.0	273	3	48.044	Used	0	1	NA	Class II	50.0
CaTMS780	(GT)9	(GT)7	TTTTGTTAGCGTTTGTGTTTGG	CATAAAACCGCCTGCAACTT	60.0	203	3	48.143	Used	0.38	2	NA	Class II	53.3
CaTMS552	(CAG)5	(CAG)6	TCATGCCATGCACACTCTTT	CTGCTGCATTTGTTGCTGTT	60.0	154	3	48.179	NA	NA	NA	NA	NA	50.0
CaTMS654	(CAT)9	(CAT)6	TCGCATAGAAGATGTCGTCG	ACGCTACTCGATGACGAGGT	60.0	152	3	48.556	Used	0.64	3	Figure 2B	Class I	54.0
CaTMS914	(TTA)31	(TTA)10	CGTGAAATGTGACCATAAAAACA	TTACCATCAATTTGAGGAATTTTT	60.0	226	3	48.802	NA	NA	NA	NA	NA	50.7
CaGMS39	(AT)13	(AT)7	CCGCAATCATATCAACTTTGTG	CCCTTCCAATTCTTCAATCC	60.0	243	3	49.301	NA	NA	NA	NA	NA	38.1
CaTMS710	(ATTC)5	(ATTC)6	AGAATTCACAACCCACCGAC	ACATCACCTCCTCCGTTCAC	60.0	240	3	49.948	NA	NA	NA	NA	NA	50.0
CaTMS999	(TTA)15	(TTA)5	TGTTTGGTTTTCATTCTTAGCA	AAATGTCATATGAGCCCTCCTC	58.0	251	3	50.716	NA	NA	NA	NA	NA	55.0
CaGMS1318	(TA)8	(TA)6	CGGGTAATGAAACTCATAAATTAAA	CAATGCCTGCATTCCAATTA	58.0	274	3	51.204	Used	0.39	2	NA	Class II	42.9
CaTMS629	(CTT)6	(CTT)5	ACCATTTGGCATTGTTCCTC	GCGAAACCTGGAGTTCTTGT	59.0	266	3	51.44	Used	0.35	2	NA	Class II	53.2

*Markers identity	Microsatellite repeat-motifs in ICC4958	Microsatellite repeat-motifs in PI489777	Forward primer sequences (5'-3')	Reverse primer sequences (5'-3')	Actual annealing temperature (0C)	Size (bp) of alleles amplified	Linkage groups (LGs)	Genetic positions (cM)	Markers used for polymorphism survey	Polymorphism information content (PIC)	Number of alleles amplified	Markers used in Figures	Markers types	GC%
CaTMS863	(TTC)9	(TTC)6	TTCTCCAATTTCTCCCTTTTGA	AATTGAGCCTTTTGCCATTG	60.0	100	3	51.73	NA	NA	NA	NA	NA	50.4
CaGMS1297	(ACA)7	(ACA)5	GCCAACCATGTCTGCTCATA	CCACGTGATCAATCCCTTCT	60.0	171	3	51.924	NA	NA	NA	NA	NA	33.3
CaTMS1122	(TAT)10	(TAT)5	AAAAATACAGAAAACTGAAGGAAAAA	CGGCAAAATAGCAGGAAGAC	58.0	246	3	52.701	NA	NA	NA	NA	NA	54.0
CaTMS1089	(TA)15	(TA)7	CGAAGAGACATGACTTTGTCCA	TTTAGAGCTGATCATTCAAAAAGA	60.0	211	3	53.028	NA	NA	NA	NA	NA	53.7
CaTMS949	(AAT)24	(AAT)6	TCACCTTCATTGTTGGTCGT	TTGTAAATTGTGGCACCCAG	59.0	256	3	53.654	Used	0	1	NA	Class I	50.3
CaGMS1134	(TG)7(TA)6	(TG)7	CTGGAGCCGAAAGAGAAAAA	AGCGAGTTGTTCAGCTTGTG	60.0	274	3	54.222	NA	NA	NA	NA	NA	33.3
CaTMS1015	(AAT)19	(AAT)11	AGTTCAATGTTTTGTAAGTCGTGT	TTTTTGATTTTCTGTACAAGCATTT	57.0	278	3	54.222	NA	NA	NA	NA	NA	50.0
CaTMS852	(TC)14	(TC)17	TTCCGTTTTCAAACCCAAAC	AACAGAGAGCTCCGAATCCA	60.0	139	3	54.222	NA	NA	NA	NA	NA	50.0
CaTMS926	(AAT)25	(AAT)5	CGGTTCACTACTAAATCCAACCA	TGTGTTGTGTAACACCCCTGA	60.0	261	3	54.222	NA	NA	NA	NA	NA	53.2
CaTMS995	(TAT)20	(TAT)10	CAAAAGACTCACCTTGACAACA	TGGGCCTATGTGACACCTCT	58.0	192	3	54.222	NA	NA	NA	NA	NA	50.0
CaGMS45	(CT)15	(CT)10	CACATGTTCTTGCAACCCTG	ATTCAAAACCCAGAAAGCCC	60.0	154	3	54.222	Used	0.62	4	Figure 2T	Class I	33.3
CaTMS1059	(AT)16	(AT)6	AAAATATTTCCTCCCGTCCC	AAAACAAAAATCACCAAAAACGA	59.0	195	3	54.23	NA	NA	NA	NA	NA	50.0
CaTMS660	(CAG)6	(CAG)5	GCCTTCCAATCCAATCTCAA	AAGCAGACCTTCCACTTCCA	60.0	164	3	54.231	NA	NA	NA	NA	NA	54.0
CaGMS1282	(CT)9	(CT)6	AAACTCCCGAATCAAACACG	GTGAGGTATGTCGATGCGGT	60.0	161	3	54.249	NA	NA	NA	NA	NA	38.1
CaTMS525	(CAT)5	(CAT)7	TCACCATGCCATTAACTCCA	GTGCCAGGAGAAGGTCTCAG	60.0	267	3	54.249	NA	NA	NA	NA	NA	51.8
CaTMS512	(CAG)7	(CAG)6	CGATTGGGGTGTAAATGGAG	TACATCTGCTCCACCATCCA	60.0	273	3	54.266	NA	NA	NA	NA	NA	50.0
CaTMS753	(AAC)5	(AAC)6	TTTGTGGTGAAACATGGTGG	GCTGATTCACTTTGTTGTGGC	61.0	259	3	54.266	NA	NA	NA	NA	NA	54.0
CaTMS964	(TAT)26	(TAT)10	CAATCTTTTAGAAATAGAAATGCACAA	TCCTGGACCATAATTGAGAAA	59.0	279	3	54.283	NA	NA	NA	NA	NA	55.0
CaGMS1143	(AG)7(A)13	(AG)7(A)11	CATAAATGGTTGCGACATGC	TCGACACAAACAAAATCACCA	60.0	168	3	54.287	NA	NA	NA	NA	NA	33.3
CaGMS1312	(AT)9	(AT)7	TTGAACCCCAAATTCTCAGC	AAAGATGGTCCATAGTGAAGCAC	60.0	237	3	54.572	Used	0.4	2	NA	Class II	33.3
CaGMS1227	(CA)9	(CA)6	CACTTACCCATTGCTTCCGT	GCATGTTCTCTTGAATTTCATTTT	60.0	261	3	54.589	NA	NA	NA	NA	NA	38.1
CaTMS601	(ATA)6	(ATA)5	ACCGGCCTGAACTCTACCTT	GACGCTTGAAAAGAGGATGG	60.0	249	3	55.179	NA	NA	NA	NA	NA	50.0
CaTMS811	(TAA)5	(TAA)8	GTGTTGGAGTTGGAGGAGGA	CTTGTTGCTGTTGTGCACCT	60.0	159	3	56.703	NA	NA	NA	NA	NA	50.2
CaTMS580	(ATT)6	(ATT)5	TCTCTCCCACAAAGGTCCC	GAAGGTGGCCAAGAGATGAA	60.0	134	3	57.06	NA	NA	NA	NA	NA	50.7
CaTMS691	(GAA)11	(GAA)7	CAAAGTAAACCCCACCGCTA	ACCGCAATATGGAACACGAT	60.0	259	3	57.568	Used	0	1	NA	Class I	50.0
CaTMS743	(TAA)7	(TAA)12	AATACGCATCCAATCCATCC	GTGTGGTGCTGCACAGAGTT	60.0	267	3	57.682	Used	0	1	NA	Class I	50.7
CaTMS783	(GAT)8	(GAT)6	GAGCATCATAAGCGACGTGA	CCGCCAATCATATCCATTCT	60.0	140	3	57.785	Used	0.67	3	Figure 2G	Class I	50.0
CaTMS890	(TAA)7	(TAA)8	AATGGAGCTTGAATCTGGGA	TGGGAAGCTTGTTGAAGTGA	59.0	151	3	58.763	NA	NA	NA	NA	NA	50.0
CaTMS915	(TAT)33	(TAT)12	TTTGGACAGAAAACAGTGTCATTT	CTGCATCGTGCTTTGATGTT	60.0	254	3	58.969	NA	NA	NA	NA	NA	52.6
CaTMS522	(CTT)6	(CTT)5	ACATTCCCATTCCCATTGAA	ATTTGGAATCCATGGTGCTC	60.0	215	3	61.902	Used	0.42	2	NA	Class II	51.5
CaGMS218	(AT)15	(AT)6	GGGGTGCTCAACTTGTTTGT	ACGTGGACAAGGTAAGGGTG	60.0	258	3	62.093	NA	NA	NA	NA	NA	38.1
CaTMS635	(TA)8	(TA)6	TTGCTTTGAAGAAGGGGAAA	CCACTGCTGTCCTACACTGC	59.0	155	3	63.276	Used	0.46	2	NA	Class II	53.3
CaTMS667	(GAT)5	(GAT)6	CGATCATGGCCTAAGATGGT	GGTGTTATGGGATGTTTGGG	60.0	224	3	63.36	NA	NA	NA	NA	NA	53.7
CaGMS1214	(TA)8	(TA)6	GGAGTTGGATTCTCTGGTGC	AATGGAACAAGTGGCTTTGC	60.0	240	3	64.26	Used	0.3	2	NA	Class II	38.1
CaTMS955	(ATA)27	(ATA)9	TTGATGAAGTATAGGGACATCAAAA	TTTCAATTAAAGAGACCAGATAGGA	59.0	276	3	64.388	NA	NA	NA	NA	NA	52.8
CaTMS562	(AAC)6	(AAC)5	TCCTTTTGGTGACACTACAAATACA	AAAAAGAGACGCCATTCACG	60.0	277	3	68.869	Used	0	1	NA	Class II	50.0
CaGMS1201	(AT)9	(AT)6	AACTGAGAATGACTTGCCTTCA	TTTAGGGATGATTGCTTGGG	59.0	228	3	73.385	Used	0.43	2	NA	Class II	42.9
CaTMS969	(TAT)21	(TAT)5	TGCTCTCTATTCGTTCCCTTTC	TCCGTTTAAGTTTCACTGCATTT	60.0	278	3	73.416	NA	NA	NA	NA	NA	50.3
CaGMS13	(CT)20	(CT)8	TTTGGTGGTGAACGTTGAAA	GGAGAAGGAAAAGGAAGGGA	60.0	243	3	73.65	Used	0.68	4	Figure 2K	Class I	47.6
CaTMS724	(AG)10	(AG)6	TGAACCCAACAAGGAACCAT	CCCCATGTGCTAAAAGCAAC	61.0	223	3	74.296	Used	0	1	NA	Class II	53.3
CaTMS616	(TAA)7	(TAA)5	CGCATCTTCAATTCCATCCT	GGGCCCAACTCAAAAGTTTC	61.0	159	3	74.51	Used	0.67	3	Figure 2A	Class I	52.4
CaTMS876	(TC)10	(TC)9	TACCCCCATTTGCATTTCAT	CACCAATAGTGGGGGAAAGA	60.0	237	3	74.514	NA	NA	NA	NA	NA	50.2
CaTMS519	**(AG)6**	**(AG)8**	**TTTTGGAGCATTAGGGCTTG**	**CAGCATCGCAAAGAACAGAA**	**60.0**	**271**	3	74.515	NA	NA	NA	NA	NA	50.7
CaTMS832	**(CAA)6**	**(CAA)8**	**CCATCACCACCAACCCTAAC**	**AGAAGAGAGGGCGGAGTAGG**	**60.0**	**215**	3	75.138	NA	NA	NA	NA	NA	53.3
CaTMS932	(TTA)28	(TTA)9	AGCAGATTGTCACTCTCTCCTTT	TCAGAGCATGTTCTATCAACCAA	59.0	243	3	80.916	NA	NA	NA	NA	NA	55.0
CaTMS543	(AAG)8	(AAG)6	TCGAGGATCTGATGAAGGAAG	TTCAGTTTCACGCAATCACC	60.0	236	3	80.929	NA	NA	NA	NA	NA	54.0
CaGMS490	(TA)10	(TA)6	TGAAAATTAACGTACGGCACA	GATGACTTGTTCTCGTCGCA	59.0	153	3	87.361	NA	NA	NA	NA	NA	33.3
CaTMS866	(TGG)8	(TGG)7	ATGCCTACCGGAAAGGTTCT	TATCTAGCCCACGCACATTG	60.0	150	3	91.318	NA	NA	NA	NA	NA	50.3
CaGMS5	(AAT)27	(AAT)5	TGAAAGTGTTTTGAAAATTAAATGATG	GTGGCTACGGAAGTCTCCAA	60.0	255	3	98.374	Used	0	1	NA	Class I	38.1
CaTMS815	(GTG)6	(GTG)8	TGATGATTTTGTGATGGTTTTGA	GCTGCATCACAGAAAGTTGG	59.0	224	3	98.404	NA	NA	NA	NA	NA	53.3
CaGMS7	(TTA)23	(TTA)5	TTTAGGATTAGCATCACATACAACA	TATCTGGGTGTGCTGGACAA	58.0	198	3	99.251	Used	0	1	NA	Class I	38.1
CaTMS721	(TTG)6	(TTG)5	GGGTCATTTTGTTGCCTCAT	AATTTTGTGCATGACCCTGA	59.0	143	3	99.364	Used	0.46	2	NA	Class II	53.2
CaGMS1199	(TC)14	(TC)11	CGGCCATTGAAAACGAATAC	TGGGTATTGGACTCCTTTCCT	60.0	266	3	99.962	Used	0	1	NA	Class I	33.3

*Markers identity	Microsatellite repeat-motifs in ICC4958	Microsatellite repeat-motifs in PI489777	Forward primer sequences (5'-3')	Reverse primer sequences (5'-3')	Actual annealing temperature (0C)	Size (bp) of alleles amplified	Linkage groups (LGs)	Genetic positions (cM)	Markers used for polymorphism survey	Polymorphism information content (PIC)	Number of alleles amplified	Markers used in Figures	Markers types	GC%
CaGMS1258	(ATA)7	(ATA)5	CAAAGGGGAAGAAGTGTTGC	GCCATTTCTGGAGTTGGAAA	60.0	219	3	100.626	Used	0	1	NA	Class I	33.3
CaTMS959	(AAT)27	(AAT)9	CGGAAAAGCATTAGGTGGTC	TTGATGGAAATTTCTTCTTGCAT	60.0	269	3	106.561	NA	NA	NA	NA	NA	55.0
CaTMS805	(TCT)7	(TCT)8	TTCAATCTCTTCACGCCCTT	GAAGGTTTCGAGTGCCTCAG	60.0	160	3	109.282	Used	0	1	NA	Class I	51.2
CaGMS1212	(AAG)8	(AAG)6	GAACCAAACATGCACAATGC	TCCTGCGTATCTGTGTCTTTGT	60.0	278	3	119.867	Used	0	1	NA	Class I	33.3
CaTMS599	(ATA)5	(ATA)6	TCCATTTCCTTCCTTTTCCTC	GGTAGAAGGTGCTTGACCGA	60.0	219	4	10.572	Used	0.4	2	NA	Class II	50.0
CaGMS1263	(AG)10	(AG)7	AATCAGAAGCGGAGTGGTTG	AATGGAGGCGGGAGAGTAAT	60.0	266	4	10.681	Used	0.44	2	NA	Class II	42.9
CaGMS1150	(CT)7(AT)7	(CT)7	ATCTCGCCAATAACCACCAC	GGTAGCTAGTTGATCAAGTTCTTTCTT	60.0	197	4	10.98	NA	NA	NA	NA	NA	38.1
CaGMS20	(TAA)14	(TAA)5	TCTGCCTCGTACTCCTCACA	GGTTGTTGCTGGCATTTTCT	60.0	247	4	11.097	Used	0.73	4	Figure 2O	Class I	38.1
CaTMS812	(AG)8	(AG)7	AGAGTGGTTGGTTAGGTTGAT	AAGAGAGAAGGTTCGCTTAAA	55.0	150	4	12.847	NA	NA	NA	NA	NA	50.0
CaTMS749	(GGAATT)5	(GGAATT)6	ACATTTCCAGCACCCATTGT	CTCCCATGATCTCCACATCC	60.0	244	4	12.925	NA	NA	NA	NA	NA	50.0
CaTMS699	(AG)10	(AG)7	GGTTTTGAGCGTGGTTTGAT	TGCAAAATGTGAAGCACAGA	59.0	146	4	15.163	Used	0.47	2	NA	Class II	50.0
CaTMS1074	(CT)15	(CT)6	ATTTCCCTGTTTCCAGGCTC	GGTTGTTGCTGGCATTTTCT	60.0	265	4	15.185	NA	NA	NA	NA	NA	53.0
CaTMS773	(AT)7	(AT)8	GGTGTGGCTTTGTTGTGTTG	TCATCGCCTTCCAATCTCTT	60.0	171	4	15.412	NA	NA	NA	NA	NA	54.0
CaTMS980	(ATAAT)14	(ATAAT)6	CCGAAACAACCAAATGACAA	CAGGAGCATGAGGCTATTGG	59.0	272	4	15.657	NA	NA	NA	NA	NA	50.0
CaTMS720	(AG)11	(AG)10	GCGAGGTTGCTTTGGACTAC	TTGTTCCCACGACAAACAGA	60.0	273	4	16.253	NA	NA	NA	NA	NA	53.2
CaGMS33	(TA)12	(TA)6	TGAGAGAGGAAAAACAGATTGAGA	CACGGATCTTGTGAGTGACC	60.0	141	4	16.864	Used	0.69	4	NA	Class I	42.9
CaGMS278	(AT)11	(AT)6	TACGGCGAAATTGAAAGGAA	AGAACCAAGTTTGTCTGCACC	61.0	240	4	17.541	NA	NA	NA	NA	NA	42.9
CaGMS332	(ATT)8	(ATT)5	AAAAGAACAAAATGGAAGCCC	TGGATGCAAAAGATTTGAGC	59.0	266	4	24.241	NA	NA	NA	NA	NA	38.1
CaTMS709	(AGA)8	(AGA)7	GACCTCGAAATTGAGAAGCG	GCATCGTCATCTTTGCTTCA	60.0	109	4	28.238	Used	0	1	NA	Class I	50.0
CaGMS1278	(AT)9	(AT)7	TCCAAAACACATTCACAAGCA	TATGGATCGGAAGACGAACC	60.0	245	4	28.854	Used	0.41	2	NA	Class II	38.1
CaTMS738	(CAA)7	(CAA)5	CGGTGATGAACCTGTTGTTG	AAGCCACTCAAGACGCTGTT	60.0	234	4	29.265	Used	0	1	NA	Class I	50.0
CaTMS747	(GAA)11	(GAA)9	TGAGGATTCAAATGTTAAGGGG	TTCAGCAAGCAATGCAAATC	60.0	255	4	30.898	NA	NA	NA	NA	NA	53.3
CaTMS569	(GCA)5	(GCA)6	GTCACGGCAAAATACACCCT	AAGCGAAGATGTCAGCATTG	59.0	278	4	31.335	NA	NA	NA	NA	NA	50.0
CaGMS1308	(TA)8	(TA)6	AACGCTCCAACGTCTTAAAAT	ATGTATGAAACAAACAAACACGA	58.0	266	4	31.408	NA	NA	NA	NA	NA	42.9
CaGMS1281	(AT)10	(AT)7	TAATGCAGTGGATGCTACGG	TTATTTTTGGGATCAACCACG	60.0	207	4	31.408	Used	0	1	NA	Class II	38.1
CaTMS626	(TAT)5	(TAT)12	GGGTGTGGATAGCAATGGTT	AGCTCAATTGCCAGGAAGAA	60.0	274	4	31.408	Used	0.4	2	NA	Class II	53.0
CaGMS29	(AGA)12	(AGA)7	GGAGCTGTGAACGGTGAAAT	CGAAACGCACCCTATTTTGT	60.0	207	4	31.409	Used	0	1	NA	Class I	42.9
CaTMS745	(GTG)7	(GTG)6	AGAATCTCTCGAAAACCGCA	ATCCCGTCCATCACCTAACA	60.0	217	4	31.419	NA	NA	NA	NA	NA	53.2
CaGMS1146	(AT)7c(T)12	(AT)7	TAGAGAGCCAGCTGCACGTA	CCCTATTTTGTTCGGCTCGT	60.0	260	4	31.444	NA	NA	NA	NA	NA	38.1
CaTMS1066	(AT)15	(AT)6	TGGTTAAGAGTTGCACTCGCT	TTGCCTAAGTAAAAGTCTCATCCA	60.0	267	4	31.565	NA	NA	NA	NA	NA	50.0
CaTMS785	(AT)7	(AT)8	AACACCCCACATAATGACCC	TCATTTGACATTCACCTCACTTTT	60.0	222	4	34.934	Used	0	1	NA	Class II	50.0
CaGMS1303	(TA)8	(TA)6	TGTGCACATGACAATTAGAGCA	TTTCCATCAAGAACACGTCG	60.0	221	4	36.028	Used	0.5	2	NA	Class II	38.1
CaTMS653	(AAC)7	(AAC)6	ACGTTAGGAATGTTGTCGGC	GGAAGGTTCCACAGTTCCAA	60.0	198	4	36.053	NA	NA	NA	NA	NA	54.0
CaGMS331	(ATT)8	(ATT)5	TCTTTGAGACTGTTTTTCCCTTT	CATGTCTCATATTCCTCTAGCTGC	58.0	115	4	40.049	NA	NA	NA	NA	NA	42.9
CaTMS856	(AT)6	(AT)8	GACCTGCTCCCTTGTCTCTG	TTTTCACCTTATACGAGCTCCA	59.0	274	4	41.982	NA	NA	NA	NA	NA	50.7
CaGMS122	(TAT)24	(TAT)11	GTGGTGAGGTTAGGTGGTGC	TGAGGGATACATGAGGGACC	60.0	243	4	42.179	NA	NA	NA	NA	NA	38.1
CaGMS37	(AT)12	(AT)6	GTTGGTGCCAACATGCCTAT	TGAACCGTGTTTCAAAACCT	61.0	163	4	44.458	NA	NA	NA	NA	NA	33.3
CaTMS726	(GAA)18	(GAA)15	ACAGAGAGGGAGGTTCGTCA	CCCTTCTTCCATGTACCACC	59.0	279	4	44.484	Used	0	1	NA	Class I	50.0
CaTMS840	(TA)12	(TA)8	CGAAGCATACCAGGTTGATG	GGGATCTTGTTTCACGCTGT	60.0	267	4	44.621	NA	NA	NA	NA	NA	50.0
CaTMS1058	(AT)16	(AT)6	CAGAGCTCTCAGTCCTTCCG	TGTAGAGAAAGGGCTGTTTAGAGAA	60.0	220	4	45.065	NA	NA	NA	NA	NA	50.0
CaTMS707	(CAC)8	(CAC)6	CGTCGTTCACATACCAAACG	CCAGAAGGAGATCCTGAACG	60.0	207	4	46.247	Used	0	1	NA	Class I	52.4
CaTMS791	(TCA)7	(TCA)6	ACCACCACTGCCTCTTGTTC	CTCCAACAACTTCCCACGTT	60.0	149	4	46.286	Used	0	1	NA	Class I	50.0
CaTMS857	(ATT)5	(ATT)8	TCCCAACCTCTTTTCCCTTT	AAACGGTGTTGACCATAGGAA	59.0	135	4	46.6	NA	NA	NA	NA	NA	50.4
CaGMS1169	(TG)7(TA)8	(TG)7	TCGGAAAATTGAGAAAGTCTTCA	GCAAATGCTCCCCTAATTGA	60.0	276	4	46.738	NA	NA	NA	NA	NA	42.9
CaTMS591	(CCT)6	(CCT)5	AAGATCGCTGCTGCAAAAGT	ATCCAATCAATGGCCGATAA	60.0	246	4	46.75	NA	NA	NA	NA	NA	50.3
CaTMS889	(TGA)6	(TGA)7	TGCAGTTGCTGAAAATCTGG	GCCTTGAGCTAAAGTGATTGG	59.0	208	4	46.908	NA	NA	NA	NA	NA	50.0
CaTMS1113	(AAT)11	(AAT)6	TTATACTTTGGCCGATGTGG	GAACTACTTTCGCGATTCAGC	59.0	212	4	46.952	NA	NA	NA	NA	NA	54.0
CaTMS765	(TTC)6	(TTC)7	GTCTTGGGAACCTTGCGTTA	GATGCGTAACTACAACCGCA	60.0	160	4	47.512	Used	0.52	2	NA	Class II	53.2
CaGMS47	(TA)13	(TA)8	CTTCAACCCACCACGCTACT	CTCACTTCCTCCGTCTCTGC	60.0	271	4	47.938	Used	0	1	NA	Class I	50.0
CaGMS1240	(AG)10	(AG)7	GTTAGGGATTTGTCGGAGCA	CAACGGTCACCTTTTTGGTT	60.0	217	4	49.065	Used	0.5	2	NA	Class II	38.1
CaTMS751	(AG)8	(AG)6	GGGCAGTGAGAGATGAGAGG	CCTCACCCTTTTACCCACAA	60.0	148	4	50.931	Used	0	1	NA	Class I	54.0
CaGMS40	(GA)11	(GA)7	TCAAAGAACCAAAATTCGAACA	CGGTTGATTGATAATTCTCGTG	60.0	189	4	51.59	Used	0.73	3	NA	Class I	38.1
CaTMS689	(CTT)9	(CTT)7	GCATCACAAGCTTCAACAGC	TTTTGGGTTGATGGGTGATT	60.0	213	4	52.169	NA	NA	NA	NA	NA	50.0

*Markers identity	Microsatellite repeat-motifs in ICC4958	Microsatellite repeat-motifs in PI489777	Forward primer sequences (5'-3')	Reverse primer sequences (5'-3')	Actual annealing temperature (0C)	Size (bp) of alleles amplified	Linkage groups (LGs)	Genetic positions (cM)	Markers used for polymorphism survey	Polymorphism information content (PIC)	Number of alleles amplified	Markers used in Figures	Markers types	GC%
CaTMS564	(ATA)5	(ATA)6	TCGTCATAGTTTCAAGCGGT	CAGGAGGTGCACTATCAGCA	60.0	149	4	52.844	Used	0	1	NA	Class II	50.0
CaTMS590	(TTG)5	(TTG)7	TCACCATCGTGTGATGGACT	TTGTTGGGTCCTCTTTTGTTTT	60.0	220	4	52.851	Used	0.5	2	NA	Class II	50.3
CaGMS1266	(TA)10	(TA)7	CCTCCTATCTTCCTCCATTGAA	GCAACAAAATCACACAACAACA	60.0	223	4	52.857	NA	NA	NA	NA	NA	33.3
CaGMS1310	(TC)13	(TC)11	CGTCGTGCAGATCTTCGATA	GCGACGTCACTGTAAACCCT	60.0	248	4	53.131	Used	0	1	NA	Class I	38.1
CaTMS574	(CAA)5	(CAA)7	CGGTGTGAATTTGGTGATGA	CAACAACAACCGAACGAGTG	60.0	253	4	53.373	Used	0.37	2	NA	Class II	50.0
CaGMS1139	(CT)7(AT)6	(CT)6	TTTCGGTTGAAATGGGACTC	CCTCTGTTGGGCTAATGCTT	60.0	275	4	55.273	NA	NA	NA	NA	NA	38.1
CaGMS30	(GA)14	(GA)7	GCAAAAGACACATTTAGGGTTT	GTAGGTGCAGGCCCTACCTT	57.0	270	4	55.483	Used	0.72	3	NA	Class I	47.6
CaGMS1241	(AT)10	(AT)7	AACCGTTCACAGTAGAGGCA	CCCTATGTCCCAAAGCTACG	58.0	147	4	57.829	Used	0	1	NA	Class II	42.9
CaTMS556	(TA)7	(TA)8	TGCATCTCGCCTTGAATATG	CCACACATGCCCCTACTCTT	60.0	214	4	63.908	Used	0	1	NA	Class II	50.0
CaTMS677	(AG)6	(AG)7	CACAAAAGGGGTAAGTGGTCA	CGCATTCAACTCATTCGTGT	60.0	232	4	64.836	Used	0	1	NA	Class II	53.3
CaTMS640	(AAT)5	(AAT)8	AATGGTGGTGGTTTGAAAGG	CCATTCGCACTTTTTGTCCT	60.0	233	4	67.296	NA	NA	NA	NA	NA	50.4
CaTMS582	(ATG)8	(ATG)10	AGGACTCCCGAACTTGTTGA	CTTCCGAAATGCATCTTGCT	60.0	180	4	68.862	NA	NA	NA	NA	NA	50.4
CaTMS952	(TTA)27	(TTA)9	GAAAAACTAAAAACACCAAACCAA	CAATGTCTTACACTTTTTATGAGGGA	59.0	167	4	70.78	NA	NA	NA	NA	NA	50.7
CaGMS1158	(AGA)6g(A)10	(AGA)6	TTCTTCCTTAGAGCACAAACCC	TCCTCTCTCTCCGTTACCCA	60.0	190	4	71.011	NA	NA	NA	NA	NA	38.1
CaTMS930	(AAT)28	(AAT)8	TGTGGACCCCGCTAGATAAA	TGCAGATTTGGTGAAATGGA	60.0	233	4	71.011	NA	NA	NA	NA	NA	53.3
CaGMS1154	(TTA)6(T)10	(TTA)6	GTAGGGTTCCAAGGGGTAGC	TAGGGTTGGGTGATGGTGTT	60.0	214	4	72.096	NA	NA	NA	NA	NA	42.9
CaTMS663	(TTC)6	(TTC)5	TCCTTGCTTCCACGTTCTCT	CGATTCAGGACGACAATCCT	60.0	195	5	5.402	NA	NA	NA	NA	NA	54.0
CaTMS762	(GA)8	(GA)9	GAATCAGAAGGTGGTTGGGA	TTCGTGCCAGAAACAGTGAG	60.0	155	5	8.111	Used	0.51	2	NA	Class II	54.0
CaTMS1067	(ATA)15	(ATA)9	GAAGAACCAAAACTGTCGATTT	AGGGGGAGATGGAGAAAGAA	57.0	206	5	9.822	NA	NA	NA	NA	NA	50.3
CaTMS790	(TC)12	(TC)10	CGTAACCGAGGAGTTTGGAA	AAGGGCGTTGAAAGAAAGAA	59.0	217	5	14.779	Used	0	1	NA	Class I	50.0
CaTMS842	(GA)7	(GA)10	GCTGGTACCACCCGAACTAA	GTGAAGTAACGGGGATCCAA	60.0	233	5	17.906	NA	NA	NA	NA	NA	50.0
CaTMS1056	(TA)16	(TA)6	TCTTCACGCCCGTATATGAA	TTGATCAATTAGTGTCTAACCGTATG	59.0	241	5	22.819	NA	NA	NA	NA	NA	50.0
CaTMS1055	(AT)16	(AT)6	TGTGTATGACCACACCAAAATG	GACGTGAACGGTTGTCTGAA	59.0	160	5	23.812	Used	0.68	3	Figure 3A	Class I	50.0
CaGMS18	(ATT)19	(ATT)9	CCTTTATAAAAAGAGGTGGGGT	GTGGGTTGCAAGGAAGATTG	58.0	201	5	29.158	Used	0.67	4	Figure 2M	Class I	38.1
CaGMS42	(AG)14	(AG)9	CGAATAAATTTGGCGGAGAA	AAGCATCCAAATTGGCAAAG	60.0	275	5	29.61	Used	0	1	NA	Class I	33.3
CaGMS1137	(TG)7(TA)6	(TG)7	TTGAGGACAGGGTGAAAACC	CACCTCCCAATGTCAAAAGG	60.0	226	5	29.692	NA	NA	NA	NA	NA	38.1
CaGMS16	(ATAAA)9	(ATAAA)5	TGCTGCCAACAAACTTAGCA	GGCACCTTTCCTTGTACTGG	61.0	202	5	29.97	Used	0.69	4	Figure 2L and 5B	Class I	33.3
CaTMS551	(ACC)10	(ACC)9	TTCTCTGAATCCAACGGTCC	TGTGTGCGTGAGAGAAGAGG	60.0	149	5	37.925	Used	0	1	NA	Class I	53.3
CaTMS766	(AAC)5	(AAC)7	CTCTTGCAACTTCCCCACTC	AAAGCAAAGGAGGGTTTGGT	60.0	150	5	42.175	Used	0.4	2	NA	Class II	53.2
CaTMS668	(TA)9	(TA)6	CCGGCTTAATGAAAGAGGAA	GACCATGGGATGGATGTTTC	60.0	209	5	47.717	Used	0	1	NA	Class II	53.7
CaTMS618	(TC)8	(TC)9	CGCATCTCAAGCTTTCTCCT	CCACCTTCATCCATTATCGG	60.0	194	5	58.17	Used	0.3	2	NA	Class II	50.0
CaTMS787	(TAT)6	(TAT)5	CACTCCCTTCCTCCCAAAAT	CGCCTTTCGAATTATTGCAT	60.0	212	5	59.417	Used	0	1	NA	Class II	50.0
CaTMS715	(AG)19	(AG)6	AAGCATCAGAAGAATCAGACAGG	CTTCCTCCTCGAGATCCTCC	60.0	220	5	63.467	Used	0.65	3	Figure 2C	Class I	50.7
CaGMS23	(AT)14	(AT)6	CACATTTGTCATTTTGTAACAGCA	TGAGTTGGGAGGGGAGACTA	60.0	259	5	65.005	Used	0.67	4	Figure 2P	Class I	42.9
CaTMS1117	(TAA)15	(TAA)10	TAAAATCCTCCTCCACACCG	GGATGGACACATACATGGGG	60.0	169	5	66.18	NA	NA	NA	NA	NA	53.2
CaTMS1006	(TTA)24	(TTA)14	CAATGTGTCAATTAGGGATACATCA	TGAATGAGAAAGAGAGAAAGTGAATG	60.0	246	5	68.039	NA	NA	NA	NA	NA	50.0
CaTMS770	(AAT)9	(AAT)6	CGGTAAGAGAAGAAGCCACG	TGCATTCAATTCAATTCCACA	60.0	280	5	68.039	Used	0	1	NA	Class I	50.0
CaTMS1065	(AAT)15	(AAT)9	AAGGGGTCTATGGACTTGGC	TTTCAAAGATTTATTGTTTCGGA	60.0	275	5	68.28	NA	NA	NA	NA	NA	50.0
CaTMS621	(AAT)5	(AAT)7	ACACCACCCACCAGTAGGAA	GGGACAAGTCAGTCCGGTAA	60.0	196	5	68.28	Used	0.42	2	NA	Class II	50.4
CaTMS649	(AGA)6	(AGA)7	TCCATCCGGACAGAGTATCC	CTGGATTGTGTTCAACGTGATT	60.0	241	5	68.281	NA	NA	NA	NA	NA	54.0
CaTMS739	(GAT)7	(GAT)5	TCAACCTTCCACCAAAATCC	ATCCTGCAGAAATCGGAATG	60.0	175	5	68.528	NA	NA	NA	NA	NA	50.0
CaTMS1060	(AAT)11	(AAT)5	TGGTTGATTACTCCATTTTAGTTTTG	GGCTTGCAACCCAATTATTTT	60.0	195	5	68.555	NA	NA	NA	NA	NA	52.4
CaGMS1253	(AT)10	(AT)7	TGACCATTTGCATTAGTTCTCAA	GCCACACCACTTTCATTTTTC	60.0	246	5	69.975	NA	NA	NA	NA	NA	42.9
CaTMS1005	(TAT)15	(TAT)5	GGGGGACTAAATCCAAAACC	AGCTATTTGTCACGCCAACC	60.0	199	5	69.975	NA	NA	NA	NA	NA	50.0
CaTMS1101	(AT)14	(AT)6	ATAGTGGACCCCACAATCCA	TGTGAGAAAATTAAGGAAGAGTTTGA	60.0	268	5	69.975	NA	NA	NA	NA	NA	53.3
CaTMS877	(CAT)5	(CAT)6	CATCAACCTCAAGCCCTGTT	AGATGAGGTTGGATAGGGGC	60.0	214	5	69.975	NA	NA	NA	NA	NA	50.2
CaTMS895	(TTA)33	(TTA)8	AAAACAACCCGCACCATAAA	TTGGAATTCAATTAATTTACACGAAC	60.0	229	5	69.975	NA	NA	NA	NA	NA	52.4
CaTMS639	(CAA)5	(CAA)7	TTTCAACAATGCCAACCAAA	TCGAAGAAGGGGAAGAAACA	60.0	211	5	69.975	Used	0.28	2	NA	Class II	50.3
CaGMS1168	(GTTT)5(T)13*	(GTTT)5	TGGTCCCATTTTGTGGTTTT	TGCATCAATGCATTAAACAAA	60.0	265	5	70.002	NA	NA	NA	NA	NA	38.1
CaGMS1324	(TC)8	(TC)6	TCCAATGAGGTTGTGACGAA	CGGAGATGAATTTTCCGAGA	60.0	201	5	70.002	Used	0.43	2	NA	Class II	42.9
CaTMS567	(TGT)7	(TGT)6	GGATTGAATTGCTTGGTCGT	TCTTTTGGCACGACAAACTG	60.0	259	5	70.059	NA	NA	NA	NA	NA	50.0
CaTMS924	(AAT)26	(AAT)5	TGGTTCAAGTCACAATCCCC	ATATGAGCCACACTTCCGCT	61.0	280	5	70.088	NA	NA	NA	NA	NA	53.2
CaGMS1325	(AG)10	(AG)8	GCTTTGACCCATTTTCCAGA	TGATGTGGCTGTGTTCCAAT	60.0	278	5	70.098	NA	NA	NA	NA	NA	38.1

*Markers identity	Microsatellite repeat-motifs in ICC4958	Microsatellite repeat-motifs in PI489777	Forward primer sequences (5'-3')	Reverse primer sequences (5'-3')	Actual annealing temperature (0C)	Size (bp) of alleles amplified	Linkage groups (LGs)	Genetic positions (cM)	Markers used for polymorphism survey	Polymorphism information content (PIC)	Number of alleles amplified	Markers used in Figures	Markers types	GC%
CaTMS642	(CAA)6	(CAA)8	ACCATTTGTTTGGGCATTTC	CGAATTGAGGGTTCTTCCAA	60.0	274	5	70.107	Used	0.45	2	NA	Class II	53.7
CaTMS827	(ACA)8	(ACA)5	TTGATGAGGAAGATGGAGGG	TTGATGAGGAAGATGGAGGG	60.0	211	5	70.109	NA	NA	NA	NA	NA	54.0
CaGMS1276	(TTA)7	(TTA)5	CCTCTAAATCTTGGGTGGCA	GACTTTTCTGTCCAATGGCAA	60.0	233	5	70.122	NA	NA	NA	NA	NA	38.1
CaGMS21	(AT)15	(AT)6	CCACACAGATTCACACCACC	TGTTAATTCCTTTGCGACCC	60.0	179	5	70.122	NA	NA	NA	NA	NA	42.9
CaTMS1013	(ATA)20	(ATA)12	GAAGGTACGGCTAAAGAGTGATG	TTGTATGTGTAACACCCAATTTCTT	59.0	280	5	70.122	NA	NA	NA	NA	NA	50.0
CaTMS1020	(AT)18	(AT)6	ACATTATCATGAAAGTGAAAGAAGAA	TTTTCTTATTGTGCCGATTTGA	58.0	277	5	70.122	NA	NA	NA	NA	NA	50.0
CaTMS1061	(ATT)11	(ATT)5	CATCGGAACTCATCTCGCTA	TTGAAGTTCTTTGGTCGTTTGA	59.0	201	5	70.122	NA	NA	NA	NA	NA	50.0
CaTMS1098	(AAAT)9	(AAAT)5	TCCGAATAAATCGCATACCC	TTTAACATAAATTTTGACACTAACACC	60.0	248	5	70.122	NA	NA	NA	NA	NA	53.2
CaTMS545	(AT)8	(AT)6	AAAGAGCTTGGCAAAGCAAA	AGGGCTTCTAACAGATGGCA	60.0	266	5	70.122	NA	NA	NA	NA	NA	54.0
CaTMS701	(TCA)6	(TCA)5	ACCGTCTTCGTCATCCCTTA	GATTTATGGCCAAATCCCCT	60.0	240	5	70.122	NA	NA	NA	NA	NA	50.0
CaTMS807	(AAC)8	(AAC)7	CCAATTGGGTTGTTCCTCAA	TGCACATTGGAGAAGTAGCG	60.0	269	5	70.122	NA	NA	NA	NA	NA	51.5
CaTMS885	(TGG)6	(TGG)5	TCGAGGAAGATTGCAGGAGT	ATTGCTATTTTGGTCGGACG	60.0	182	5	70.122	NA	NA	NA	NA	NA	50.0
CaTMS936	(TTA)24	(TTA)5	GCTCCTCTCTCTCCCCATTT	TGATCAATAGAATGAAAAGAAACTGAA	60.0	230	5	70.122	NA	NA	NA	NA	NA	55.0
CaGMS1322	(AC)8	(AC)6	CGTGGGACGAACTGAGTCTT	AGTTTGGCGCTGAATTTGAC	60.0	230	5	70.122	Used	0	1	NA	Class II	33.3
CaTMS794	(ATG)6	(ATG)5	TACGGAGGCTATGGATGGTC	CACTCTTCCACTCCTTTGTGC	60.0	194	5	70.122	Used	0.47	2	NA	Class II	50.4
CaGMS1294	(AAT)7	(AAT)5	AGCGATTACCTTGAATGTGAGA	TCACACACATGGACCGAGTT	59.0	273	5	70.13	NA	NA	NA	NA	NA	47.4
CaTMS613	(CAT)6	(CAT)5	AACCTCTTCTTTCACACCTTGC	GGGGATATGATCCAGCCTTT	60.0	279	5	70.155	NA	NA	NA	NA	NA	52.4
CaTMS756	(GAA)8	(GAA)7	TTTGTCATTGCAAATCAGCC	AGTGGCTTTGATGAAATGGG	60.0	278	5	70.19	NA	NA	NA	NA	NA	54.0
CaGMS1136	(CT)6ca(CT)6	(CT)6	AAAATGCACTTGTGTTTTTGG	GCAACTTGATGCGAATTGAC	58.0	279	5	70.211	NA	NA	NA	NA	NA	42.9
CaTMS996	(TTA)21	(TTA)11	ACAGAGCCACCAGGATTGTT	GAAACTGGCCAGACGTTTTT	60.0	225	5	70.221	Used	0.73	3	NA	Class I	52.4
CaGMS1264	(TC)9	(TC)6	TGTATGTAGGCTTGGGGAGG	ATCCAGATGATGCCCCTACA	60.0	170	5	70.252	Used	0.48	2	NA	Class II	38.1
CaTMS768	(AAAC)6	(AAAC)7	AATTGAAATGGAGCAACCTCA	GCGAAACCCTTTACCTGAAA	59.0	274	5	70.263	NA	NA	NA	NA	NA	53.3
CaTMS963	(AAT)26	(AAT)8	TCATTTTTGGTTCAAATTTTAAGTCT	TGGAGATGACTTGCTTGCAG	59.0	235	5	70.264	NA	NA	NA	NA	NA	55.0
CaTMS1054	(ATTT)10	(ATTT)5	ATACATACGCGTTTGGAGGC	GGGAAGTGCTCGTAAATCCA	60.0	257	5	70.519	NA	NA	NA	NA	NA	50.0
CaTMS634	(ATA)5	(ATA)6	ATATGGTGGTGGCTGATGGT	CGTGGCAAAGACTGTTTGAA	60.0	223	5	70.71	NA	NA	NA	NA	NA	53.3
CaGMS1161	(TC)6ttc(T)14	(TC)6	TCTCAAATCCATGACGATGA	TTTTTATTTTGATTTCTTCACTACACA	58.0	280	5	70.714	NA	NA	NA	NA	NA	38.1
CaTMS515	(AAT)5	(AAT)6	AATCGCGCAAACATTTACAA	TATCCGAAGTCGATGCCTTT	60.0	273	5	70.724	NA	NA	NA	NA	NA	50.4
CaTMS650	(AT)7	(AT)6	TTTTCAAAGAACCCCAACAGA	AGCAGGACATTTGTGCAGTG	60.0	159	5	70.724	NA	NA	NA	NA	NA	54.0
CaTMS1086	(TA)14	(TA)6	GGCACGGGGGATTATTTAGT	GTGACATGGAAACATGGTGG	60.0	236	5	70.734	NA	NA	NA	NA	NA	50.3
CaTMS1036	(AT)13	(AT)6	ACCAGTTCGGTTCAAGATGG	AGCAAACTCAACTGATAAAACGA	60.0	269	5	70.909	NA	NA	NA	NA	NA	50.2
CaTMS792	(AGA)6	(AGA)5	AATGAAATGTTGCTCAGCCC	GCGATGAACAATTCAAGGGT	60.0	227	5	70.995	NA	NA	NA	NA	NA	50.0
CaTMS1082	(AT)14	(AT)6	TTGGTAGTGACTCTCAAGACCATT	TCAAATCTTCTATCAAGTGGGACA	59.0	199	5	71	NA	NA	NA	NA	NA	50.0
CaTMS554	(CT)6	(CT)7	GGATTCTTGCCCAGATTCAA	TGAAGTCAGATCAGATGGCG	60.0	203	5	71.982	NA	NA	NA	NA	NA	50.0
CaTMS706	(TG)10	(TG)7	TTTTTCCCTTTATCGCATGG	TAGGGGAAGGCAAATGTACG	60.0	145	5	73.714	Used	0	1	NA	Class II	50.0
CaTMS636	(CT)10	(CT)7	CCTCAAGTGCAACAAAAACAA	TGCAAACATTTTCACACCAGA	60.0	124	5	74.757	Used	0.45	2	NA	Class II	50.0
CaTMS736	(TAC)7	(TAC)5	TGGGCATCTCAGAAAATGCT	ATTTGGGCCTGAGAAGGTTT	60.0	261	5	75.391	NA	NA	NA	NA	NA	53.7
CaTMS546	(TCA)5	(TCA)6	CTGCAAAGAGGACACAAGCA	GACCGTGATTCTCCGTGATT	60.0	280	5	75.401	NA	NA	NA	NA	NA	53.2
CaTMS991	(ATA)24	(ATA)13	TGTGATCCATGTTACTTCACTTGTT	TCAATCTTCGTAGTCTTTTCACAA	60.0	188	5	75.831	NA	NA	NA	NA	NA	50.0
CaTMS641	(TGA)6	(TGA)5	TCCAATTCCTCACAACCACA	CTCTTAACTCCGCCGTCAAC	60.0	197	5	76.825	Used	0	1	NA	Class II	50.7
CaTMS1105	(ATA)11	(ATA)6	ACCGGGTTGGATACAAAAGA	CACTCATGGCTGAAGGAGAA	59.0	215	5	76.894	NA	NA	NA	NA	NA	54.0
CaTMS740	(AT)6	(AT)7	CACAGCAACAGAACTGGCAT	TTCATGGCACAAGAAGTTGC	60.0	267	5	77.163	Used	0.4	2	NA	Class II	50.0
CaTMS657	(TA)8	(TA)7	CGCATTCCCTTCACCTCTAA	TGAAATTCGGAAGAACCAGG	60.0	111	5	78.407	NA	NA	NA	NA	NA	54.0
CaTMS1087	(AT)15	(AT)7	TCCTCATGCCTCTGTGACTG	ACAAGGATTCGACCCACAAC	60.0	245	5	78.451	NA	NA	NA	NA	NA	50.4
CaTMS708	(ATT)15	(ATT)9	AATTAGGGTTGGATGGAGGG	CTTCCGAACCACTACGCTTC	60.0	175	5	78.497	Used	0	1	NA	Class I	52.4
CaTMS619	(TA)9	(TA)8	CCACCTCTTCCCATCACTCA	TCATGGAGAATCCCAGATCC	60.0	271	5	81.748	Used	0	1	NA	Class II	50.0
CaTMS577	(GAA)6	(GAA)8	TAATGAAAATTGTGGGAGAAG	TGACCCTCTTGTCATACTCAT	54.0	153	5	81.795	Used	0.6	3	Figure 2F	Class II	50.0
CaTMS958	(AAT)23	(AAT)5	CTCAATAGGTGGTGGGGCTA	TCATAAATTTTCACGTGTCTCATAA	60.0	275	5	81.802	NA	NA	NA	NA	NA	55.0
CaTMS700	(TCT)6	(TCT)5	CATCAGTAACCGCTCAACGA	TTTGGAGGTGGAAAGAGTGG	60.0	244	5	82.223	Used	0	1	NA	Class II	50.0
CaTMS966	(ATA)21	(ATA)5	TTCATTCTTGGTCCCAGAGG	TTCGATTAACACTTATTGTTTTGGAC	60.0	244	5	82.271	NA	NA	NA	NA	NA	55.0
CaTMS655	(TC)6	(TC)10	TATGCTGCTGCAACTCCAAC	ACACAACCTGCTCATTGCTG	60.0	218	5	83.146	NA	NA	NA	NA	NA	54.0
CaTMS806	(TTA)5	(TTA)7	GGGACAAGTCAGTCCGGTAA	ACACCACCCACCAGTAGGAA	60.0	196	5	83.146	Used	0.5	2	NA	Class II	51.5
CaTMS984	(ATA)25	(ATA)13	TCAATATCATAAGAAAATCAAGAACAG	TTGTTGTTACTGGTCAAAAACTGA	57.0	237	5	84.756	NA	NA	NA	NA	NA	50.0
CaGMS1204	(TAT)7	(TAT)5	ACGCCTTCTTTGCTCCTTTT	AGTCAAGCCTCACTCCCAGA	60.0	250	5	86.915	NA	NA	NA	NA	NA	42.9

*Markers identity	Microsatellite repeat-motifs in ICC4958	Microsatellite repeat-motifs in PI489777	Forward primer sequences (5'-3')	Reverse primer sequences (5'-3')	Actual annealing temperature (0C)	Size (bp) of alleles amplified	Linkage groups (LGs)	Genetic positions (cM)	Markers used for polymorphism survey	Polymorphism information content (PIC)	Number of alleles amplified	Markers used in Figures	Markers types	GC%
CaTMS973	(ATT)24	(ATT)10	TGTTTTCTTCGCTTTGTTTGC	AAATTGCCGTTGATGTTGGT	60.0	248	5	87.228	NA	NA	NA	NA	NA	50.2
CaTMS617	(GAA)5	(GAA)6	GAAAGATGCAATTTGTGCGA	TCATTCCATGAGAATTTTGGC	60.0	235	5	91.572	NA	NA	NA	NA	NA	50.0
CaTMS644	(TGA)6	(TGA)5	CCTATTCATCTTCCCAGCGA	TGACCATGACATGCCTGTTT	60.0	150	5	91.589	NA	NA	NA	NA	NA	50.0
CaTMS972	(TTAT)17	(TTAT)6	GTGAGGTCTAAGGCAGTGGC	GGTGAAAAAGAAAAGTGGGTGA	60.0	266	5	92.153	NA	NA	NA	NA	NA	50.3
CaTMS1012	(TAT)13	(TAT)5	TATCCGACAACGTACGAGCA	TTAATTTGGTTTGACCGATTTT	60.0	279	5	92.212	NA	NA	NA	NA	NA	50.0
CaTMS529	(CT)7	(CT)6	TGAGCTTGAACCAGTTGTCG	TCAGAACATCAAAGCCTCCC	60.0	275	5	93.056	NA	NA	NA	NA	NA	52.0
CaGMS1172	(AG)6aaagaga(GT)6	(AG)6	CGCGAAGAGAAAGAGACGAG	TGCAGGGTATGAAAGGATGA	60.0	141	5	93.659	Used	0.49	2	NA	Class II	40.0
CaTMS825	(ATT)6	(ATT)10	CCTAATTCACCACCACCACC	ATGCAACCCATTTTGGAGAA	60.0	270	5	93.766	NA	NA	NA	NA	NA	54.0
CaTMS1118	(ATA)17	(ATA)12	AAATTAATCTAACATGACATAAGGGAA	TGTGCTTGTTGAATGAGAGGA	58.0	259	5	93.891	NA	NA	NA	NA	NA	53.3
CaTMS1108	(TTA)15	(TTA)10	TTTTGGGGTTTAGGGTGTCA	AAAACTAACAACAACAAATAACGTGTC	60.0	236	5	94.84	NA	NA	NA	NA	NA	54.0
CaTMS1025	(AT)14	(AT)6	CAATTTCCTCCTATTTGTTTTGACA	CGAGGATGCGACCTTGTACT	61.0	243	5	95.932	NA	NA	NA	NA	NA	50.4
CaTMS697	(CCA)8	(CCA)11	ACATTGTTGGTCGTGTTTGC	GGGTGATGTTGGAAGGATGA	61.0	259	5	96.739	Used	0	1	NA	Class I	50.0
CaTMS680	(AAT)8	(AAT)9	CAAACGGAGAGGGATGAAGA	TGATCCATGCTTTTCCAAAT	58.0	270	5	97.385	NA	NA	NA	NA	NA	50.2
CaGMS25	(AATA)10	(AATA)6	GGATGGGGATGTCTCACTCA	CAAACATGTTCATTCCTTACTCG	61.0	276	5	97.385	Used	0	1	NA	Class I	38.1
CaTMS704	(ATC)8	(ATC)7	ACCGTTCTCTAGGACGACCA	TGAGTGAAGATGATGACGGC	60.0	216	5	98.599	Used	0	1	NA	Class I	50.0
CaGMS1132	(AT)6(AC)6	(AT)6	ATGCAAAGGTGTTTCGTGTG	CAAAGTGAGGATGAACCAATTTC	60.0	229	5	101.647	NA	NA	NA	NA	NA	42.9
CaTMS1043	(AAT)12	(AAT)5	GGGTAGAGAGATGCCTTGAGAA	TTGTAAGCTTTGTTAATATTGGATATG	60.0	208	5	101.647	NA	NA	NA	NA	NA	50.0
CaTMS922	(TAT)26	(TAT)5	CGTTCTCTCTTCCCCTTCCT	CGGTTGTAACATCCTTTTTAGCA	60.0	221	5	101.647	NA	NA	NA	NA	NA	53.0
CaTMS713	(TC)17	(TC)11	CGTTTCTCGCTCTGGAGGTA	TTTCGTTGGTTACACGGTCA	60.0	253	5	101.647	Used	0	1	NA	Class I	50.3
CaTMS598	(AAC)6	(AAC)5	TGAACAAAGGACCACACACC	TGTTCCAAGAAAAGCAGAGGA	60.0	230	5	107.015	NA	NA	NA	NA	NA	50.0
CaTMS681	(TA)8	(TA)6	ATAGCAGCAAATCCTCAGCG	GCTCATTGAAGATGACATTGC	58.0	273	5	107.685	Used	0.47	2	NA	Class II	50.0
CaTMS799	(TC)15	(TC)11	CCGATACCGGAGGATGTAGA	CCCAAACTCGACCTTGTTGT	60.0	239	5	110.192	Used	0	1	NA	Class I	50.7
CaTMS1090	(AT)14	(AT)6	CACCATTGTACACACACTGCAA	TGCAGGAAAAGCATACCAAA	61.0	279	5	111.227	NA	NA	NA	NA	NA	53.7
CaTMS686	(GGT)6	(GGT)5	TCCTCTTCGACAATTCGCTT	GAAGGAATTGGTGCTTTTGG	60.0	173	5	115.887	Used	0	1	NA	Class II	50.0
CaTMS992	(CTT)17	(CTT)6	CCGACCCAACATTTTCATTT	TGCTGGAACGAACATTCAAC	60.0	174	5	116.444	Used	0.68	3	NA	Class I	50.0
CaTMS854	(GAA)6	(GAA)7	GGTTTTTGGTTTTGGGGAAG	AACCCATTGTTGCTAATGCC	60.0	100	5	119.311	NA	NA	NA	NA	NA	50.7
CaGMS1287	(TA)9	(TA)6	TCGTTTGCACTTGTTTTAGCC	TGTGAGCACTCATCGGTCAT	60.0	201	5	119.456	NA	NA	NA	NA	NA	38.1
CaTMS679	(ATA)5	(ATA)6	GGCTATGCCAACATTCCTGT	TGAAGTAGGCCAATGCAACA	60.0	248	5	119.915	Used	0.4	2	NA	Class II	52.0
CaTMS1024	(ATA)18	(ATA)10	TTCGAATTTTCAAAGTATTCTTTTCTT	TTTGTTTAAAGGCATCATTCG	59.0	271	5	121.228	NA	NA	NA	NA	NA	50.3
CaTMS1094	(AT)14	(AT)6	GGTGACGAGGAATTGAAGGA	TGAGTCGCAGCGAGAGTAGA	60.0	225	5	121.452	NA	NA	NA	NA	NA	50.3
CaTMS921	(ATT)28	(ATT)7	TCTTTTTCTTGGTGAATAGAGTTGT	TCATTTACTGTTTACAAATTGGCAT	58.0	264	5	121.462	NA	NA	NA	NA	NA	52.8
CaTMS624	(CCA)6	(CCA)7	CGGAAACGTCACAATGTTTG	GCTACTCCTTGGTGCTACGG	60.0	250	5	121.471	NA	NA	NA	NA	NA	52.6
CaTMS774	(TCT)7	(TCT)5	TCCTAAATCTAATTCTATTTCGCATAA	ATCGCAAATGAAACGGAGTC	60.0	248	5	121.945	NA	NA	NA	NA	NA	54.0
CaTMS894	(AAT)11	(AAT)5	CAAAGCCTGGTTCTTCATCA	GGAAGTAGCCTGAGGTGCAA	60.0	266	5	132.825	NA	NA	NA	NA	NA	50.0
CaGMS478	(AT)10	(AT)6	TTGCCCACTCATTTCCTTTC	CAAATTGCATTGTTGAACCAG	60.0	265	6	19.476	NA	NA	NA	NA	NA	33.3
CaTMS588	(TAG)7	(TAG)6	TGCAAACCACTTTTCCTTCTG	CGAATCACAAAATCCATCCC	60.0	140	6	26.335	Used	0	1	NA	Class I	50.3
CaTMS702	(CTT)6	(CTT)7	CGGTTTTTCATGTGTTGGTG	CCAAGCTTTGCTGTGTTCAA	60.0	121	6	30.461	Used	0.41	2	NA	Class II	50.0
CaTMS544	(TTC)7	(TTC)6	ATAATCGACGGGAAAAGGCT	CACTCCACACTCCACCAATG	60.0	200	6	35.012	NA	NA	NA	NA	NA	54.0
CaGMS19	(AG)16	(AG)7	CAGAAAACAAACCAAAACCGA	TGCTCATGCATTTCAACCTC	60.0	191	6	36.515	Used	0.78	4	Figure 2N	Class I	33.3
CaGMS232	(AG)16	(AG)8	TCGACTGGAGTTACAGGGCT	TGAATCTGTTGATATACTTTCTTCTGC	60.0	155	6	41.921	NA	NA	NA	NA	NA	38.1
CaGMS1198	(TC)9	(TC)6	TAGGCATTTGCAACGCTATG	TTTTGCGTTCCTTTTCTCCT	60.0	202	6	44.664	Used	0.25	2	NA	Class II	38.1
CaTMS828	(AT)12	(AT)9	ACGGTGAATTCATCCCAGAC	ATCATTGCTGTGAAAGCACG	60.0	177	6	45.018	NA	NA	NA	NA	NA	53.2
CaTMS878	(AC)10	(AC)7	TTCTGACCCAGAAACAGATTCA	GCTTGCTTGCTTTTCCCTAA	60.0	166	6	46.847	NA	NA	NA	NA	NA	50.0
CaTMS727	(AT)6	(AT)8	TGCAGCAGAGCATCAAATCT	GTTGTCTGAAGGTCCCCAAA	60.0	135	6	50.071	Used	0.45	2	NA	Class II	50.0
CaGMS3	(TAT)39	(TAT)8	CACCAAAAGTGTGCACAGGA	AAGCAGTTGAAGCACCACAA	61.0	273	6	51.051	Used	0.77	4	Figure 2J	Class I	33.3
CaTMS865	(TTC)7	(TTC)6	CTGGAATCTCGCTCTCGTTC	CGCAGCTCCTCCTTTATCAC	60.0	237	6	51.648	NA	NA	NA	NA	NA	50.4
CaTMS600	(TC)8	(TC)7	CCCAGAAAAGAGAAAACGCA	ATTTCTCAACTGTCTCGCCG	60.0	269	6	52.084	Used	0	1	NA	Class II	50.0
CaTMS891	(GAA)8	(GAA)6	TCTTGGGACATAGAATTTGAA	GATGATTTCAACATGGGAATA	55.0	149	6	53.171	NA	NA	NA	NA	NA	50.0
CaTMS538	(TCT)6	(TCT)8	GGCAGCGACAACTACAACAA	TAATTGAGAATCGGGTTCGG	60.0	199	6	53.287	Used	0.48	2	NA	Class II	53.3
CaGMS15	(TTA)17	(TTA)7	AAACGCCTTGTCACCTTTTG	AATGGGGTGTTGATTTTGGA	60.0	233	6	54.345	Used	0	1	NA	Class I	33.3
CaTMS883	(GTT)7	(GTT)6	CAGGTTGAGCCACAACAAGA	TGTTTCTGTTTCTGCGTTGC	60.0	182	6	56.524	NA	NA	NA	NA	NA	50.0
CaTMS945	(ATA)24	(ATA)6	GGGACCAAAATTGCAGATGTA	TCTGACATGTGGCATCTCATT	60.0	212	6	57.477	NA	NA	NA	NA	NA	50.0
CaGMS1247	(TTA)7	(TTA)5	CTCCTCAACCGCTGCATTAT	TGGTGGTTCTTCTTCCTACGTT	60.0	259	6	58.109	Used	0	1	NA	Class I	33.3

*Markers identity	Microsatellite repeat-motifs in ICC4958	Microsatellite repeat-motifs in PI489777	Forward primer sequences (5'-3')	Reverse primer sequences (5'-3')	Actual annealing temperature (0C)	Size (bp) of alleles amplified	Linkage groups (LGs)	Genetic positions (cM)	Markers used for polymorphism survey	Polymorphism information content (PIC)	Number of alleles amplified	Markers used in Figures	Markers types	GC%
CaTMS561	(TTC)10	(TTC)13	CTGCATCAACCACACCAATC	CAACGGATAATGCACTCCCT	60.0	140	6	58.53	Used	0.75	2	Figure 2E and 4B	Class I	50.0
CaTMS893	(TC)7	(TC)9	GATCGGAATCCAAACGGTTA	CGCGATCAATTAGAGAAGCC	60.0	157	6	61.48	NA	NA	NA	NA	NA	50.0
CaGMS38	(AG)13	(AG)7	CACAATCATCATCGAGCACC	ACACACTCCTCACCGCTTCT	60.0	222	6	61.993	Used	0	1	NA	Class I	33.3
CaTMS536	(AC)6	(AC)8	GCCACCGTTGTGTTCCTTAT	CGTTTTGAAAAATCGGTGGT	60.0	183	6	62.056	Used	0	1	NA	Class II	50.0
CaTMS851	(ATC)10	(ATC)8	TGAGCGATGTTACGTGTTGAG	TGGTGGACATGAGAGGATGA	60.0	278	6	62.534	NA	NA	NA	NA	NA	50.0
CaGMS1153	(TC)6(TA)8	(TC)6	TCAATCTCCATCTTCCCCTG	CAACAAGGTCCCCACCAATA	60.0	222	6	65.854	Used	0	1	NA	Class II	33.3
CaGMS1238	(AT)9	(AT)6	CCCTGCACTACACGAGGTCT	AAATACGTGTCTGCACGTCG	60.0	277	6	66.606	Used	0.4	2	NA	Class II	42.9
CaTMS844	(CT)9	(CT)8	CTCAACGGGAGGATTGTGAT	TCGCTTCGCTTTCTTCATTT	60.0	256	6	75.671	NA	NA	NA	NA	NA	50.0
CaGMS321	(AAT)8	(AAT)5	GCACATAGCAGTTACATATGGCAG	TACTTTTGCACCTCGGTTCC	61.0	265	6	88.098	NA	NA	NA	NA	NA	42.9
CaGMS1210	(AAT)7	(AAT)5	CCCCCACAAACTCAAAGTAAG	GCTGCATTTTCACCGTACAA	59.0	265	6	93.981	Used	0	1	NA	Class I	38.1
CaGMS2	(AAT)45	(AAT)5	GTCATTTTCAACTGACTCATATTCAT	TTCTATGGAAACCCAGTGAGC	58.0	247	6	99.772	Used	0	1	NA	Class I	33.3
CaTMS888	(AG)19	(AG)8	TGGTTACAAATGTACAATGCCA	GGCAGATTCCTCACCCTACA	60.0	263	7	13.714	NA	NA	NA	NA	NA	50.0
CaTMS767	(TAT)7	(TAT)5	AAACTTGAGGGCAAGCCTTT	CAAAACACAGAGTTGAAGGAACA	59.0	103	7	14.617	Used	0	1	NA	Class I	53.3
CaGMS44	(AG)12	(AG)7	AAGCAGAGACCCATGTGGAG	AGTGGGGAGGGAAACAAACT	60.0	233	7	29.001	Used	0	1	NA	Class I	42.9
CaTMS816	(TAT)7	(TAT)6	TTGTTTGCAGGTGAAGCAAC	TCAAGTTGAAAATGGGAAATAAA	58.0	212	7	30.035	NA	NA	NA	NA	NA	53.3
CaGMS8	(ATT)28	(ATT)12	TGAACAACTCAAATAAACAATCTTCC	TCTCACTAATCCAAAACACCAAAA	60.0	257	7	30.09	Used	0	1	NA	Class I	42.9
CaTMS880	(AAT)7	(AAT)5	TGTATTTGGGGTGTGGATGA	CCCTCATGTCATTTCCATCA	59.0	185	7	47.652	NA	NA	NA	NA	NA	50.0
CaGMS1145	(AT)7ag(T)11	(AT)7	TTCGTTTTTCATTTTTCTTCAGC	ACAACGTCGCACTAAAACGA	60.0	172	7	47.771	NA	NA	NA	NA	NA	38.1
CaGMS1239	(AT)11	(AT)8	CCACATCCACAAACACAACA	TGTTCACCCAAACCAACTCA	59.0	267	7	48.044	Used	0	1	NA	Class I	42.9
CaGMS310	(ATT)8	(ATT)5	AAAAACCCGTGTCGTTTATTT	AGGGCAATATGCGATTCATT	58.0	256	7	50.318	NA	NA	NA	NA	NA	38.1
CaTMS705	(TC)17	(TC)12	TCACTCCCTCGATCCTCAAC	AGAAACTTGGCAAAAGCAGC	60.0	280	7	56.542	Used	0.72	3	NA	Class I	52.4
CaTMS953	(ATT)27	(ATT)9	GCATTGGAGATGCTTTAAGAAAA	CTGCTGCCTTATTGTGAACTT	60.0	247	7	59.046	NA	NA	NA	NA	NA	52.6
CaTMS910	(TTA)27	(TTA)5	GCTTGGACTAAATACAACCAACG	TCAACCAATCTTTTTACCCACA	60.0	210	7	59.818	NA	NA	NA	NA	NA	50.0
CaTMS841	(CT)8	(CT)6	ATTCGGTCGTATCGTCTTGG	GCGTGTGAGCGTGTAAAAGA	60.0	173	7	76.333	NA	NA	NA	NA	NA	50.0
CaTMS539	(CTT)9	(CTT)7	ATCACAACGTGGTGGTAGCC	TCTTCTTCTCCTGGGGATCA	60.0	265	7	77.976	Used	0	1	NA	Class I	50.0
CaTMS537	(GAA)6	(GAA)5	TTGAGCTGGAAGAAGGTCGT	CACTCCTCACATCCAACCCT	60.0	118	7	78.002	Used	0.47	2	NA	Class II	53.3
CaTMS1119	(ATA)19	(ATA)14	TTTATGTGTTGGCCGATTTTT	CTACCACCACCACATTGACG	59.0	248	7	84.67	NA	NA	NA	NA	NA	53.3
CaTMS606	(GTT)7	(GTT)9	GGCCACGAAAAACATCAAGT	TTGGAAATGGGTTCTCAACA	59.0	154	7	84.67	Used	0	1	NA	Class I	50.0
CaGMS190	(AAT)13	(AAT)6	CACCGGGAGATACGTAGGAG	TAGCACCCTACGACATCCGT	60.0	256	7	87.374	NA	NA	NA	NA	NA	38.1
CaTMS1051	(TTA)13	(TTA)6	GCAGTCTGAGGCTGATTTGA	CACCAACCCATGTGATTTGA	59.0	275	7	88.565	NA	NA	NA	NA	NA	50.0
CaTMS664	(TTC)7	(TTC)6	AATGGGGTCATTGGATGAAA	CTTAGAACCACCCGAAGCTG	60.0	266	7	89.11	NA	NA	NA	NA	NA	54.0
CaTMS671	(CAA)5	(CAA)7	GACGCTTTCCAGAAGATTGC	CCACTGGAATGGAGGAAAAA	60.0	242	7	89.114	NA	NA	NA	NA	NA	54.0
CaTMS1121	(AAT)10	(AAT)5	ACCCACCAAACCACATGACT	AAGAGCGGTGGCTTTCAATA	60.0	230	7	89.145	NA	NA	NA	NA	NA	50.0
CaGMS369	(AAT)10	(AAT)5	CGCACAGGGAGATACGTAGG	AGGGAAGGTATCAGCACCCT	61.0	265	7	93.121	NA	NA	NA	NA	NA	38.1
CaTMS698	(AGA)6	(AGA)5	TTTGCAAGTCTCGGTTTGTG	ACAACCGTTTCACCTTCCAG	60.0	232	7	104.212	NA	NA	NA	NA	NA	50.0
CaGMS48	(AT)12	(AT)7	TGATATGCACTCCCATTGTTATG	TCGTTGTTAAATATGACATCATTAGAG	60.0	236	7	110.531	Used	0	1	NA	Class I	33.3
CaTMS572	(GAG)9	(GAG)8	CCCACGTCATGATGAGTCAC	AGCCTCACAACTTACCCCCT	60.0	122	7	110.775	NA	NA	NA	NA	NA	50.0
CaTMS983	(TTA)19	(TTA)7	TTTAGAAATGTTTTGTCTCATTCG	TCTCTAATCCCCGACTTGGA	58.0	207	7	111.073	NA	NA	NA	NA	NA	50.0
CaTMS584	(TCA)9	(TCA)6	CGCAACTTCTCCCTTTCATC	TCATGGATTTCCTTTGCCTC	60.0	248	7	112.272	Used	0	1	NA	Class I	50.4
CaTMS631	(AAT)6	(AAT)7	TCCACTTCACCACATCATCA	TGCATATTTTGACCCTGCAT	59.0	209	7	112.813	Used	0.45	2	NA	Class II	53.2
CaGMS1248	(ATT)7	(ATT)5	TGGTCAGATTATATGGTCACGC	GCAACTTTACATGTCCCTCAAA	60.0	208	7	112.817	NA	NA	NA	NA	NA	42.9
CaTMS714	(TA)7	(TA)8	CCAAATTGAACCGAACCCTA	ACAAAGACAGGGGAAGAGGC	61.0	276	7	112.846	NA	NA	NA	NA	NA	50.4
CaTMS976	(ATT)24	(ATT)10	TGCACAACTCTAGTGTCATCCC	AGCAGTGTGTGCCTTTTCAA	60.0	208	7	113.013	NA	NA	NA	NA	NA	50.0
CaTMS550	(CAA)5	(CAA)6	CCTTCACCTATCCCACTGGA	TGGGCCAAAGTGGATAAGAC	60.0	249	7	113.445	Used	0.41	2	NA	Class II	53.3
CaTMS985	(TAA)22	(TAA)10	TAACACTGGTGCTTGTTGCC	CGTGCTCGTTATCAATCTCTTTC	60.0	257	7	116.003	NA	NA	NA	NA	NA	50.0
CaTMS744	(ATT)7	(ATT)9	CATTCCCATATTTTCCTCCG	AAGACAATCGAATCCAACGG	60.0	266	7	116.181	Used	0	1	NA	Class I	53.9
CaTMS608	(TCA)7	(TCA)6	GCGTTTTGGTTTTGGAAAGA	AACTTGCAGCCAACACTTCC	60.0	240	7	116.76	NA	NA	NA	NA	NA	50.0
CaGMS286	(AT)12	(AT)7	CGCCAACCACCACATCTAAT	TGCATTTGCCACTACTTTAATATC	61.0	261	7	131.634	NA	NA	NA	NA	NA	45.0
CaTMS1031	(TAT)15	(TAT)8	CAATGATAAATGTATCGAATTTATGGA	TGACAATGCAATACTAATATGCAAAA	59.0	258	7	136.598	NA	NA	NA	NA	NA	51.8
CaTMS1076	(TA)14	(TA)6	AATTTGCAGCTATTTTGGAGTC	CTAGCCTGGCAAGCCTATCA	58.0	254	7	138.8	NA	NA	NA	NA	NA	53.2
CaTMS769	(CTT)7	(CTT)5	CTCTGGTGCAAAGTTGGAGG	AACCACAATGCTGCTCCTTT	60.0	274	7	140.19	NA	NA	NA	NA	NA	50.0
CaTMS900	(TTA)28	(TTA)5	GGTGACCAAATGAGTACCACAA	TGTTCATAACTACACCTTTTCAAACA	60.0	157	7	140.19	NA	NA	NA	NA	NA	52.4
CaTMS742	(TAT)12	(TAT)6	AGTGGTGGAAGTATCCGTGG	AAGGATGAAAAACAGAGGGTG	58.0	237	7	140.203	Used	0.71	3	NA	Class I	50.4

*Markers identity	Microsatellite repeat-motifs in ICC4958	Microsatellite repeat-motifs in PI489777	Forward primer sequences (5'-3')	Reverse primer sequences (5'-3')	Actual annealing temperature (0C)	Size (bp) of alleles amplified	Linkage groups (LGs)	Genetic positions (cM)	Markers used for polymorphism survey	Polymorphism information content (PIC)	Number of alleles amplified	Markers used in Figures	Markers types	GC%
CaGMS1293	(GA)9	(GA)6	TGAGGGAGAGAGACACATGAAA	CGGAAATTTTGTTGTCCAAG	60.0	212	7	140.644	NA	NA	NA	NA	NA	33.3
CaTMS755	(CTA)5	(CTA)8	TCCCGTGAAACTGTCACAAA	CACCTGGAGAAGCTGAAATTG	60.0	277	7	166.979	Used	0.47	2	NA	Class II	54.0
CaTMS950	(ATA)26	(ATA)8	CCAAATCCCACAAACATATCC	AAATGGTTAAATAGGTGTTTGGATG	59.0	248	7	171.217	NA	NA	NA	NA	NA	50.4
CaTMS906	(ATA)28	(ATA)5	CCCAAATCACCTAACTTGTGAA	GCTGCATTGGACATGGTACT	59.0	280	7	171.282	NA	NA	NA	NA	NA	50.0
CaTMS678	(AT)12	(AT)7	CCAACCAATGAAAGCTAGGC	TCCTATACCAATCCCCCACA	60.0	134	7	175.335	Used	0	1	NA	Class I	51.8
CaTMS923	(AAT)28	(AAT)7	TCCAGAAAGAGGTCAACAAGG	TCACAAACATCAAAATGCGG	59.0	258	8	10.303	NA	NA	NA	NA	NA	53.1
CaGMS1234	(AT)9	(AT)6	TCATAGGCAAATTCAAGGGG	CGACGGTACTTACCAGAAATTG	60.0	265	8	19.922	NA	NA	NA	NA	NA	38.1
CaGMS1313	(TAAA)6	(TAAA)5	GCAATCACACAAACACTGCC	ATCAGCATCAGCTGCATCAC	60.0	221	8	19.922	NA	NA	NA	NA	NA	42.9
CaTMS977	(ATA)25	(ATA)11	AAATCACTCTCGCACACACG	CTCCCCTCATTCTCCCAACT	60.0	148	8	19.965	NA	NA	NA	NA	NA	50.0
CaTMS1008	(TTA)16	(TTA)6	TGCTTAAATGGCCCAGACTT	TGTCCACGTAACTTTGAAAACA	60.0	270	8	24.956	NA	NA	NA	NA	NA	51.8
CaGMS1261	(TA)11	(TA)8	ATTGGGAATTCAGCGTTTCC	ACTGAGAATTGGGGTCGTTG	61.0	280	8	24.957	Used	0	1	NA	Class I	42.9
CaTMS614	(GAA)5	(GAA)7	TGCTGGTCTTGTAGCTGGTG	GCAGCTTGTAAAGGGTTTGG	60.0	168	8	26.248	Used	0.4	2	NA	Class II	50.0
CaTMS725	(AT)8	(AT)7	TTTCGTCCCCAAATCCAATA	ATTGGTCCCCAATGTCTTGA	60.0	226	8	26.5	NA	NA	NA	NA	NA	53.3
CaGMS6	(TTA)31	(TTA)11	TGTCATGCTTATTCCGACATCT	TCTCCCACCTCTGTTGCTTT	60.0	244	8	26.515	NA	NA	NA	NA	NA	47.6
CaTMS987	(AAT)17	(AAT)6	TCAACTTGAAAAGAGCACGAGA	TTAGGCTTGATATGCCGACC	60.0	210	8	26.535	NA	NA	NA	NA	NA	50.0
CaTMS887	(GATTCA)6	(GATTCA)5	TTGCCGTCCGAATCTTCTAC	CGTTCCTGACGTTCCTTTTC	60.0	276	8	28.165	NA	NA	NA	NA	NA	50.0
CaGMS1235	(TTA)7	(TTA)5	CCCTCTTCTCTACGACACCG	CGGTGGACATCCTCGTATCT	60.0	279	8	29.098	Used	0.74	4	NA	Class I	33.3
CaTMS563	(TTG)5	(TTG)6	GGCTGGGTTGATTTATGGTG	AGACAGCAAGCAAGCAGTGA	60.0	141	8	36.289	NA	NA	NA	NA	NA	50.0
CaTMS741	(AG)9	(AG)12	TTCCAGATCTCCGGTAGGTG	ACTCTCCACTCTCCCAACCA	60.0	209	8	36.868	Used	0.44	2	NA	Class II	50.3
CaTMS603	(TAT)6	(TAT)5	CAAAAGGGCGTTAAGGTTCA	GGAACCCTATGGTTTCCGA	60.0	271	8	53.722	NA	NA	NA	NA	NA	50.0
CaTMS609	(TCT)6	(TCT)9	TCAACCCTGTTTTGGTTCCT	TTGTTCCTTATTGTCACAACACC	59.0	217	8	53.722	NA	NA	NA	NA	NA	50.0
CaTMS818	(TCAC)7	(TCAC)6	AGGGTTTGTGAAATTGCGAA	GACTGAGAAGGAGCGGTGAC	60.0	118	8	54.348	NA	NA	NA	NA	NA	50.0
CaGMS17	(AAAT)12	(AAAT)7	CCGAATTCCCTGTTGTAGGA	ACTGCATGTTTGCCAGGTTT	60.0	276	8	54.348	Used	0	1	NA	Class I	42.9
CaTMS760	(ATG)8	(ATG)7	TGGTGAAGAAACAGGAACCA	ACCTGGAGAAGAAGAGAGGG	57.0	259	8	54.363	NA	NA	NA	NA	NA	54.0
CaTMS974	(AAT)27	(AAT)13	GGTCCCAAAGAAACAAGCAA	TGAATAATTTTGAAGATGGTGTTTG	60.0	271	8	54.731	NA	NA	NA	NA	NA	50.2
CaGMS485	(AT)10	(AT)6	CTCCAACCAATTATGGACGC	CATTTGCAAGGTTATGGCAC	60.0	208	8	66.342	NA	NA	NA	NA	NA	30.4
CaGMS11	(AT)21	(AT)6	TCGGATTAGTGCATGAGAAAA	GGATAATACGAGTTTGTTCGCAA	58.0	205	8	70.906	NA	NA	NA	NA	NA	38.1
CaGMS1156	(AC)6AGAG(AT)11	(AC)6AGAG(AT)6	GAAGAACTCGCGAGAAGGAG	TGAGTCGCGACGAGAGTAGA	59.0	140	8	71.81	NA	NA	NA	Figure 3B	NA	42.9
CaTMS612	(GAA)5	(GAA)8	TAGTTGCTGCCTTCGGAACT	GGCGGATACTACTTTGTCGC	60.0	200	8	72.509	Used	0.45	2	NA	Class II	50.0
CaTMS1052	(AT)16	(AT)6	TTTTCCAATAGAACTTCAAATCAA	TTTCATCGTGATCTTGTAAGGC	57.0	242	8	72.612	NA	NA	NA	NA	NA	50.0
CaGMS1269	(TTA)7	(TTA)5	AAGATTTACCGCATCACATATCTC	CCTCCTACCCCACATTACCA	59.0	203	8	72.714	NA	NA	NA	NA	NA	33.3
CaTMS1088	(AT)14	(AT)6	TGTGCAACTGATGTCGAAGA	GGCTACGATTTTGTGCTTCA	59.0	251	8	72.714	NA	NA	NA	NA	NA	50.7
CaTMS604	(TCC)7	(TCC)5	AAACACAGATGTCGTCGCAA	CACTGATATTCGGCAACACG	60.0	156	8	72.788	Used	0.73	3	NA	Class I	50.0
CaGMS1173	(CAA)9	(CAA)7	TCAACTGCAGAAGGAGATGG	CCCTTGATTGTCCTTGTGCT	59.0	196	8	72.875	NA	NA	NA	NA	NA	38.1
CaTMS1011	(ATT)15	(ATT)5	CAAGAACTTTTAAACAATCATTTTTGG	CGGAGGGAATGCGTTATACT	60.0	218	8	72.875	NA	NA	NA	NA	NA	50.2
CaTMS1044	(AAT)12	(AAT)5	CGACTGTAATACCTCAATTTTGTTTG	AAGATGGGTTGGCTGACAAT	60.0	280	8	72.875	NA	NA	NA	NA	NA	50.0
CaTMS853	(TGA)7	(TGA)6	CCAAGAATCTTTGCTTTGGA	GAAAGGCGTTACCTTCCCTT	60.0	254	8	72.875	NA	NA	NA	NA	NA	50.0
CaTMS674	(GGA)6	(GGA)7	AGAGAAGAGAGAGGGGTCGG	CGACTTCGAGTTCCGTCTTC	60.0	184	8	72.875	Used	0	1	NA	Class II	53.3
CaGMS1144	(TAT)6(T)10*	(TAT)6	AACGGTGTTGGAGAATGCTT	TGTTTAGTCTGGTGCCATTTTG	60.0	244	8	72.88	NA	NA	NA	NA	NA	42.9
CaTMS1050	(TTA)15	(TTA)8	ATCAGAAGTGGGGAACGTGA	GGAACATCCAGGCATTTCAT	61.0	277	8	72.906	NA	NA	NA	NA	NA	50.0
CaTMS717	(CT)17	(CT)8	CCTCTTCAAACCATCCTCACA	TCTCGAGCGATCCATCTTTT	60.0	201	8	72.997	NA	NA	NA	NA	NA	52.8
CaTMS520	(TAT)7	(TAT)6	CTGGCACAAAGGGTGAGATT	CAAACCAACCCATGAAGACA	59.0	168	8	73.23	NA	NA	NA	NA	NA	51.2
CaGMS1237	(AT)9	(AT)6	AGGCCAGTTGACACCATAGC	GCTGGAACCATTTCCAATGT	60.0	171	8	73.249	Used	0.45	2	NA	Class II	38.1
CaTMS782	(CTT)7	(CTT)6	CCAAAACCATTTTCCTTCAATC	TATGGATCGGAAGACGAACC	60.0	191	8	74.469	Used	0	1	NA	Class I	50.0
CaTMS735	(AAG)6	(AAG)5	TTTCCTCACTTCAAAGACCCA	ATTTGACGAAACTCCAACGG	60.0	252	8	74.731	Used	0	1	NA	Class II	50.7
CaTMS1048	(ATA)19	(ATA)12	ATCCGTGTCGTTTCAACTCC	TCACTGAGGAGCTGATCCCT	60.0	187	8	75.27	NA	NA	NA	NA	NA	50.0
CaTMS843	(AT)14	(AT)13	TACACTTAGCATGGCACCCA	ATAACCTCGAAAACAGCCGA	60.0	148	8	76.985	NA	NA	NA	NA	NA	50.0
CaTMS1042	(AAT)15	(AAT)8	TTTCCTCTTGTATGTAAACAACCTT	GATGAAATTGTAAATATTACCACACAA	58.0	209	8	78.691	NA	NA	NA	NA	NA	50.0
CaGMS1	(ATAA)35	(ATAA)5	AAAGCTAAGGACCCCACGTT	TATGCTCCAAGGGATGCAAG	60.0	280	8	79.501	Used	0.71	4	Figure 2I	Class I	33.3
CaTMS632	(ATG)6	(ATG)8	TCAAAGGGAAAGGATTTTGG	AGGGTCCCTCAGTATTGCCT	60.0	145	8	80.977	Used	0	1	NA	Class II	53.2
CaTMS1049	(ATA)12	(ATA)5	CTTCGATGGTAGCAGAAGCC	TTTCTCTCTCGGACTTTCGG	60.0	279	8	82.055	NA	NA	NA	NA	NA	50.0
CaTMS524	**(CT)7**	**(CT)6**	**AAATCCCAAACCGAAACTCA**	**AGCATCGCAAAAATCAGAGG**	**60.0**	**205**	8	82.105	NA	NA	NA	NA	NA	51.5
CaTMS978	(TTA)22	(TTA)8	AAAATGTATTGGACTTGAGAAATTCA	TGCACATAGAGAACTGAGAATAAAAA	59.0	250	8	82.114	NA	NA	NA	NA	NA	50.0

*Markers identity	Microsatellite repeat-motifs in ICC4958	Microsatellite repeat-motifs in PI489777	Forward primer sequences (5'-3')	Reverse primer sequences (5'-3')	Actual annealing temperature (0C)	Size (bp) of alleles amplified	Linkage groups (LGs)	Genetic positions (cM)	Markers used for polymorphism survey	Polymorphism information content (PIC)	Number of alleles amplified	Markers used in Figures	Markers types	GC%
CaTMS672	(AGA)7	(AGA)8	CCGAAAGAAAACGATTCCAA	GAATTGCCTAGCTGCGACTC	60.0	131	8	82.12	NA	NA	NA	NA	NA	54.0
CaTMS1116	(AAT)15	(AAT)10	CCTTGACGATATGAGATCCGA	CCTTGAAACCTTGCAGTTCC	60.0	260	8	83.495	NA	NA	NA	NA	NA	53.2
CaTMS666	(TTC)10	(TTC)5	TCTTTCGCTTTCACTCCCTC	GGTGGAAGCGGTTATTGAGA	60.0	227	8	84.22	Used	0	1	NA	Class I	53.9
CaTMS1037	(AAT)12	(AAT)5	GGCTTATCCATACCCCGTTT	TGCAACGAAACAAATTGGAG	60.0	231	8	109.57	NA	NA	NA	NA	NA	50.0
CaTMS1027	(AT)17	(AT)6	TCACGTGCATGCAAGATAAAT	AGGGAAAGGAGCAAAATTGA	59.0	252	8	109.788	NA	NA	NA	NA	NA	50.7
CaTMS1010	(TTA)15	(TTA)5	AACAATCTTAGAAGGTTTAAACAAAAA	TTGTTATTTGTGCATTATGTTTATGG	57.0	243	8	109.925	NA	NA	NA	NA	NA	52.0
CaGMS1170	(AC)7(AT)8	(AC)7	TTGTCTGTCCGCCTTATTCC	CAATGTTGAAGACGCCAAAG	60.0	129	8	109.962	NA	NA	NA	NA	NA	38.1
CaTMS637	(TC)10	(TC)9	AGCGATGAGAGCGAGTAAGC	TCTCTCCCCAATTCACCATC	60.0	278	8	109.962	Used	0.44	2	NA	Class II	50.0
CaTMS722	(GAA)9	(GAA)6	GACTAAGGCCTCAAAACCCC	ATCACCACTTCTTGCATCCC	60.0	237	8	109.977	NA	NA	NA	NA	NA	53.2
CaTMS823	(GAT)6	(GAT)9	GTGGCCAGGTGAGAGAAGAG	ATGGTAGTTTGGCGGTGAAG	60.0	233	8	109.977	NA	NA	NA	NA	NA	54.0
CaTMS982	(TTA)20	(TTA)8	TCTCTTCGCACCTCATTGATT	GCAATGCATACATGGACCCT	60.0	222	8	109.977	NA	NA	NA	NA	NA	50.0
CaTMS615	(TTA)7	(TTA)5	AGATCCACCTCCACCTTGTG	TTGGAGGTTGTGTTGTTGGA	60.0	185	8	110.468	Used	0	1	NA	Class I	52.4
CaTMS1018	(AAT)15	(AAT)7	TTCAAATTCAATAATTATCAGCAACA	TTTTTGTAGGGCATGGAAAT	59.0	259	8	112.757	NA	NA	NA	NA	NA	50.0
CaTMS630	(AT)6	(AT)8	GGCTGTGTTTGGTTGTGTTG	TTGCATGCTTTTACCAAACC	59.0	180	8	112.765	Used	0	1	NA	Class II	53.2
CaGMS35	(AT)12	(AT)6	GAGGTGTAACATCCCGCTTT	GCCAAGAGTTTTGGATTTAGGA	59.0	221	8	113.222	NA	NA	NA	NA	NA	34.8
CaTMS1038	(AAT)18	(AAT)11	GGTCACGTCATGGATTTGTG	TTGTCTATTTTATGGGGGCG	60.0	227	8	113.222	NA	NA	NA	NA	NA	50.0
CaTMS648	(CT)16	(CT)12	GCGATCTCTCGAAAACCCTA	GAACGCAAACCACATGATTG	60.0	159	8	113.42	Used	0	1	NA	Class I	54.0
CaGMS1309	(TA)9	(TA)7	AGTGGGGGACTGTGTTGAAG	CGTTTGGTCTATCGTCGGTT	60.0	277	8	113.431	NA	NA	NA	NA	NA	38.1
CaTMS882	(TGA)14	(TGA)6	CGAGGATCTCTGGGAAATGA	AAGCGTGCTGCTTCAGAAAT	60.0	213	8	113.537	NA	NA	NA	NA	NA	50.0
CaGMS1250	(TA)10	(TA)7	GGGGATTGTCAGAAACGGTA	TTGTCGACACATCCTCTCAAA	60.0	223	8	114.632	Used	0.41	2	NA	Class II	33.3
CaTMS837	(GGT)8	(GGT)7	TGTGCCAAGTGATGTTGGTT	AGCATGCAAAAGCATGTTGA	60.0	269	8	114.648	NA	NA	NA	NA	NA	50.0
CaTMS1114	(GAT)10	(GAT)5	GCAACAAAAGAAAAGGCAGC	TTCTTCCTCCTCCTCTTCCC	60.0	195	8	114.648	Used	0	1	NA	Class I	54.0
CaTMS892	(AT)10	(AT)8	TGTGGAACTCCATACCCAAAG	TGACATGAAACCAAAGCATCA	60.0	174	8	114.652	NA	NA	NA	NA	NA	50.0
CaTMS516	(AAT)6	(AAT)5	AGTCTTGGTGGTGTTTTCGG	TCTACCCCCAGTGCATTCTT	60.0	140	8	114.668	NA	NA	NA	NA	NA	50.7
CaGMS1185	(GA)9	(GA)6	TTCACTCAAATGTAGCAATCAAA	AATTATTGCCTGAGTTGCGG	58.0	214	8	114.685	NA	NA	NA	NA	NA	38.1
CaTMS1102	(AT)14	(AT)6	TGAATGACTCAAAGGAGTGTCA	GGTGTGTGCTCCCTTTTCAT	58.0	234	8	114.685	NA	NA	NA	NA	NA	53.3
CaTMS833	(ATA)5	(ATA)11	GGTTCATTTCAAAAGCGGAA	CTTCCTTCAAACCAAGCCAA	60.0	123	8	114.685	NA	NA	NA	NA	NA	50.0
CaTMS860	(TCT)6	(TCT)7	TAACGGTAGCAGAAGTCGCA	GAAACGGTGGATGGAGAGAA	60.0	194	8	114.687	NA	NA	NA	NA	NA	50.4
CaTMS979	(TTA)23	(TTA)9	CCCAATTAAATGACGAAAAATCA	TTGGTTCAAACCTTTTCTGTTT	60.0	280	8	114.687	NA	NA	NA	NA	NA	50.0
CaGMS12	(AAT)22	(AAT)13	TGGGGTGGTGTACAAGAGAAG	TTTGACATGAATGATGTTTTGAGA	60.0	178	8	114.714	NA	NA	NA	NA	NA	42.1
CaGMS1262	(AT)9	(AT)6	CTACCTTATGGGCACGCAAT	AGCCAATTTGGTGTTGGAAG	60.0	265	8	115.144	Used	0.46	2	NA	Class II	42.9
CaTMS737	(AGA)5	(AGA)6	AAGAAATGAGGCAGTGGTGG	GCCCACTCTACTTTCACAGCTT	60.0	184	8	115.156	NA	NA	NA	NA	NA	53.7
CaTMS822	(AT)6	(AT)7	GGCTGGAATTGGCAAAACTA	AAGCTGCTTCAGGTGCTGTT	60.0	242	8	115.156	NA	NA	NA	NA	NA	50.0
CaGMS1138	(AT)6(AG)7	(AT)6	AGGCTATAAGAATGTCCACCTTT	AGAAATGCAAGCATCGTCAA	57.0	237	8	115.157	NA	NA	NA	NA	NA	38.1
CaGMS1184	(TGA)7	(TGA)5	ACGCTGATGATGATCGTGAA	TGTGATGCAACTAAGCCACC	60.0	235	8	115.157	NA	NA	NA	NA	NA	42.9
CaTMS988	(ATA)22	(ATA)11	GCTGTTTTCTCTATTTCAAGATCG	GCATTGTTCGGAATTGATGA	59.0	270	8	115.16	NA	NA	NA	NA	NA	50.0
CaTMS1099	(TA)14	(TA)6	GACTGTGGTCTGATTATTTTTCTCAA	AAAAATTCCAAATGTCCTCAAA	60.0	242	8	115.163	NA	NA	NA	NA	NA	53.2
CaGMS1155	(AC)6tctg(AC)6	(AC)6	TGTGGTTCATCCTCATCCAA	CCTTTGCAAGTTTCACCGAT	60.0	242	8	115.174	NA	NA	NA	NA	NA	47.6
CaTMS1080	(TA)14	(TA)6	TCACATTCTCACATTAAATAAACAAAA	CGCGCGCAAATACATACATA	58.0	253	8	115.174	NA	NA	NA	NA	NA	53.3
CaTMS855	(AAG)6	(AAG)5	TGCGTTGTTGTTGAGAGGAG	TGAGAACCATGGCAATAGCA	60.0	231	8	115.174	NA	NA	NA	NA	NA	50.7
CaTMS638	(ATG)7	(ATG)6	TAAACAAACCGGGAAGTTGG	CCCCTTACGAATCCCATTTT	60.0	137	8	115.174	Used	0	1	NA	Class I	50.0
CaGMS1149	(AG)8(TG)6	(AG)6	GAGTAAAAGGGTCGTTGCGA	CTCCTCTTGCATTTCAACCG	60.0	232	8	115.176	NA	NA	NA	NA	NA	38.1
CaTMS1002	(ATA)15	(ATA)5	ATCGCGCAAGAAAAGAAAAA	AAACTTCTCGAGTGTGCGGT	60.0	276	8	115.176	NA	NA	NA	NA	NA	55.0
CaTMS1029	(TAA)12	(TAA)5	CCTTCACACTTTAACTCAACCTTC	GAGGTGTAAAACTCCAATTTTCTATGA	58.0	255	8	115.176	NA	NA	NA	NA	NA	51.5
CaTMS1033	(TAA)13	(TAA)6	CCCCAATCCATTATGCTTTC	CTCGATCAGGGACAACACTG	59.0	279	8	115.176	NA	NA	NA	NA	NA	52.0
CaTMS1115	(ATT)10	(ATT)5	TCGGGTTGAAGTTAGTATTAAAAAGA	TAATGGGTCAGCAATGAGCA	59.0	217	8	115.176	NA	NA	NA	NA	NA	54.0
CaTMS597	(AAG)8	(AAG)5	GCAGTGGAGGTGAATTCGAT	GGGTGCACCTGTTTCAGAAT	60.0	125	8	115.176	NA	NA	NA	NA	NA	50.0
CaGMS1207	(TC)12	(TC)9	GATGGTGCTGTGAAATGTGC	TCCGCATTGAGATAAGGTCC	60.0	214	8	115.176	Used	0	1	NA	Class I	42.9
CaGMS1176	(AT)9	(AT)6	CAATTTCCTCCTATTTGTTTTGACA	TGATTCTCGAGGATGCGAC	61.0	250	8	115.176	Used	0.45	2	NA	Class II	33.3
CaTMS1081	(TTA)14	(TTA)6	GTTTGTTTGGATAGCGGACC	TAGTGGATAGGTCGGTTCGG	59.0	226	8	115.177	NA	NA	NA	NA	NA	53.3
CaGMS1142	(CT)6(CA)7	(CT)6	TTTACACCCCCTAATTCCCC	CAATTTCGGCCAAGAACATT	60.0	151	8	115.18	NA	NA	NA	NA	NA	42.9
CaTMS658	(CTA)7	(CTA)5	CTTGCATTGCACTTCTTCCA	TGCAGAAGGAATTGTGCTTG	60.0	203	8	115.182	NA	NA	NA	NA	NA	54.0
CaGMS1140	(AC)7(AT)6	(AC)7	CTTTCCATTTCCCATCACAA	AAGTCCTTGCAAACAAATCTCC	58.0	218	8	115.19	NA	NA	NA	NA	NA	38.1

*Markers identity	Microsatellite repeat-motifs in ICC4958	Microsatellite repeat-motifs in PI489777	Forward primer sequences (5'-3')	Reverse primer sequences (5'-3')	Actual annealing temperature (0C)	Size (bp) of alleles amplified	Linkage groups (LGs)	Genetic positions (cM)	Markers used for polymorphism survey	Polymorphism information content (PIC)	Number of alleles amplified	Markers used in Figures	Markers types	GC%
CaTMS1064	(AT)12	(AT)6	GGTCCCAATCCAAATCTCCT	AAAACTCACGAAGATTAATTGTGTTCT	60.0	242	8	115.192	NA	NA	NA	NA	NA	50.0
CaTMS898	(TAT)29	(TAT)5	GAGGGACACATTGTTTTAATAACTTTT	CATATTTGCAAGTACCAAAAACAT	59.0	279	8	115.192	NA	NA	NA	NA	NA	52.4
CaTMS534	(AGG)5	(AGG)6	CTGAGCAGCAGAGGATGTCA	ACCTTGCTGTTGGCGATAAT	60.0	209	8	115.193	NA	NA	NA	NA	NA	50.0
CaGMS1152	(TC)9(T)10	(TC)9	TTACATGAAAAATAAAAACAAGCAA	CCGGAAGATTTAGCAATGGA	57.0	199	8	115.194	NA	NA	NA	NA	NA	42.9
CaTMS1106	(TAT)14	(TAT)9	TTTTCTTGAATCTTGGATTAGCC	TGCCACTCACTTGTTTGCTT	59.0	275	8	115.194	NA	NA	NA	NA	NA	54.0
CaTMS940	(TAT)25	(TAT)6	TGACAAACATGTGTCTCTTGTATAGTT	AACAAAAAGACGCGACAAAGA	58.0	207	8	115.194	NA	NA	NA	NA	NA	55.0
CaTMS1096	(AT)14	(AT)6	CCATGCTTGTGACGTTCTCT	GCCTTTTCTTTTTCTTTGGGA	59.0	279	8	115.194	Used	0	1	NA	Class I	50.7
CaGMS1157	(GA)6a(AG)8	(GA)6a(AG)6	GTTGCTTGGCCAAAAATATG	CCATCGATTGTATTGAGGCA	58.0	238	8	115.2	NA	NA	NA	NA	NA	47.6
CaGMS1162	(TC)8(TA)7	(TC)7	CCCTTTCACCTTCACTCCAA	GCATATTAAACGGTTGCGTG	60.0	275	8	115.2	NA	NA	NA	NA	NA	42.9
CaTMS1003	(TTG)17	(TTG)7	TACGAGGTGGAGTTTGGAGG	CCTCACATACCCCTAGCGAA	60.0	195	8	115.2	NA	NA	NA	NA	NA	50.0
CaTMS1026	(ATA)13	(ATA)5	GCAAATTAATGATTTAACACAAATACA	TGAACGGTTGATGATGCTGT	58.0	260	8	115.2	NA	NA	NA	NA	NA	50.7
CaTMS1104	(AAT)17	(AAT)12	CAATAGCTCCATAATGCTAATAAAACA	CACGTTTATATCAGAATCACATTCAA	59.0	242	8	115.2	NA	NA	NA	NA	NA	50.0
CaTMS1109	(AAT)10	(AAT)5	TGCTCTGTCATATCTAATGTTCATCC	CTTCCACTGTGCCACAAAAA	61.0	239	8	115.2	NA	NA	NA	NA	NA	54.0
CaTMS696	(TGA)6	(TGA)7	TTCCATCCATTCTTTGCCTC	CTGGTGTCATACATGCAGGC	60.0	260	8	115.2	NA	NA	NA	NA	NA	50.0
CaTMS718	(CTT)6	(CTT)7	AAGCAAATCGAATAATGGCG	CAGCATTCACCTTCAAAGCA	60.0	171	8	115.2	NA	NA	NA	NA	NA	53.0
CaTMS784	(GAA)9	(GAA)6	GACTAAGGCCTCAAAACCCC	ATCACCACTTCTTGCATCCC	60.0	237	8	115.2	NA	NA	NA	NA	NA	50.0
CaTMS789	(TA)7	(TA)8	AGCCCCTTCGATGTGTAGTG	GCCAATATTTGGACGCAAGT	60.0	204	8	115.2	NA	NA	NA	NA	NA	50.0
CaTMS850	(TGG)10	(TGG)6	GGTTTTGAGAGAGAGTGCGG	TCTTCCGCAAAACAAAAACC	60.0	203	8	115.2	NA	NA	NA	NA	NA	50.0
CaGMS1208	(AT)10	(AT)7	GTAAGCCAACTTCCTTGGCA	AACCCACAACCACTTCCATC	60.0	196	8	115.2	Used	0.41	2	NA	Class II	33.3
CaGMS1219	(TA)9	(TA)6	TCACAATCCAAACCACCAAA	GTGATGGGTTGCCACGTTAT	60.0	250	8	115.213	NA	NA	NA	NA	NA	33.3
CaGMS1127	(AC)6(A)10	(AC)6	TTGTTTCTCCAAACCAACTACA	TGCTTCCATAAGTTCTCCCG	57.0	119	8	115.219	NA	NA	NA	NA	NA	33.3
CaGMS1141	(AT)6(AC)7	(AT)6	AACTTACCTTCGCTCCCGTC	CGGCTGTTCTGCCATAAGAT	61.0	168	8	115.219	NA	NA	NA	NA	NA	33.3
CaTMS1092	(AT)14	(AT)6	CAACAAAATGAATGAATTAAGATGAAA	CGATATGAACACAAACATGTGG	60.0	230	8	115.219	NA	NA	NA	NA	NA	50.0
CaTMS723	(CCG)5	(CCG)6	GATGTTCACCGGAAGTTCGT	AGGTCTTGGTGGAAGTGGTG	60.0	175	8	115.219	NA	NA	NA	NA	NA	53.2
CaTMS986	(ATA)24	(ATA)12	AATTGAGTTTGAAAGAATTGAGACA	AAGAGGGTGAATTGACTTACGG	58.0	278	8	115.219	NA	NA	NA	NA	NA	50.0
CaTMS994	(TTA)22	(TTA)12	AACGTCGGCAAGAAAGAAAA	TTTTGTCTATTGTGTTGAATTATGCTT	60.0	187	8	115.219	NA	NA	NA	NA	NA	52.4
CaGMS1177	(TAT)7	(TAT)5	CATCAACGACGACGAGAGAA	AACACTTGGCCGTTGGTTAC	60.0	214	8	115.219	Used	0	1	NA	Class I	47.6
CaTMS620	(AT)9	(AT)15	TTTTTCATCCATCACATCATCA	TTGATGCTTTACAACGTCGC	60.0	261	8	115.219	Used	0.47	2	NA	Class II	50.3
CaGMS1159	(TA)7ttgtc(T)10	(TA)6	TTTGTTCTATGTTGTCCCAACC	TGTGCATTAATGATCCATTTGTC	59.0	280	8	115.268	NA	NA	NA	NA	NA	38.1
CaTMS694	(TTG)6	(TTG)5	CTGTGGCTGTGCTGTCTGTT	ATTGTTGTGGGCTGAGGAAT	59.0	217	8	115.311	NA	NA	NA	NA	NA	50.0
CaTMS1078	(TTTA)9	(TTTA)5	TGCGACATCTTTTTAATTTTTCAG	AAAGTGAGTTTCAAAACTACCAAGTA	60.0	262	8	115.455	NA	NA	NA	NA	NA	53.2
CaTMS1071	(AT)15	(AT)6	GGTTGTGTTGTCTTATTGTCAAGC	CACTAAACCCTGGCCTAAACTC	60.0	280	8	116.318	NA	NA	NA	NA	NA	50.7
CaTMS809	(GGT)7	(GGT)5	CGTTACGATATTCGGGTGCT	GCTTCCCTTACGCTTAACCC	60.0	161	8	116.33	NA	NA	NA	NA	NA	52.0
CaTMS829	(AG)17	(AG)15	TGTGAGCGGTAGATGAAACG	TCCTCCCATTCTTTGCATTC	60.0	246	8	116.33	NA	NA	NA	NA	NA	53.2
CaTMS1023	(TAT)16	(TAT)8	TGTGAAACAATCAAATTAAGGACAA	GACCCCACCAGCTAGTGAAC	60.0	279	8	116.337	NA	NA	NA	NA	NA	50.0
CaTMS1075	(TTA)11	(TTA)5	CCTAAAGACGTAACTTGTGAACG	GTACAGGTGCACGACCTTGA	58.0	257	8	116.337	NA	NA	NA	NA	NA	53.1
CaTMS1091	(AT)14	(AT)6	TTAGCTTCAACGTATTTTTCAACA	TGGTTATCTAGTCACCCAAAAGAA	58.0	183	8	116.337	NA	NA	NA	NA	NA	50.0
CaTMS585	(AG)9	(AG)8	TTCGGGAACCTTGAATTCTG	CCAACCTCAACCTCAAAACC	59.0	215	8	116.337	NA	NA	NA	NA	NA	50.4
CaTMS593	(GGA)9	(GGA)13	GCCTCATGCATCACAAGAAA	GCATTTTGCATGTTTGAACC	59.0	264	8	116.337	NA	NA	NA	NA	NA	50.2
CaGMS1252	(GAT)7	(GAT)5	TGAGGGTACTCCTAAACCCG	CCCCCTTCTTTTCCTTCAAC	59.0	180	8	116.337	Used	0	1	NA	Class I	38.1
CaGMS1306	(TATT)6	(TATT)5	GCAGCTGAACTAATTGGCCT	ACAGGAAGAGCTCCCTAGCC	59.0	201	8	116.345	NA	NA	NA	NA	NA	38.1
CaTMS510	(TGA)7	(TGA)8	TAATTGGAGGCTTGTACCCG	GATTCCCCCAAGGAATCATC	60.0	244	8	116.345	NA	NA	NA	NA	NA	50.0
CaTMS514	(TA)7	(TA)8	ATTCCCATTAGCAAGTTGGC	CAATGGTTGCTTTCCTTGGT	60.0	270	8	116.345	NA	NA	NA	NA	NA	50.3
CaTMS647	(TAT)6	(TAT)7	GATTCACCAAACCAAGCCAT	AAAGAGTGCAGGGGATGTTG	60.0	251	8	116.347	NA	NA	NA	NA	NA	55.0
CaTMS719	(ATC)6	(ATC)5	AAGACCAATCATCAAACCGAA	AAGCTTGTGCAGGGAACACT	60.0	172	8	116.347	NA	NA	NA	NA	NA	53.1
CaTMS779	(AG)7	(AG)6	TGTCAAAAGCGAGAATTCCA	GGAAGCAGCTGGTTTTGTTG	61.0	280	8	116.347	Used	0	1	NA	Class II	53.3
CaGMS1200	(AT)8	(AT)6	TCACGGTCAATTCATTTTGG	TTGTTCTGTTTTCTACGCGG	59.0	168	8	116.349	NA	NA	NA	NA	NA	38.1
CaTMS748	(TGGA)7	(TGGA)6	ATCCACCTCCTTTTGCCTTT	CCTCAGTCTAGGTGGGCAGA	60.0	208	8	116.349	NA	NA	NA	NA	NA	53.3
CaTMS975	(ATA)27	(ATA)13	CAACAAAAATCCTTTTGCAAGTT	TTCCTGGATTTAGCATTCACA	60.0	271	8	116.349	NA	NA	NA	NA	NA	50.0
CaTMS623	(AAG)8	(AAG)10	CGGCCATTGAAATTGAAAA	GTTGGAAACAAACACGCCTT	60.0	276	8	116.349	Used	0	1	NA	Class I	50.7
CaGMS1277	(TA)9	(TA)6	AGGGAAACTTGACCCTCCAT	TGTTTGAAATTGCCACAAGC	60.0	191	8	116.349	Used	0.4	2	NA	Class II	33.3
CaGMS1167	(AT)7(AG)8	(AT)6	TCACCAACTCGTTGTTTACCTTT	TCAATAAACAAATTCAACATTCAAG	60.0	271	8	116.353	NA	NA	NA	NA	NA	33.3
CaTMS934	(ATT)24	(ATT)5	TTCAAACATCCTTTTTCTTTGGA	GGGCGTAAATGAATATTTTTCTAAT	60.0	226	8	116.357	NA	NA	NA	NA	NA	55.0

*Markers identity	Microsatellite repeat-motifs in ICC4958	Microsatellite repeat-motifs in PI489777	Forward primer sequences (5'-3')	Reverse primer sequences (5'-3')	Actual annealing temperature (0C)	Size (bp) of alleles amplified	Linkage groups (LGs)	Genetic positions (cM)	Markers used for polymorphism survey	Polymorphism information content (PIC)	Number of alleles amplified	Markers used in Figures	Markers types	GC%
CaGMS230	(ATT)11	(ATT)6	CGAACTCAGCAATTCAGCAA	AAATTATCAGCCACCTGCAGTAA	60.0	146	NA	NA	NA	NA	NA	NA	NA	33.3
CaGMS231	(TAT)16	(TAT)11	CAAAAATAAAAGGCGAAATTGA	TCGACTTTTAAAATAAACGGATCA	58.0	145	NA	NA	NA	NA	NA	NA	NA	42.9
CaGMS256	(AT)14	(AT)7	GGTTGAATTATTGAAATTCATCGTTA	CAAATTTCGCATTTGTGTTTG	60.0	224	NA	NA	NA	NA	NA	NA	NA	47.6
CaGMS257	(AT)13	(AT)6	AAAGTGAGTTCACGGCCAAC	GGGATGAAGAAACTCACCACA	60.0	145	NA	NA	NA	NA	NA	NA	NA	38.1
CaGMS258	(TAT)14	(TAT)7	TCTGCAATAGACTCAAGCTAAATCA	AATAGGGGGACCAAAAGTGC	60.0	252	NA	NA	NA	NA	NA	NA	NA	38.1
CaGMS260	(TA)13	(TA)6	CCTGCATCCATGAATCAACA	CACATGGAGCATTCCCACTA	60.0	241	NA	NA	NA	NA	NA	NA	NA	47.6
CaGMS261	(AT)14	(AT)7	CCGATTGCATAACTGCAAAA	TATCGCACAAAGAGAGTGCC	60.0	238	NA	NA	NA	NA	NA	NA	NA	38.1
CaGMS262	(TA)13	(TA)6	TGATGGAGTAATCGCTCTGAAA	ACCGTTGGAAATCGTCAATC	60.0	262	NA	NA	NA	NA	NA	NA	NA	33.3
CaGMS263	(AT)13	(AT)6	TTGAAACTTTTGCAAAGATACTAATTT	TGTTTATCCATCCCATTGAACA	58.0	219	NA	NA	NA	NA	NA	NA	NA	47.6
CaGMS264	(TA)14	(TA)7	TCATTTTCTACTGGGCCACC	CACAATCATTTCTTCAACCCG	60.0	265	NA	NA	NA	NA	NA	NA	NA	40.9
CaGMS265	(AT)13	(AT)6	TTCGAGTTTGTAAATACGACTTCC	GCCAGGCCAGACCATAAGTA	59.0	203	NA	NA	NA	NA	NA	NA	NA	33.3
CaGMS266	(TA)13	(TA)6	TTTTTGATATGCATGGTCGTT	GGACTCGCAATTTATTTTAGGAA	58.0	232	NA	NA	NA	NA	NA	NA	NA	38.1
CaGMS267	(AT)13	(AT)6	CATGAAGAGCTTTTGTGTGTGA	TGTGGCGCCTTAATAAGTTTC	59.0	128	NA	NA	NA	NA	NA	NA	NA	38.1
CaGMS268	(AT)13	(AT)6	AACCACGTCGCATAACATGA	TTTTAATTTGACGGTTCGCTT	60.0	174	NA	NA	NA	NA	NA	NA	NA	33.3
CaGMS270	(AT)14	(AT)7	TGACCAAAACAGATTACTCCAAA	TCATGCTAACATGTGTTCCAAA	59.0	279	NA	NA	NA	NA	NA	NA	NA	38.1
CaGMS271	(AT)13	(AT)6	TTGCTTCTTATAATGGCGGC	CGCTTGTCATAAAAGCGTTG	60.0	248	NA	NA	NA	NA	NA	NA	NA	47.6
CaGMS272	(TAA)11	(TAA)7	TGGTATAAAATCAGGGGCAAA	CGTTCATTTCCCGTAAGCAT	59.0	242	NA	NA	NA	NA	NA	NA	NA	40.9
CaGMS273	(AT)14	(AT)8	TCAATCCTAAATCATGAACAAATCA	TGGTCCATATTTACTCCTCTTACTTG	60.0	209	NA	NA	NA	NA	NA	NA	NA	38.1
CaGMS274	(AAT)10	(AAT)6	TCCCTATGCCAAATAGAATTACG	GGTCATCTCTTATTAGATGGTTATTCA	59.0	258	NA	NA	NA	NA	NA	NA	NA	38.1
CaGMS275	(AT)12	(AT)6	CAACATTTCATATGATCCCTTTCA	GGGACATTTGAGTCTTTGTTAGG	60.0	270	NA	NA	NA	NA	NA	NA	NA	42.9
CaGMS276	(AAT)9	(AAT)5	CAACTTCGTCATTTATTTTGTCAC	CGTTAAATTAAAGAAAACAAATACCAC	58.0	148	NA	NA	NA	NA	NA	NA	NA	38.1
CaGMS277	(TC)18	(TC)12	TTGAGGCTCTCTCGTTTGGT	CGAATTTGTTTCGGTTCGAT	60.0	243	NA	NA	NA	NA	NA	NA	NA	33.3
CaGMS278	(ATA)10	(ATA)6	TTTTCTCTCGTGCATTTCACA	CATGTAAAGAGAAATGAAATCAGCA	59.0	144	NA	NA	NA	NA	NA	NA	NA	33.3
CaGMS279	(ATA)10	(ATA)6	GATGCATTCTCAAAATGGTCAA	CCTCAAACGTAAAAGGCACC	60.0	251	NA	NA	NA	NA	NA	NA	NA	33.3
CaGMS281	(ATT)15	(ATT)9	AATGTGGTTGAGTAAATGTGTCA	AACGTGTAGAGTAGACGTGCTAAAA	57.0	203	NA	NA	NA	NA	NA	NA	NA	33.3
CaGMS282	(TA)12	(TA)6	AATCACAATAAAACCCTTCAAAAA	TTGAGCCCCTTCGATATAAA	58.0	280	NA	NA	NA	NA	NA	NA	NA	42.9
CaGMS283	(AT)12	(AT)6	AGCGTGATCACATTCACAGC	TCCTGAAGTCAATAGATTTTCATCC	60.0	254	NA	NA	NA	NA	NA	NA	NA	30.4
CaGMS284	(TAA)11	(TAA)7	CCGGAAATTTCAGAATGAATG	ACACGTTTTGTTTGTGGCAA	59.0	180	NA	NA	NA	NA	NA	NA	NA	42.9
CaGMS285	(GAA)11	(GAA)7	TTGCACCAATATCAAATGAAGG	CCTGTTGACAACTGAGAAGAGC	60.0	271	NA	NA	NA	NA	NA	NA	NA	47.6
CaGMS286	(AT)12	(AT)6	CGTTATAAAATGAAATTTGTTTGGAA	GAGAACACGGATGTAATTTAGCC	60.0	259	NA	NA	NA	NA	NA	NA	NA	38.1
CaGMS287	(AAT)11	(AAT)7	CACTGAAATATGTTGAGGAGACG	AGTTTGTGACTGCCTTTTCCA	59.0	207	NA	NA	NA	NA	NA	NA	NA	45.0
CaGMS288	(TA)13	(TA)7	CGCCTAGTGTTTAATTGGGC	TCTCGTCATCTTTTTCCTCCA	60.0	247	NA	NA	NA	NA	NA	NA	NA	38.1
CaGMS290	(TA)12	(TA)6	CAATCATCCATCGAATTCAAAA	GCATGTTATCTCAACTCATTAATCATC	60.0	186	NA	NA	NA	NA	NA	NA	NA	38.1
CaGMS291	(ATG)9	(ATG)5	ACGCTACTCGATGACGAGGT	TCGCATAGAAGATGTCGTCG	60.0	152	NA	NA	NA	NA	NA	NA	NA	33.3
CaGMS292	(AT)12	(AT)6	GACAAAAATCAACCATTACAAATGA	TGTCGATTGGATGAGTTCAA	59.0	258	NA	NA	NA	NA	NA	NA	NA	38.1
CaGMS293	(TA)12	(TA)6	TGCCCATAAAATTCCATTTACA	TTTTCTTAAAAACTTTGAATGTACCG	59.0	209	NA	NA	NA	NA	NA	NA	NA	33.3
CaGMS294	(TAA)12	(TAA)8	GATGAAAACAATTCGAACGGA	TCAGATTTAACGATTATCAATTTACCA	60.0	258	NA	NA	NA	NA	NA	NA	NA	38.1
CaGMS295	(AT)12	(AT)6	ACCATTCCACTTTTGGCTTG	TGAGATGTCTTGATATGGACCAA	60.0	279	NA	NA	NA	NA	NA	NA	NA	38.1
CaGMS296	(TA)12	(TA)6	GATCGGACCGACTGATTCAA	TCAGTCGTGCATCATAAAGG	61.0	120	NA	NA	NA	NA	NA	NA	NA	47.6
CaGMS297	(TA)12	(TA)6	ATGCCATTCTTGCCTGATTT	TCAAATGCAGCAACAACAAA	60.0	269	NA	NA	NA	NA	NA	NA	NA	42.9
CaGMS298	(ATT)14	(ATT)10	TCGCATTTTAAGGGAGGAAA	AGCAAGGAAGCATCATCCAA	60.0	212	NA	NA	NA	NA	NA	NA	NA	38.1
CaGMS299	(AT)12	(AT)6	TGAGTGCTTCTGTTTATGCATGT	TTGGACTTTGGACAGCACAG	60.0	208	NA	NA	NA	NA	NA	NA	NA	36.4
CaGMS300	(AT)12	(AT)6	TGTTGACGACAAATCACAATCA	TGAAAATTAATCTATTGAACTACGGA	60.0	180	NA	NA	NA	NA	NA	NA	NA	33.3
CaGMS302	(TA)12	(TA)6	GGAGTCGAACGATTCTCACC	TTGAGGGTGATAGTTAGAAAGAGACA	60.0	207	NA	NA	NA	NA	NA	NA	NA	42.9
CaGMS303	(AAT)14	(AAT)10	CAATTTCGTGAATGTTGGGTT	AAACGCCAAACATCTTAAATCA	60.0	261	NA	NA	NA	NA	NA	NA	NA	42.9
CaGMS304	(ATAA)8	(ATAA)5	TGTCTTCAGAATTAGTCTTCAAACAGA	TTCAATCTCATTTTATTTGTCTCCA	60.0	248	NA	NA	NA	NA	NA	NA	NA	42.9
CaGMS305	(TTC)9	(TTC)5	TGGTGTTGACTGATCGTTCG	AGGATGAATGTGGTGTTGAAGA	61.0	240	NA	NA	NA	NA	NA	NA	NA	42.9
CaGMS306	(TTA)16	(TTA)12	TCAATGTCAAGCTATATGACCTTCT	GCAACTGAGCTGAGAGCGTA	58.0	198	NA	NA	NA	NA	NA	NA	NA	42.9
CaGMS307	(AT)13	(AT)7	GATGAGTGCATGAGGAAGCA	CAACGATAACATTCATATTCTCCA	60.0	246	NA	NA	NA	NA	NA	NA	NA	42.9
CaGMS308	(TAT)13	(TAT)9	CTGTTTTGGAACAATTGGGG	TGAAGGCATTCAGCATTTTG	60.0	182	NA	NA	NA	NA	NA	NA	NA	42.9
CaGMS309	(AT)12	(AT)6	TTTGAACGCAATTTGGATGA	AGATGGTTAACGCAATTCAAA	60.0	196	NA	NA	NA	NA	NA	NA	NA	33.3
CaGMS310	(TC)12	(TC)6	CCCCTCCATCTACTGTTTCC	AAACCTAGCTTGGCAAAAACC	58.0	252	NA	NA	NA	NA	NA	NA	NA	42.9
CaGMS312	(AAT)16	(AAT)12	AAGGCGAAAATGTGAAGTTGA	GCAAACTTTTCTCACCTTAGCC	60.0	273	NA	NA	NA	NA	NA	NA	NA	33.3

*Markers identity	Microsatellite repeat-motifs in ICC4958	Microsatellite repeat-motifs in PI489777	Forward primer sequences (5'-3')	Reverse primer sequences (5'-3')	Actual annealing temperature (0C)	Size (bp) of alleles amplified	Linkage groups (LGs)	Genetic positions (cM)	Markers used for polymorphism survey	Polymorphism information content (PIC)	Number of alleles amplified	Markers used in Figures	Markers types	GC%
CaGMS313	(AT)12	(AT)6	ATGGGTATAAGTACCCGCGA	TGTGTCCATCCCTAAAACGA	59.0	223	NA	NA	NA	NA	NA	NA	NA	38.1
CaGMS314	(TATT)8	(TATT)5	TTTTTAAGCTTGTAGATCCCTTTT	TGTTCAAACCTGGGTGTAAGG	57.0	220	NA	NA	NA	NA	NA	NA	NA	33.3
CaGMS315	(TAT)10	(TAT)6	CACCCTCCCTCCCTTAAGTC	GCTGGATGGTCTGCAATTTT	60.0	255	NA	NA	NA	NA	NA	NA	NA	42.9
CaGMS317	(AAT)14	(AAT)10	TGTTGACACCTAATTTTGTCCG	AGAGGCAAACAAGAACCGAA	60.0	198	NA	NA	NA	NA	NA	NA	NA	42.9
CaGMS318	(AT)12	(AT)6	ATCCATGTATCCAACCCCAC	CCCTGCTATTGCAGAGGTGT	59.0	247	NA	NA	NA	NA	NA	NA	NA	42.9
CaGMS319	(TGA)9	(TGA)5	TCATGGATTTCCTTTGCCTC	CGCAACTTCTCCCTTTCATC	60.0	248	NA	NA	NA	NA	NA	NA	NA	42.9
CaGMS320	(TA)12	(TA)6	CATTGCATTGCATAGTCAAACA	GCTGCAACTTGGGGATGATA	60.0	255	NA	NA	NA	NA	NA	NA	NA	42.9
CaGMS321	(TA)13	(TA)7	TGCAACACATTTTCAGCCTT	CTTCATAGCATAGTCGCCCG	59.0	119	NA	NA	NA	NA	NA	NA	NA	42.9
CaGMS322	(AT)9	(AT)6	TTAGGCGCCACTCACTTTTT	TGTTCATTTGCATCGGAAAC	60.0	243	NA	NA	NA	NA	NA	NA	NA	33.3
CaGMS323	(AT)10	(AT)6	GAGGTTGGGGTTGGTTTCTT	CCATTCGCCTTCAATTTTGT	60.0	208	NA	NA	NA	NA	NA	NA	NA	42.9
CaGMS324	(TA)13	(TA)7	AACCACGACCATCTCTTTGG	GGGTCTTGGAGCCCATACTT	60.0	169	NA	NA	NA	NA	NA	NA	NA	38.1
CaGMS325	(ATT)9	(ATT)5	GGGGTCCACTTTCATTTGTG	TTTGGATGCCAACATAGTTCA	60.0	259	NA	NA	NA	NA	NA	NA	NA	40.0
CaGMS327	(AT)13	(AT)7	ATTAAATTGGGCCTTTTGCC	TGATGAAATTTTGTGATGCATTCT	60.0	225	NA	NA	NA	NA	NA	NA	NA	33.3
CaGMS328	(TTA)10	(TTA)6	AAACCATGTCGTCCGTCAA	TGGAATGACTACTGAGTGATTGTC	60.0	280	NA	NA	NA	NA	NA	NA	NA	38.1
CaGMS329	(AT)12	(AT)6	CGATTTAACAAGGATTAAAGGCA	GCATTCTTCTCAATATAAGACTTTGA	60.0	242	NA	NA	NA	NA	NA	NA	NA	38.1
CaGMS330	(TA)13	(TA)7	AATGGAAAGCATCCAAGCAC	AAGGCCAATTTTGTTTGACG	60.0	199	NA	NA	NA	NA	NA	NA	NA	38.1
CaGMS331	(AT)12	(AT)6	CTCATCGGTCACTGGTTCAA	TCTGAACCAAACCCAGTTCC	60.0	178	NA	NA	NA	NA	NA	NA	NA	42.1
CaGMS332	(AT)13	(AT)7	GATTGGTATCTGGCCATCAAA	TGCAAAACCGCATTCTAGTG	60.0	266	NA	NA	NA	NA	NA	NA	NA	47.6
CaGMS333	(TA)13	(TA)7	ATTTAACACCGTTAACCACACA	CCGATAAGGCAGCATACGAT	57.0	270	NA	NA	NA	NA	NA	NA	NA	42.9
CaGMS334	(TTA)14	(TTA)10	GGCTGAAGAAGAAAATCATCG	CCTTGACCCTACAAGCCAAG	59.0	227	NA	NA	NA	NA	NA	NA	NA	33.3
CaGMS335	(TTA)15	(TTA)11	TTTTAACGTGCGTCACCAAC	TTGGAGTGCATGAACCTGAC	60.0	233	NA	NA	NA	NA	NA	NA	NA	33.3
CaGMS336	(AT)14	(AT)8	GTCCCCCGAATGTGTAGAGA	CTGGTCCTTTTTGGGTCTTG	60.0	256	NA	NA	NA	NA	NA	NA	NA	47.6
CaGMS337	(AAT)10	(AAT)6	TGATTTCTCCATCTTATCGGG	ATTAGCGCCTTACGACATCC	59.0	259	NA	NA	NA	NA	NA	NA	NA	33.3
CaGMS338	(TAT)9	(TAT)5	CTCAATTTCTGTTTGGGGGA	CTCACCGTACATTGCAGCAT	60.0	252	NA	NA	NA	NA	NA	NA	NA	35.0
CaGMS339	(CTT)9	(CTT)5	GCATCACAAGCTTCAACAGC	TTTTGGGTTGATGGGTGATT	60.0	213	NA	NA	NA	NA	NA	NA	NA	45.0
CaGMS340	(AT)13	(AT)7	TCAGCAATGAAAATAACTATCCAG	TTGTGGTTGCATCCCTAACA	58.0	189	NA	NA	NA	NA	NA	NA	NA	42.9
CaGMS341	(AT)12	(AT)7	TGCAAGAGTATTGAAACCAAACTG	TCATGCGTTGATGTCTAATTTTG	61.0	254	NA	NA	NA	NA	NA	NA	NA	38.1
CaGMS343	(GA)12	(GA)7	CTTGCGTATGACGAGGTTGA	TCTCTGTCGGCATCTTTGC	60.0	232	NA	NA	NA	NA	NA	NA	NA	38.1
CaGMS344	(AT)11	(AT)6	TCTTGATAATTGATGGGTGGAA	AAATAAGTTGCAAATCTTCATAGGATA	59.0	236	NA	NA	NA	NA	NA	NA	NA	38.1
CaGMS345	(AT)11	(AT)6	ACATTCGAACCTTGGCAGTT	ATGGAGAAGCCTGAAAACCA	60.0	258	NA	NA	NA	NA	NA	NA	NA	42.9
CaGMS346	(AT)11	(AT)6	GGCCGATCCAACAAGTTAAA	TCATGTCTATGGTCAAATTCAGA	60.0	279	NA	NA	NA	NA	NA	NA	NA	42.9
CaGMS347	(AT)11	(AT)6	TTTTCTTTTGTATTTGAGACCCA	GCCGTCCTCATGTAGACCAT	58.0	236	NA	NA	NA	NA	NA	NA	NA	42.9
CaGMS348	(AT)11	(AT)6	TCAATTATTGGGTTGTCTTATGTC	GGGAGATTAGACGTTCGGTC	57.0	237	NA	NA	NA	NA	NA	NA	NA	42.9
CaGMS349	(AT)11	(AT)6	AACACTTCCACACGTCCTCC	CAAGGTACATATGTGTTATTTAACCG	60.0	233	NA	NA	NA	NA	NA	NA	NA	47.6
CaGMS350	(TA)12	(TA)7	AGAATGCTATCCAATTGCCC	AAAAATTATGTACGCATCCAATCA	59.0	280	NA	NA	NA	NA	NA	NA	NA	42.9
CaGMS352	(TC)11	(TC)6	GGCAAAAGAAAAACATCGGA	TTCTGGAAAAAGGAGAGCGA	60.0	235	NA	NA	NA	NA	NA	NA	NA	33.3
CaGMS353	(TTA)12	(TTA)7	ATTCCAGGTGTGTGGCTGAT	TCTCCAATCAGTTCAACTCAAAA	60.0	244	NA	NA	NA	NA	NA	NA	NA	34.8
CaGMS354	(AT)11	(AT)6	CATACCATATGCACCCCCTC	AAGGTGACATAATAGTGCAACAATC	60.0	164	NA	NA	NA	NA	NA	NA	NA	38.1
CaGMS355	(AT)12	(AT)7	CGTTTTTCATATTCAGGCCC	CGCATGCATACGGAACTCTA	59.0	187	NA	NA	NA	NA	NA	NA	NA	42.9
CaGMS356	(AT)11	(AT)6	CCCTTCACTTTCTCACATAAAGG	AAGCCCTGTAACTCCAGTCG	59.0	195	NA	NA	NA	NA	NA	NA	NA	42.9
CaGMS357	(AT)12	(AT)7	CCGACCTGAACATTTTATTGC	CAAAGTATACAATCTACCAATCAATCA	59.0	168	NA	NA	NA	NA	NA	NA	NA	33.3
CaGMS359	(AT)11	(AT)6	AACGAGTTAGGTTCGTCTCATGT	TGACATGACAATTTGCAACAA	59.0	207	NA	NA	NA	NA	NA	NA	NA	33.3
CaGMS360	(TA)11	(TA)6	TTATTGGCGTGCATGTTTTT	CAGAGTATGCTGAATTGGAGTTG	59.0	257	NA	NA	NA	NA	NA	NA	NA	42.9
CaGMS361	(TA)11	(TA)6	TGGATTTCTATAAAATTTTGTGTGTTT	GTTTGTCGGGTGGAGACAAT	59.0	208	NA	NA	NA	NA	NA	NA	NA	42.9
CaGMS362	(AT)12	(AT)7	AACACGAGGGACTAATGTTTGATT	AAGTCAAATATTCCTTCTCGAACA	60.0	208	NA	NA	NA	NA	NA	NA	NA	38.1
CaGMS363	(TA)12	(TA)7	CAGAAAAATGTTGGGTGTTTTT	CAAGACTTTAGTATGGGAATATCACG	58.0	105	NA	NA	NA	NA	NA	NA	NA	33.3
CaGMS364	(TA)12	(TA)7	TGCTTCTTAAATTCCGCCTT	TCTCTTTTTCCTCAATGCTTCA	59.0	248	NA	NA	NA	NA	NA	NA	NA	38.1
CaGMS366	(AT)11	(AT)6	CCATGTCCATTTAATTGCTGA	AACCCCACACACACACACAC	58.0	233	NA	NA	NA	NA	NA	NA	NA	47.6
CaGMS367	(AT)12	(AT)7	TGGTTAGGAAAATGTTTGATCG	TAAATGAAGACGCGCAACAG	59.0	176	NA	NA	NA	NA	NA	NA	NA	33.3
CaGMS368	(AT)11	(AT)6	TGCGAGATGAATTTGGATCTT	GCATCCCAACACATTTCAAAG	60.0	276	NA	NA	NA	NA	NA	NA	NA	33.3
CaGMS370	(TA)11	(TA)6	ACTAATGCCAATCGCTCAGG	ATGTGATGCGTGGTTGAGTC	60.0	267	NA	NA	NA	NA	NA	NA	NA	38.1
CaGMS372	(TA)11	(TA)6	TGCCTTTTGAGTTACGTGACAA	ATAAGGCGATCTCCCCAACT	61.0	206	NA	NA	NA	NA	NA	NA	NA	42.9
CaGMS373	(GA)14	(GA)9	CCGGAGAGAGTGAAAGCATC	CCATTGCACCATGAGAGAGA	60.0	150	NA	NA	NA	NA	NA	NA	NA	38.1

*Markers identity	Microsatellite repeat-motifs in ICC4958	Microsatellite repeat-motifs in PI489777	Forward primer sequences (5'-3')	Reverse primer sequences (5'-3')	Actual annealing temperature (0C)	Size (bp) of alleles amplified	Linkage groups (LGs)	Genetic positions (cM)	Markers used for polymorphism survey	Polymorphism information content (PIC)	Number of alleles amplified	Markers used in Figures	Markers types	GC%
CaGMS374	(AT)11	(AT)6	AAACAAGTTGGTTTGATTTGATTG	TCACCGAAAACCTGAACAAA	59.0	211	NA	NA	NA	NA	NA	NA	NA	38.1
CaGMS375	(AT)11	(AT)6	CAATGTTCAAAATTTGCTTTTCAT	AAACTCATAAGAAAAATCATTACCAAT	59.0	149	NA	NA	NA	NA	NA	NA	NA	38.1
CaGMS376	(TA)11	(TA)6	CAAGCTCAGTTTCACAAAATGC	CCATCCATATATGATTATGTGGTCA	60.0	242	NA	NA	NA	NA	NA	NA	NA	38.1
CaGMS378	(AT)11	(AT)6	TCCATTTTGATGTAACCATTGA	TGTTTTTGTTTTATTTTTGGCTTC	58.0	208	NA	NA	NA	NA	NA	NA	NA	38.1
CaGMS380	(TA)12	(TA)7	CACGTGTGTGCAAAAGTGTGT	CTGCTCGAGACCCTTAGAGTG	61.0	211	NA	NA	NA	NA	NA	NA	NA	33.3
CaGMS381	(TA)12	(TA)7	TCCTGGTATGGTAGTAATATTGTGTTT	TCCTTCACACACATCAGTCAAA	58.0	271	NA	NA	NA	NA	NA	NA	NA	38.1
CaGMS382	(AT)11	(AT)6	TTTGATAGGATTTCAACCACGA	TCAAAGCGATTTACTCAACCAA	59.0	269	NA	NA	NA	NA	NA	NA	NA	38.1
CaGMS383	(AT)11	(AT)6	TCGACAAATAATTTTAAAACGGG	TTTGTGTTGAATTGTTGTGACG	59.0	163	NA	NA	NA	NA	NA	NA	NA	38.1
CaGMS384	(TA)12	(TA)7	TCAAGCAGGGAATGGAGAAC	TTGAATTTAAAATAATGTTGGCAAT	60.0	206	NA	NA	NA	NA	NA	NA	NA	38.1
CaGMS385	(AT)11	(AT)6	TCTTGCCACCAACAAAACAA	TGTTCCAATTCCTCGTGTCA	60.0	211	NA	NA	NA	NA	NA	NA	NA	38.1
CaGMS387	(AT)12	(AT)7	AATTCAAAATGGATCACGGG	TTTAATCTATAACGGGAAAACGTAA	60.0	227	NA	NA	NA	NA	NA	NA	NA	47.6
CaGMS388	(TTA)10	(TTA)5	AGGGAAAGAAGGCAACTCGT	GCATGCAATGGATGATGAAG	60.0	276	NA	NA	NA	NA	NA	NA	NA	52.4
CaGMS389	(AT)11	(AT)6	GGATGAAAATCTCGTGTTGC	GGAGACCGAATTTTGAATGAA	58.0	201	NA	NA	NA	NA	NA	NA	NA	33.3
CaGMS390	(AT)11	(AT)6	TGTCAAACATTCAACTATCGACAT	TTGACTCGTTGTTGCTCTGG	59.0	229	NA	NA	NA	NA	NA	NA	NA	42.9
CaGMS391	(AT)11	(AT)6	GAATCTCACAGTTGTGTGAGTTGAT	AAATTGGTGGAAAAAGCCAA	60.0	251	NA	NA	NA	NA	NA	NA	NA	33.3
CaGMS392	(TA)11	(TA)6	AATCCCCAAAATCTTGCTCA	TTGCTTTTGAATGGATGACG	60.0	249	NA	NA	NA	NA	NA	NA	NA	33.3
CaGMS394	(TA)11	(TA)6	AAAGGCTGCCACTAATATCGAA	CACCTGAAAACCAAGAATTAGGA	60.0	231	NA	NA	NA	NA	NA	NA	NA	42.9
CaGMS395	(TA)11	(TA)6	TATCTCCACTCCCTCGTTCC	TGTTCTAGCAGGACCCTTCC	59.0	197	NA	NA	NA	NA	NA	NA	NA	47.6
CaGMS396	(TA)11	(TA)6	AAATCGTGTTTATGGGTTAGACA	GTTATCGATCAGCAGTGCCA	58.0	122	NA	NA	NA	NA	NA	NA	NA	42.9
CaGMS397	(AT)11	(AT)6	TGTAACTAAATTGTCACCCGCA	TCAAGATGGATGATTTTAGACCAA	60.0	152	NA	NA	NA	NA	NA	NA	NA	38.1
CaGMS398	(TA)12	(TA)7	AGATTGCCTTGGTCGAAAGA	TTGGTTCACACCTCATCATCA	60.0	198	NA	NA	NA	NA	NA	NA	NA	38.1
CaGMS399	(AT)11	(AT)6	ATTGTTGGCAGGTTGTGGTT	CCGATTGTAGTGGGGCTAAT	60.0	230	NA	NA	NA	NA	NA	NA	NA	31.8
CaGMS400	(AT)11	(AT)6	AGCCAGGATGTAGTGGATGG	TTGGTTGTGCTTGTTTTCCA	60.0	250	NA	NA	NA	NA	NA	NA	NA	33.3
CaGMS401	(TA)11	(TA)6	CCCATAGAGAGCCCAACAAG	GGTCCTCTTTCTTCCGTCG	60.0	252	NA	NA	NA	NA	NA	NA	NA	42.9
CaGMS431	(AT)12	(AT)8	CTAGGGTTTACGCAGAAGCG	TCAATCCTAAATCAGGAATAAATCAA	60.0	272	NA	NA	NA	NA	NA	NA	NA	45.0
CaGMS432	(AT)10	(AT)6	AAAGAACTTGAGGCAGCCAA	TCTTTTCAAACGAATGCAATTT	60.0	264	NA	NA	NA	NA	NA	NA	NA	42.9
CaGMS434	(AT)11	(AT)7	ACATTTTATCGGTGTAACATTAACATA	TCATGGAGATGGACCTGACA	57.0	249	NA	NA	NA	NA	NA	NA	NA	38.1
CaGMS435	(AT)10	(AT)6	CAACTGCAAAATTCAAATACGTT	AAATCTATTTGAGCAAGAGTGTTTTT	58.0	210	NA	NA	NA	NA	NA	NA	NA	33.3
CaGMS436	(TTTA)7	(TTTA)5	ATTTTATGGCCAACCCAACA	ACTCGGCTTCCATTGATGAG	60.0	174	NA	NA	NA	NA	NA	NA	NA	42.9
CaGMS437	(AT)10	(AT)6	TTTTGAACTGCAGGATGTTCC	AGGTTGCCTTCAACAAAGGA	60.0	234	NA	NA	NA	NA	NA	NA	NA	42.9
CaGMS438	(AT)12	(AT)8	GGGTATTTTGTTTAGAAAAATGTTCG	TCCTGAAAGCAGAGAGTTGGA	60.0	219	NA	NA	NA	NA	NA	NA	NA	38.1
CaGMS439	(AT)11	(AT)7	TTTTTGTTTTTGTCAATGTAATATGC	AAGCGAGAACTTACGGAACC	59.0	244	NA	NA	NA	NA	NA	NA	NA	38.1
CaGMS440	(TA)10	(TA)6	TGTCAGCATGGAATGAGAATG	GCAAGAATGCACCCTCTCTC	60.0	120	NA	NA	NA	NA	NA	NA	NA	50.0
CaGMS441	(AT)11	(AT)7	TCTTAGCTTTTCCCCTGTCG	CCCACTGTCATCAACCTCCT	59.0	204	NA	NA	NA	NA	NA	NA	NA	40.0
CaGMS442	(AT)10	(AT)6	GGGAACTCACACAGTTAAAGCA	CATGAAAAAGGAAGGGAAAAA	59.0	262	NA	NA	NA	NA	NA	NA	NA	38.1
CaGMS443	(TC)11	(TC)7	CAGTGCCATCACTCCATCAC	CACGCAAAAACAAACCACAG	60.0	252	NA	NA	NA	NA	NA	NA	NA	38.1
CaGMS444	(AT)10	(AT)6	CATAGTTGTTGAGTTTCAAATGGTG	ATGCATGTGCTTCCATACCA	60.0	232	NA	NA	NA	NA	NA	NA	NA	42.9
CaGMS445	(AG)12	(AG)8	GGTTTGATGCTACCCCAAAA	TGTGACCCCAATACTCCAAA	60.0	225	NA	NA	NA	NA	NA	NA	NA	38.1
CaGMS446	(TA)11	(TA)7	CAGAGGGCGACTTCCATAAA	AATACAATTGGGGTTCAGCG	60.0	253	NA	NA	NA	NA	NA	NA	NA	33.3
CaGMS447	(AT)11	(AT)7	AATCATAACCGATAACAAAACTCAT	TCATTAGACTCCAACAATTCATAGAC	57.0	268	NA	NA	NA	NA	NA	NA	NA	42.9
CaGMS448	(TA)10	(TA)6	TTTGCAAATGGCTCACTCAT	TTCAAAATGAAATATTCTTCTTCCG	59.0	233	NA	NA	NA	NA	NA	NA	NA	42.9
CaGMS449	(AT)10	(AT)6	CCGCTTATTTTCGAGTTGGA	TTTGGTTATATTGATGTTTGTAATGAA	60.0	278	NA	NA	NA	NA	NA	NA	NA	38.1
CaGMS450	(AT)11	(AT)7	TCCAATGTTGGGGAATTCAT	TTGGATTTCAATCTCATAACTGGA	60.0	191	NA	NA	NA	NA	NA	NA	NA	33.3
CaGMS451	(AT)10	(AT)6	TGGAAGGAAAATGAAAGAGGT	GACCATCTATGTCCAGACACCA	58.0	175	NA	NA	NA	NA	NA	NA	NA	50.0
CaGMS452	(AC)10	(AC)6	TGGCATCACAAGATTTCAATG	GCACTGTCATGAGAAAGCCA	60.0	251	NA	NA	NA	NA	NA	NA	NA	47.6
CaGMS454	(TA)10	(TA)6	GTGACGGCCTCCTTAACAAC	TTTTGGTGGAACACTTGCCT	60.0	264	NA	NA	NA	NA	NA	NA	NA	42.9
CaGMS455	(TA)10	(TA)6	TGTTATCATCTTTAATGTTTGATTAGG	AATGAGGGCATCACACCTTC	57.0	229	NA	NA	NA	NA	NA	NA	NA	40.9
CaGMS472	(TA)10	(TA)6	TCCTCCATTGCCTTTTCATC	GCATATTTTGGGACTAAGCATGT	60.0	229	NA	NA	NA	NA	NA	NA	NA	47.6
CaGMS474	(AT)10	(AT)6	CTGCAACAACGTTCCAAAAA	GGCCAGACTCACACTTATAAAAA	60.0	203	NA	NA	NA	NA	NA	NA	NA	42.9
CaGMS475	(AT)10	(AT)6	CAAATCAATATACAACACATCACTCC	CTTGCTCCTACGTATCCCCA	59.0	215	NA	NA	NA	NA	NA	NA	NA	38.1
CaGMS476	(AT)10	(AT)6	TCACGTCCATATTAATCATCAATTC	AGTGATAACCGTTGGAAAAGC	59.0	280	NA	NA	NA	NA	NA	NA	NA	33.3
CaGMS477	(TC)10	(TC)6	TGGATCCATTTGGTTTGGAT	TTCTTTCTCGTCGACTCCGT	60.0	226	NA	NA	NA	NA	NA	NA	NA	33.3
CaGMS479	(AT)11	(AT)7	CCACGTAAATAGCCGAGCAA	TCGCTTTTGCGTTTATGTTG	61.0	251	NA	NA	NA	NA	NA	NA	NA	42.9

*Markers identity	Microsatellite repeat-motifs in ICC4958	Microsatellite repeat-motifs in PI489777	Forward primer sequences (5'-3')	Reverse primer sequences (5'-3')	Actual annealing temperature (0C)	Size (bp) of alleles amplified	Linkage groups (LGs)	Genetic positions (cM)	Markers used for polymorphism survey	Polymorphism information content (PIC)	Number of alleles amplified	Markers used in Figures	Markers types	GC%
CaGMS480	(TA)10	(TA)6	GCGTTAGCGTCCTTGACTTC	TGAAGTGGTATATCTTAACCCAGGA	60.0	257	NA	NA	NA	NA	NA	NA	NA	33.3
CaGMS481	(AT)10	(AT)6	TGTTGCTCCAGAAAGGGTTC	TCCGGATACCACCTTCATTC	60.0	178	NA	NA	NA	NA	NA	NA	NA	33.3
CaGMS482	(AT)10	(AT)6	ACCAAAAACCCATGGAGGAT	TGCTGATCAAGACAAATGGC	60.0	266	NA	NA	NA	NA	NA	NA	NA	33.3
CaGMS483	(AT)10	(AT)6	ATGCGTTATGGGTGAAATGG	TGGAGGAAGAAGGGAGAAGC	61.0	260	NA	NA	NA	NA	NA	NA	NA	33.3
CaGMS484	(AT)11	(AT)7	TTGTGGTTGTTGTGTTGTCAAA	TGCAAGTAATAGATTTTGGAGGC	60.0	182	NA	NA	NA	NA	NA	NA	NA	38.1
CaGMS486	(AT)11	(AT)7	TTCTTACTTCATCAAATCCGACA	TTCCATCTTCAAATAACCACTCAA	59.0	256	NA	NA	NA	NA	NA	NA	NA	38.1
CaGMS487	(AT)10	(AT)6	GGAATGAAATGATGGGGGTA	ATGGAGGGAACGTGTTTCTG	59.0	231	NA	NA	NA	NA	NA	NA	NA	38.1
CaGMS488	(AT)13	(AT)9	GGCTTGGTGGACCAATTTAG	TCCGTTAACTGGTGACAACG	59.0	250	NA	NA	NA	NA	NA	NA	NA	39.1
CaGMS489	(AT)10	(AT)6	TGTCTATTAATCCATCAACATTGGA	CAATTTTCTATTATTCCAAACAATGC	60.0	235	NA	NA	NA	NA	NA	NA	NA	47.6
CaGMS491	(TTTA)8	(TTTA)6	TGTGACATGATTTATGAATGACTTTTT	TTAGGGTTGTTCCAGTTGGC	60.0	207	NA	NA	NA	NA	NA	NA	NA	33.3
CaGMS492	(AT)10	(AT)6	TGATGAAAGCATGTTGACCA	TTCAAAGTTATGCCAAAAGGAAA	59.0	226	NA	NA	NA	NA	NA	NA	NA	38.1
CaGMS493	(AT)11	(AT)7	TGAATTGCTTAGGTCTTTAGTCCA	TCTACTTTCGTCGCAACTCA	59.0	243	NA	NA	NA	NA	NA	NA	NA	33.3
CaGMS494	(TA)10	(TA)6	CATCCAACAAGGACGGAGTT	TTTTAGAACTTGGGTAGAACGG	60.0	165	NA	NA	NA	NA	NA	NA	NA	38.1
CaGMS495	(AT)10	(AT)6	TTAGCACGATTGGTAGCACG	TGGGCTTTGAGCTTCATTTT	60.0	119	NA	NA	NA	NA	NA	NA	NA	38.1
CaGMS496	(TA)11	(TA)7	CAATGTAAAACCCACCCATT	ATTGGAGAAACCTTTCCGCT	57.0	252	NA	NA	NA	NA	NA	NA	NA	33.3
CaGMS497	(TA)10	(TA)6	AACCCATCATTTTGCCATGT	CCTGGAAAACTTGCAACTGG	60.0	155	NA	NA	NA	NA	NA	NA	NA	30.0
CaGMS499	(AT)10	(AT)6	GAGGCTGAGAGGAGTACATTCG	ATCTTCTTAGGGAGCAGGGG	60.0	277	NA	NA	NA	NA	NA	NA	NA	38.1
CaGMS500	(AT)10	(AT)6	TGAAGCTAGGCTTGGTGGAT	ATTTTTCACCGACCGTTTTG	60.0	148	NA	NA	NA	NA	NA	NA	NA	42.9
CaGMS501	(AT)10	(AT)6	CGTCTTATGGCTTACCTCCC	TGTTGAATTGTTTAGGTTATTAGCCA	59.0	221	NA	NA	NA	NA	NA	NA	NA	33.3
CaGMS502	(TA)10	(TA)6	CCAAAATCTCAAATTGGCCT	TGCTTGTTTTCTTGCTGTGG	59.0	170	NA	NA	NA	NA	NA	NA	NA	42.9
CaGMS503	(AT)11	(AT)7	CTGTCAATTGAGTTGCGACG	AATGGGTTTGCGAGAAACAG	60.0	273	NA	NA	NA	NA	NA	NA	NA	42.9
CaGMS504	(TTAT)7	(TTAT)5	TTGTGAGGACTGAACGTAGCTT	GAAAACGGAGAACGCTCTTG	59.0	202	NA	NA	NA	NA	NA	NA	NA	42.9
CaGMS505	(TA)10	(TA)6	GGAGACAACTTCTCTTCACCAA	AGCCACTTCATTGACTTGTCTC	58.0	203	NA	NA	NA	NA	NA	NA	NA	42.9
CaGMS506	(TA)10	(TA)6	TGTCTTCAAAAGGATAACTCATGC	TCATTTTGAGTGTTGAAGATTATGG	60.0	207	NA	NA	NA	NA	NA	NA	NA	38.1
CaGMS509	(AT)10	(AT)7	TCTTTTACATAATCCTTAATCCTCACC	TTGCACCAAGATTCCAAAGA	59.0	277	NA	NA	NA	NA	NA	NA	NA	40.0
CaGMS558	(TA)10	(TA)7	GCGAGTGTCCAATCTCAACA	GAAGCGACCTAAACTTTTGTGAA	60.0	268	NA	NA	NA	NA	NA	NA	NA	42.9
CaGMS559	(TA)9	(TA)6	CTTCGATCCAACAGTCAATGAG	ATGAGTGGGGATCGATCTTG	60.0	264	NA	NA	NA	NA	NA	NA	NA	30.0
CaGMS560	(TA)10	(TA)7	CAACCACTCTAGGGTTGCAT	TGCATCATGAGTTTGAAGGG	58.0	169	NA	NA	NA	NA	NA	NA	NA	38.1
CaGMS561	(TA)9	(TA)6	TGAAGAGTGAAAATTTGAAGAAAAA	TGTTTTGTGAAAAATGTGGCA	59.0	116	NA	NA	NA	NA	NA	NA	NA	33.3
CaGMS563	(ATT)7	(ATT)5	TTGGAACAGACATTGACCCA	TTGGAGAAAACTTTGGACAACA	60.0	223	NA	NA	NA	NA	NA	NA	NA	33.3
CaGMS564	(ATT)7	(ATT)5	TGCTTTTCACATCATCCAATG	TGCTAAATTCATTACAAAACATTCAA	60.0	233	NA	NA	NA	NA	NA	NA	NA	33.3
CaGMS565	(AT)10	(AT)7	AATTAAGAAAATTGATGCATTTGA	CCAACATCAAGATGGATACGG	57.0	233	NA	NA	NA	NA	NA	NA	NA	33.3
CaGMS566	(AT)10	(AT)7	TGGAAGAGAAAGTAAGCAAAATCA	TTTTCACAAAAGCATTGCCA	59.0	246	NA	NA	NA	NA	NA	NA	NA	42.9
CaGMS567	(TAA)7	(TAA)5	GGCAAGAGAAGGTCGCATC	TGGCACATCTTCTCAACTATTTTT	61.0	280	NA	NA	NA	NA	NA	NA	NA	33.3
CaGMS568	(TA)10	(TA)7	TGTCGAAATGAATGAGAGCAA	TGCTAGTTGGTGGTTGTGGA	59.0	230	NA	NA	NA	NA	NA	NA	NA	38.1
CaGMS571	(TTA)7	(TTA)5	GCGCTTATGCAATCACTTCA	AAAGATGACGCCGACATTAAA	60.0	176	NA	NA	NA	NA	NA	NA	NA	42.9
CaGMS577	(TA)9	(TA)6	CACGAAATCGGAAGGGATTA	TGATGGAATTTTCAATTATCTCATTT	60.0	193	NA	NA	NA	NA	NA	NA	NA	42.9
CaGMS578	(TA)13	(TA)10	CAACCATACTTTCAAGTTTCACG	CATCCAGACCCCCTTCAGT	59.0	278	NA	NA	NA	NA	NA	NA	NA	33.3
CaGMS579	(TAT)7	(TAT)5	TCATTTAAGGGAAAAGGGGG	TGTGAGTGTGTCAAAGGGTTTT	60.0	279	NA	NA	NA	NA	NA	NA	NA	42.9
CaGMS580	(AT)9	(AT)6	TTGGAAGATGGATTGGTTGA	AATTGAGGGACCATTTCCGT	59.0	255	NA	NA	NA	NA	NA	NA	NA	42.9
CaGMS581	(AAT)7	(AAT)5	TTTCTGAAACAAACGCACTCA	TAAGCCTGATAGGCCAGCAT	59.0	178	NA	NA	NA	NA	NA	NA	NA	38.1
CaGMS584	(TTC)7	(TTC)5	CATTTACACTTCGCGATTCAA	CAACCATATGGGAGATGCAA	59.0	114	NA	NA	NA	NA	NA	NA	NA	42.9
CaGMS586	(AT)10	(AT)7	AACTTTTAACTTTTGGATGTTCCC	TGGACCAAATTAGTGAAATAATGG	59.0	158	NA	NA	NA	NA	NA	NA	NA	31.8
CaGMS590	(TA)10	(TA)7	CTAGGACCGACGACCAAGAG	TCGATAGCGAGGAGACATAGC	60.0	241	NA	NA	NA	NA	NA	NA	NA	38.1
CaGMS592	(TA)10	(TA)7	ATTGTTGCAAATTGGCCCTA	TTGTCCAAAAGTGAAATTGAAAA	60.0	128	NA	NA	NA	NA	NA	NA	NA	38.1
CaGMS594	(TG)9	(TG)6	TTGCCATCAATGGTGTGATT	TTTGTTTCTTGCTATTGGGTTTT	60.0	143	NA	NA	NA	NA	NA	NA	NA	42.9
CaGMS595	(TAA)9	(TAA)7	CAACTCTCTTCTATTTTTCCTCCAA	TCCTCATCGTACTTTGTATGTGG	59.0	214	NA	NA	NA	NA	NA	NA	NA	38.1
CaGMS608	(TTTA)7	(TTTA)5	CCCTCCCAACATCCTTTGTA	TTTAAAATGAGAGGATGGAGTATGTT	60.0	212	NA	NA	NA	NA	NA	NA	NA	38.1
CaGMS610	(TA)10	(TA)7	TCGAGGCACAAGGGAAAATA	TGAGCTGCGACGAGAGTAGA	61.0	188	NA	NA	NA	NA	NA	NA	NA	47.6
CaGMS614	(TA)8	(TA)6	TGGAATAACACCCAACTTGAAA	GAAGAAATGACCAAAATAGCCAA	59.0	234	NA	NA	NA	NA	NA	NA	NA	38.1
CaGMS615	(TA)9	(TA)6	TGTGTATAACTATTTCTGAATTGACCA	AAGTTATGAATCAAAAGCAAAATGAA	58.0	261	NA	NA	NA	NA	NA	NA	NA	42.9
CaGMS618	(TA)9	(TA)6	GGACAATAATCGTTGAATTAGCAA	AATGGTGGGTGTTGTGTGAA	59.0	249	NA	NA	NA	NA	NA	NA	NA	33.3
CaGMS619	(TAT)8	(TAT)6	CCGTTAAGAAACTCTTTCAAGCC	GCCTTTTTGTAAATCGACATGA	61.0	175	NA	NA	NA	NA	NA	NA	NA	33.3

*Markers identity	Microsatellite repeat-motifs in ICC4958	Microsatellite repeat-motifs in PI489777	Forward primer sequences (5'-3')	Reverse primer sequences (5'-3')	Actual annealing temperature (0C)	Size (bp) of alleles amplified	Linkage groups (LGs)	Genetic positions (cM)	Markers used for polymorphism survey	Polymorphism information content (PIC)	Number of alleles amplified	Markers used in Figures	Markers types	GC%
CaGMS620	(AT)11	(AT)8	AATGAATGCGTTTGTTTCAGA	TTTTGTCTTGTCTGGAAGCCT	58.0	252	NA	NA	NA	NA	NA	NA	NA	38.1
CaGMS621	(TA)11	(TA)8	CAAAATTTTCAAACAAAACCCA	TCTTTCAATCCAACGGACAA	59.0	244	NA	NA	NA	NA	NA	NA	NA	33.3
CaGMS623	(AT)9	(AT)6	ATGTGGAACAAACTACCGGC	CCGTTACCACAACCACAAAG	60.0	209	NA	NA	NA	NA	NA	NA	NA	31.8
CaGMS624	(AT)9	(AT)6	TCTTCACCATCCTATCTTTCATCA	CTCGATGTTACTTCTACATCTTCG	60.0	271	NA	NA	NA	NA	NA	NA	NA	33.3
CaGMS627	(AT)9	(AT)6	AAACAGTTGACAATCAAACAGGT	CGGAGGAAACAAGGCAAATA	58.0	215	NA	NA	NA	NA	NA	NA	NA	38.1
CaGMS630	(AT)9	(AT)6	TTGATGCATGACGTAGCCTT	TGTCAAAACAAGACATATAATTCCA	59.0	230	NA	NA	NA	NA	NA	NA	NA	31.8
CaGMS631	(ATT)7	(ATT)5	GCCGTAGAGTTTTGTCTGGG	AGGTTTCGGTGGACCAATTT	60.0	280	NA	NA	NA	NA	NA	NA	NA	42.9
CaGMS634	(TAT)7	(TAT)5	TGGTGAAGGGAATATAAGAAAGAAA	TCCAACTCATCACGAGTCATCT	59.0	280	NA	NA	NA	NA	NA	NA	NA	33.3
CaGMS635	(TA)10	(TA)7	AAATCAATGGACCGCCTCTT	AAAAATTCACAACCACTTCGAT	61.0	203	NA	NA	NA	NA	NA	NA	NA	42.9
CaGMS636	(TA)10	(TA)7	TTCAGATTTTGAGTGTCCCG	TGTGAGTGAATGTTGTTGTTGG	59.0	254	NA	NA	NA	NA	NA	NA	NA	33.3
CaTMS1421	(TC)8	(TC)7	CACCATGCTTACTTGGCTGA	TCCCAGCAAACATACAACCA	59.9	187	NA	NA	NA	NA	NA	NA	NA	54.0
CaTMS1422	(TA)8	(TA)7	TGTGTCATGTCAAGGACCGT	TTATGGTGCGATGACATCCT	60.0	177	NA	NA	NA	NA	NA	NA	NA	54.0
CaTMS1423	(CT)7	(CT)6	AGAGAGGGGCGAAGCTATGT	AAACAAGACCAATAAATTCAGCAA	60.4	156	NA	NA	NA	NA	NA	NA	NA	54.0
CaTMS1424	(TA)8	(TA)7	TTtGCTTACAAGGATAGTTTtACCA	CATTGTTGTCAGTTGAAGCCA	58.5	132	NA	NA	NA	NA	NA	NA	NA	53.2
CaTMS1425	(GT)8	(GT)7	TGAGTCAAGAGATGCAAATTGTT	TAGCCCCACCTGCAGTTATT	58.9	271	NA	NA	NA	NA	NA	NA	NA	53.2
CaTMS1426	(TA)9	(TA)8	GAAAaTAACATTAGGTTTGAGACAAAA	AAAGATAGGTGAATTGTTGGATG	58.3	278	NA	NA	NA	NA	NA	NA	NA	53.3
CaTMS1427	(AT)7	(AT)6	GCCATGTCGTGATTTTGGAT	AGACCAACCCCTTGCAAAAA	60.7	159	NA	NA	NA	NA	NA	NA	NA	53.3
CaTMS1428	(AT)8	(AT)7	TTGCATGTCAATTTTCAAGGAG	AGACTCACATCATTTCCACGG	60.1	216	NA	NA	NA	NA	NA	NA	NA	50.0
CaTMS1502	(AT)8	(AT)7	GGCCGTCTTCAAAATTCAAA	TGGATCTGGCATCAATTCAA	60.1	173	NA	NA	NA	NA	NA	NA	NA	50.0
CaTMS1503	(AT)7	(AT)6	GCTTTTCTTCTTGCAACTGCT	TGGCAGTTTGGATTACCACA	58.9	241	NA	NA	NA	NA	NA	NA	NA	50.0
CaTMS1504	(AG)7	(AG)6	GTTATGCCGGTTCGATTTTT	TGCACATGTGGAAGTGACAT	58.9	229	NA	NA	NA	NA	NA	NA	NA	50.0
CaTMS1505	(TA)7	(TA)6	CAACTGTGTCACATTATGTTCGC	GGCACCATACATACATAGTCCAAA	60.5	248	NA	NA	NA	NA	NA	NA	NA	50.0
CaTMS1506	(AT)7	(AT)6	CAATAACCCGGATGGAGCTA	CGTGGAAGAATACGTGGGTT	59.9	149	NA	NA	NA	NA	NA	NA	NA	50.0
CaTMS1507	(AT)7	(AT)6	GGAAATGTTTGGCGAATGTC	CACGGTGCATCTCACTCATAA	60.3	276	NA	NA	NA	NA	NA	NA	NA	50.0
CaTMS1508	(GT)8	(GT)7	CACAATGATAAATAaTTTGGAAAAGA	AGTCAACGGCTTGCTTGAtT	57.8	168	NA	NA	NA	NA	NA	NA	NA	50.0
CaTMS589	(AGA)5	(AGA)6	ATCGCGTGAAGGAGAGAGAG	TACAACACCAACGACGCATT	60.0	141	NA	NA	NA	NA	NA	NA	NA	50.3
CaTMS832	(CAA)6	(CAA)8	CCATCACCACCAACCCTAAC	AGAAGAGAGGGCGGAGTAGG	60.0	215	NA	NA	NA	NA	NA	NA	NA	50.0
CaGMS1413	(CA)7	(TG)7	AATACGTTTGAGGGTTGCCA	TTTGTATTTGTACCAATCGCATTA	60.4	253	NA	NA	NA	NA	NA	NA	NA	NA
CaGMS1414	(TG)6ttg(T)10	(TG)6	AAAGCGCTGTAAAATGCACA	TGATTGTGTGAACTGCTTATGG	59.5	240	NA	NA	NA	NA	NA	NA	NA	NA
CaGMS1415	(TA)7(GA)6	(TA)7	TGGAATGAAAGCAACGAAGA	GGATTTCAGAGCATGCACAA	59.4	195	NA	NA	NA	NA	NA	NA	NA	NA
CaGMS1416	(TTA)5	(TA)6	CTCTTTATGAAAAATTGTGAGACATT	TCtTTTGCTAACAAACACAAATCC	57.2	238	NA	NA	NA	NA	NA	NA	NA	NA
CaGMS1417	(CA)7(TA)7	(CA)7A(AT)7	CTGGACGTCGGTACGGTAaT	GAAGCAGCACTGTGGGTCTA	59.9	205	NA	NA	NA	NA	NA	NA	NA	NA
CaGMS1418	(TTA)6	(TG)8a(GT)6	CAGAAAATTGAAGGAAAAATTCCA	TTTCTCCTCTTTGGCACGTT	60.6	129	NA	NA	NA	NA	NA	NA	NA	NA
CaGMS1419	(AT)9(AC)6	(AT)9	AAAACAAGAAAGGGAAGGAAAA	ATATGCACCAACTTGAgGGG	58.4	271	NA	NA	NA	NA	NA	NA	NA	NA
CaGMS1420	(AGA)5(A)10*	(AGA)5	AAGGTCGCAACAGCCTACAT	ATGCCGTATTAGGGTCGTGA	59.8	258	NA	NA	NA	NA	NA	NA	NA	NA
CaGMS1429	(AT)7	(AT)6	AGTtAAGGgACcGCCTGAGT	CCCTACCTTGTAACTTTGTATATTTCA	60.1	243	NA	NA	NA	NA	NA	NA	NA	NA
CaGMS1430	(AT)7	(AT)6	CCACAATGAAGGCGGTAAGT	TGAGTACTGATAGGATTTGGTTGTTT	60.0	237	NA	NA	NA	NA	NA	NA	NA	NA
CaGMS1431	(AG)10	(AG)9	AACGCATCAATGACCGTACA	GTCGAGAACCCGAGAGACAG	60.0	154	NA	NA	NA	NA	NA	NA	NA	NA
CaGMS1432	(AG)8	(AG)7	CGAACACTGATCCGGAAACT	TCAGAAAGGAAAGAACACTGAAA	60.1	235	NA	NA	NA	NA	NA	NA	NA	NA
CaGMS1433	(TA)7	(TA)6	TGGGTTTTGCATAGTCGGTT	TCAATAGTCTCGGCTTCCTCA	60.4	198	NA	NA	NA	NA	NA	NA	NA	NA
CaGMS1434	(AT)8	(AT)7	TCAACATGTTAGGATTTGTGTCA	tGGTGGTTCAATTTGTGTTTATG	58.1	241	NA	NA	NA	NA	NA	NA	NA	NA
CaGMS1435	(TA)10	(AAG)5	TCATACATGCTCTTCGGCAC	CCGTAGTATCAATTGAATGGAATG	59.8	276	NA	NA	NA	NA	NA	NA	NA	NA
CaGMS1436	(CT)14	(CT)13	CCATGATACGAGAAGTGAGGG	GGTTACCTTGCGAGAAGCAG	59.6	217	NA	NA	NA	NA	NA	NA	NA	NA
CaGMS1437	(TA)7	(TA)6	TGTAAGGATTACTGCTTGCAAAT	TGATGCCGTTAAGCATGAAA	58.0	257	NA	NA	NA	NA	NA	NA	NA	NA
CaGMS1438	(AT)8	(AT)7	TTGAAAAACCCTAAATTTCAACAA	CTTTGGATGCATAGCACGTC	59.0	223	NA	NA	NA	NA	NA	NA	NA	NA
CaGMS1439	(AT)7	(AT)6	TtAAAATGGTCCCTCCGTCC	GTCCTTGCGAGTTGGAGAAC	61.0	264	NA	NA	NA	NA	NA	NA	NA	NA
CaGMS1440	(TA)7	(TA)6	TAAAGTTGGACGTGTCTGCG	CGAGTGAATTAATTAGCAGTGGC	59.9	138	NA	NA	NA	NA	NA	NA	NA	NA
CaGMS1441	(AT)7	(AT)6	AGCAACAGCCTGCTCAAAGT	TGTGTACACCCTTGTGGTGG	60.2	262	NA	NA	NA	NA	NA	NA	NA	NA
CaGMS1442	(TA)7	(TA)6	TTTGGGGCCACACACTTAAT	TGATTGGTTGTGTTTTAGGTCG	60.2	165	NA	NA	NA	NA	NA	NA	NA	NA
CaGMS1443	(GA)7	(GA)6	TTGAGGGAATATTTGATGATGA	AAATGCTCCCTCCAACAATG	57.0	179	NA	NA	NA	NA	NA	NA	NA	NA
CaGMS1444	(AT)7	(AT)6	CACTAAATCTCACCGTTGACTAAGG	GCCTTCAAATGCAATGACTG	60.4	207	NA	NA	NA	NA	NA	NA	NA	NA
CaGMS1445	(TA)11	(TA)10	GGAAATGAGCTGAAATTCCTGT	TGGCCTTATTGATCTCCaCA	59.6	205	NA	NA	NA	NA	NA	NA	NA	NA
CaGMS1446	(AT)7	(AT)6	TCACTCACACAAAATACGATTGC	AAAAGGGTGCCACACAGAAG	60.1	145	NA	NA	NA	NA	NA	NA	NA	NA

*Markers identity	Microsatellite repeat-motifs in ICC4958	Microsatellite repeat-motifs in PI489777	Forward primer sequences (5'-3')	Reverse primer sequences (5'-3')	Actual annealing temperature (0C)	Size (bp) of alleles amplified	Linkage groups (LGs)	Genetic positions (cM)	Markers used for polymorphism survey	Polymorphism information content (PIC)	Number of alleles amplified	Markers used in Figures	Markers types	GC%
CaGMS1447	(AT)8	(AT)7	TCAGAAGTTGGACATGATACACG	CTGGTGATGTGTTGGATGCT	60.0	247	NA	NA	NA	NA	NA	NA	NA	NA
CaGMS1448	(AG)11	(AG)10	GATGGAAATCCGAAATGACG	TGTCTTATCCGCTGCTTGTG	60.3	242	NA	NA	NA	NA	NA	NA	NA	NA
CaGMS1449	(CA)7	(CA)6	TCCATCTGATCCACCAACAA	CCTCTTGGAAATGACCCTCA	59.9	178	NA	NA	NA	NA	NA	NA	NA	NA
CaGMS1450	(AG)8	(AG)7	GGTGGTTTTCACAGCAGCTT	GGCATAGCAGAGTCCCAGTC	60.3	148	NA	NA	NA	NA	NA	NA	NA	NA
CaGMS1451	(AC)7	(AC)6	CACCTTCAATTTCGTTATGACA	CAACGTGGTTTTACGTCATTT	57.7	254	NA	NA	NA	NA	NA	NA	NA	NA
CaGMS1452	(AG)7	(AG)6	AAACCGGTGGTGTTGAAAAG	AGACCAATTTGCCGTGGTAG	59.9	182	NA	NA	NA	NA	NA	NA	NA	NA
CaGMS1453	(TC)7	(TC)6	TTCTGCAGTTCCAAACATGG	TAGACTCCAAGGGAgGAGGG	59.7	262	NA	NA	NA	NA	NA	NA	NA	NA
CaGMS1454	(AT)10	(AT)9	ATGCACGAACGGGAACTAAA	GGATTTTCAAGGCATCATAACC	60.5	241	NA	NA	NA	NA	NA	NA	NA	NA
CaGMS1455	(AC)12	(AC)11	TGAAGGATTGCCAGGTATGTT	GACGTGTTGATGTGTCAATGC	59.4	260	NA	NA	NA	NA	NA	NA	NA	NA
CaGMS1456	(TA)9	(TA)8	ACCATGGgTCTTGGgTCATA	GACGCACCACTATCTGGGAT	60.1	277	NA	NA	NA	NA	NA	NA	NA	NA
CaGMS1457	(GA)14	(GA)13	TtCATCATGTCATACCACGTCA	TTTTGTCCGTCCAATGTCAA	59.8	259	NA	NA	NA	NA	NA	NA	NA	NA
CaGMS1458	(AT)9	(AT)8	AAATATTtCAACTCCATGCATCTAA	TGCAATGAATGGAATCTTGG	58.2	153	NA	NA	NA	NA	NA	NA	NA	NA
CaGMS1459	(TA)11	(TA)10	TTTGCCATTCAAGAGGATCA	GATTCAGTTGGTATGGTTTTCG	59.2	229	NA	NA	NA	NA	NA	NA	NA	NA
CaGMS1460	(AT)10	(AT)9	GGGCATAAATGCCAAGAAAA	CAAAAGCTGTCTCTTATGTGCG	59.9	136	NA	NA	NA	NA	NA	NA	NA	NA
CaGMS1461	(GA)7	(GA)6	TCACTTGTTGCGACCAACTAAC	TCTTAAAATTATGAAGtTAAAATTGCC	60.2	258	NA	NA	NA	NA	NA	NA	NA	NA
CaGMS1462	(CA)9	(CA)8	GCCGAGTTCCCAACAATTAG	TGATCGGAATGGATAAGGGA	59.6	246	NA	NA	NA	NA	NA	NA	NA	NA
CaGMS1463	(TA)8	(TA)7	GGCAAATTTCAATTCATTCCA	TTGAGAACTCTGTTGGTATAGTTTCAG	59.8	261	NA	NA	NA	NA	NA	NA	NA	NA
CaGMS1464	(TA)10	(TA)9	GCAAACTCTTAGTTAAGTCTTTATCCA	CGGTGTGATATGATGTGGGA	58.0	142	NA	NA	NA	NA	NA	NA	NA	NA
CaGMS1465	(TA)7	(TA)6	CAAATTCTCGATGCTCCATGT	TCACATTCATGAGAAAAATCCG	60.1	268	NA	NA	NA	NA	NA	NA	NA	NA
CaGMS1466	(TA)8	(TA)7	CGGGCTACCTAGAATCCCTC	TTTCATGTGGTTGCATCCTAAG	60.1	207	NA	NA	NA	NA	NA	NA	NA	NA
CaGMS1467	(TA)7	(TA)6	CGAACCTAAAACTAACAAATCCTTT	CCACGTGCAAGAGAAATTGA	58.4	213	NA	NA	NA	NA	NA	NA	NA	NA
CaGMS1468	(CT)9	(CT)8	CGTCTACCTTCTTCCAAGCG	GAGAGGCGTCGATGAGAAAG	60.0	188	NA	NA	NA	NA	NA	NA	NA	NA
CaGMS1469	(AT)8	(AT)7	AGTATTTTgTTAGGGgCGGG	TCTGCCTGTACTCTCAGCTTTG	60.2	205	NA	NA	NA	NA	NA	NA	NA	NA
CaGMS1470	(TA)8	(TA)7	TGTTCTATGCATTTtGGTATTTtG	tTCAATTTTAcCCCTATTAACACAA	58.2	260	NA	NA	NA	NA	NA	NA	NA	NA
CaGMS1471	(CA)7	(CA)6	GTGGCCATCTATTTGCCATT	AAAATGTGTATGCCCCTTGC	59.8	228	NA	NA	NA	NA	NA	NA	NA	NA
CaGMS1472	(TA)9	(TA)8	TTACAATCAATTAAAGATATTTGCTCA	GGAAGTGCTCGAATCTGAGG	57.7	218	NA	NA	NA	NA	NA	NA	NA	NA
CaGMS1473	(TA)7	(TA)6	GCCACAAAATCAGACTGGCT	TAAGGAGAGCCAAAGTGGGA	60.3	114	NA	NA	NA	NA	NA	NA	NA	NA
CaGMS1474	(AT)9	(AT)8	CCAATGTGGCATTGATAATAGG	TCACGCATGTGAAAAGTGGT	59.2	222	NA	NA	NA	NA	NA	NA	NA	NA
CaGMS1475	(GT)8	(GT)7	CCTTAGGCCATCTGTTTGAA	CACTGGTTgGTTGGTCACTG	57.8	194	NA	NA	NA	NA	NA	NA	NA	NA
CaGMS1476	(AT)7	(AT)6	GAAAAACTCCTTTCATCTGTTATGC	ACCACATGTTTCgTGGTTGA	59.6	178	NA	NA	NA	NA	NA	NA	NA	NA
CaGMS1477	(AC)8	(AC)7	AAaCCAACATTGAGAGTGGGA	CTGAGCCCAcCATCTCTTTC	59.4	262	NA	NA	NA	NA	NA	NA	NA	NA
CaGMS1478	(AT)8	(AT)7	TGTTGCGACCAAAAATGGTA	GCAAGAGAACAGCAGCAACA	60.0	179	NA	NA	NA	NA	NA	NA	NA	NA
CaGMS1479	(GA)7	(GA)6	CCATTGGTGGAAAAGCAGTT	TTGtTGTTTGGATTTGATTTGC	60.0	226	NA	NA	NA	NA	NA	NA	NA	NA
CaGMS1480	(AG)7	(AG)6	TTTGCGATCCAGCAAGTATG	TGTTACTGCTTTTTGCGTGG	59.8	170	NA	NA	NA	NA	NA	NA	NA	NA
CaGMS1481	(TA)11	(TA)10	TGTGGTCACTTTATGGTATCTGA	TGAGCTCTTTTATTTAAGATTTTGC	57.2	258	NA	NA	NA	NA	NA	NA	NA	NA
CaGMS1482	(AC)7	(AC)6	TTTAAACTCGCAATCCGACa	TTTGTGTCATTTTAAGTCTCTACGA	59.3	233	NA	NA	NA	NA	NA	NA	NA	NA
CaGMS1483	(CT)11	(CT)10	TCAAAAGTGGCAATCTGCTG	aAATCATCAATCACCCTcGG	60.0	199	NA	NA	NA	NA	NA	NA	NA	NA
CaGMS1484	(TG)10	(TG)9	CATAAaTGCATGCGTCTAATGAA	AACAGATTCGTTGTTTGTGCC	60.0	256	NA	NA	NA	NA	NA	NA	NA	NA
CaGMS1485	(GA)8	(GA)7	CCTGTGGCTTTTGAATCCTT	ACTCCATTGCTGCTGCTTCT	59.2	242	NA	NA	NA	NA	NA	NA	NA	NA
CaGMS1486	(TA)7	(TA)6	CCTTCCcTTGGAATGCCTAT	TCTTCtTTTAAACCCTTATGAAAGTTG	60.3	235	NA	NA	NA	NA	NA	NA	NA	NA
CaGMS1487	(TC)10	(TC)9	GCAATACGTAGAAGCTTATTTTGAA	CCATGGAGAAACTTGCCCTA	58.3	236	NA	NA	NA	NA	NA	NA	NA	NA
CaGMS1488	(TA)8	(TA)7	GATTTtCTTTTGACCGGCCT	TGTGGtTTTTGTCCTGAATCC	60.4	101	NA	NA	NA	NA	NA	NA	NA	NA
CaGMS1489	(AT)9	(AT)8	GAGGTTTTCGGCATTCAAAA	CCCAAGATTCCTCtTCCACa	60.1	231	NA	NA	NA	NA	NA	NA	NA	NA
CaGMS1490	(GA)7	(GA)6	CCcTATCACcATGAAGCAAAA	GGCCTTTTGAGGTTTTCATCT	59.9	142	NA	NA	NA	NA	NA	NA	NA	NA
CaGMS1491	(AT)11	(CA)7	aGAAACATTATGAACACAACAATGA	CAGTTTGGCAAAAGTTAATTTCC	57.7	280	NA	NA	NA	NA	NA	NA	NA	NA
CaGMS1492	(TA)7	(TA)6	TCAAAATCGACCAATGTTGTG	TTGCAAGGATGTGATaACaaAAA	59.4	271	NA	NA	NA	NA	NA	NA	NA	NA
CaGMS1493	(TA)8	(TA)7	TTGGAATAAAAACCTCACCAAAA	TGACCATCCAACCTTACATGA	59.8	229	NA	NA	NA	NA	NA	NA	NA	NA
CaGMS1494	(TA)10	(TA)9	GTCGGTAAGTTCGGCAGGTA	TGTGTCACATGTGGAGGGAG	60.1	119	NA	NA	NA	NA	NA	NA	NA	NA
CaGMS1495	(TA)8	(TA)7	GGAAGGTGATTGGTGAAACG	CCCTTACATCTACAATCTCCAGTG	60.4	262	NA	NA	NA	NA	NA	NA	NA	NA
CaGMS1496	(GA)11	(GA)10	GAGGTGATGACCCTCCATTG	CGATTCACACCTTGTGTTCA	60.3	251	NA	NA	NA	NA	NA	NA	NA	NA
CaGMS1497	(TC)9	(TC)8	TTGCACCAAACCAGTAACCA	GAGCCAAAAATCTCCCATGA	60.0	263	NA	NA	NA	NA	NA	NA	NA	NA
CaGMS1498	(AT)10	(AT)9	CGAATTCTCCACAGGGATTC	TGGCTCATTTGATTTCACACA	59.5	251	NA	NA	NA	NA	NA	NA	NA	NA
CaGMS1499	(AT)9	(AT)8	TCACCCCTTTTCATCTCCAC	TGTATTAAGAACGTGTCAGAGAGTG	59.9	160	NA	NA	NA	NA	NA	NA	NA	NA

*Markers identity	Microsatellite repeat-motifs in ICC4958	Microsatellite repeat-motifs in PI489777	Forward primer sequences (5'-3')	Reverse primer sequences (5'-3')	Actual annealing temperature (0C)	Size (bp) of alleles amplified	Linkage groups (LGs)	Genetic positions (cM)	Markers used for polymorphism survey	Polymorphism information content (PIC)	Number of alleles amplified	Markers used in Figures	Markers types	GC%
CaGMS1500	(TC)10	(TC)9	TCtCCCTGCTCAATTAGCGT	GGTGGTTGTGGAGAAGTTTTG	60.0	246	NA	NA	NA	NA	NA	NA	NA	NA
CaGMS1509	(TA)8	(TA)7	CCTACCAATCTCAATCGCAT	TTAGGGGGTgGGAAAAgaAG	57.6	258	NA	NA	NA	NA	NA	NA	NA	NA
CaGMS1510	(AT)11	(AT)10	GGACCCGCAAGTCAAAAATA	CTTTTCCTGCCCCTTTCTCt	59.9	223	NA	NA	NA	NA	NA	NA	NA	NA
CaGMS1511	(AT)7	(AT)6	TGGCTCACAGGTCTCCTTCT	TGATGGAGCTTTTCATTTGC	60.0	172	NA	NA	NA	NA	NA	NA	NA	NA
CaGMS1512	(TA)7	(TA)6	GATGGCGGAAACGATGTC	TTTCAAAACCCGAATCAAAAA	60.0	224	NA	NA	NA	NA	NA	NA	NA	NA
CaGMS1513	(TA)7	(TA)6	GTTGCTTTGAACGGATGGAT	TGGTGCATGTAAAATTAATTAAAAGA	59.9	254	NA	NA	NA	NA	NA	NA	NA	NA
CaGMS1514	(AT)7	(AT)6	GGGACCACAGATGTAATTTAGCC	TTGCAGTTTGAACCAGAATCA	60.9	242	NA	NA	NA	NA	NA	NA	NA	NA
CaGMS1515	(AG)10	(AG)9	TGCAAATGCAAATTGAAACA	GCTCTCCCTAAACAAGCAGG	58.7	214	NA	NA	NA	NA	NA	NA	NA	NA
CaGMS1516	(GA)8	(GA)7	GGAGTTGGAGAGAGGCAATG	GGTCCCAACCACTCCCTTAT	59.8	252	NA	NA	NA	NA	NA	NA	NA	NA
CaGMS1517	(AT)10	(AT)9	TTAAACATTGCCTCGCTGTG	CCCAAAAAGCAAAACCTTCA	59.9	211	NA	NA	NA	NA	NA	NA	NA	NA
CaGMS1518	(AT)8	(AT)7	GGCAAAATGTGTTGTGACCA	CCGAAAAAGTCACCATCTGA	60.4	133	NA	NA	NA	NA	NA	NA	NA	NA
CaGMS1519	(TG)8	(TG)7	AAACAGACGTAGGTGTGCCC	ACCTCATCACTATCACCGCC	60.0	260	NA	NA	NA	NA	NA	NA	NA	NA
CaGMS1520	(TA)8	(TA)7	TGACAAGGTATGGCTGCAAA	CCCAAAATGTAACTGACCAAGG	60.3	135	NA	NA	NA	NA	NA	NA	NA	NA
CaGMS1521	(AT)7	(AT)6	GGTCTTTTATGAACCTTGTGATTTG	CAAGTGTGCAAGTCACGGTC	60.2	235	NA	NA	NA	NA	NA	NA	NA	NA
CaGMS1522	(AG)9	(AG)8	TGTTTGTTGGAGAGTGGGTG	TGAATGTGTCTTGGCATGGT	59.6	103	NA	NA	NA	NA	NA	NA	NA	NA
CaGMS1523	(GA)7	(GA)6	TCTGATGCTTGTCGGTTCTG	TTCGTGAATTTGTGGTGAGC	60.0	123	NA	NA	NA	NA	NA	NA	NA	NA
CaGMS1524	(TA)8	(TA)7	TGCTTCCACAATCATGAGAAA	tTcCACTCCACttTTaAACaACaA	59.3	257	NA	NA	NA	NA	NA	NA	NA	NA
CaGMS1525	(CT)12	(CT)11	CCATACCCCTAACGACCCTT	TGTGTGtCTTCCCTTTAtGACC	60.1	263	NA	NA	NA	NA	NA	NA	NA	NA
CaGMS1526	(AG)8	(AG)7	CTCAGCCTTCTTCAGCTTGC	GAAGGCTGTCTTGGGTGAAG	60.4	276	NA	NA	NA	NA	NA	NA	NA	NA
CaGMS1527	(GA)7	(GA)6	CAAGCACATCAAATAGTGGGA	TGAATCATTATCCCTCCTCCA	58.6	113	NA	NA	NA	NA	NA	NA	NA	NA
CaGMS1528	(TC)14	(TC)13	TCAAGGCTCAAAGAAAGGTGA	TGTAGGTGGtTTCTTGTGTGTG	60.0	195	NA	NA	NA	NA	NA	NA	NA	NA
CaGMS1529	(AT)7	(AT)6	CAACGTTACAGAAAGTCAAAGGT	CGAAATACCTGTATCCATTGCC	57.6	247	NA	NA	NA	NA	NA	NA	NA	NA
CaGMS1530	(TA)7	(TA)6	GTAGAATCGCCATCCACCAT	AGGGAGTGTGGGTAGCAAAA	59.8	150	NA	NA	NA	NA	NA	NA	NA	NA
CaGMS1531	(TA)7	(TA)6	GACACAAGCTTGCCTTCCAT	TCAGTCAAAAACAAAAAGATGTTGA	60.3	266	NA	NA	NA	NA	NA	NA	NA	NA
CaGMS1532	(TA)7	(TA)6	CAGCCCCCAATACTCCTACA	CGTCCTACATTGTTTGGAACTG	59.9	189	NA	NA	NA	NA	NA	NA	NA	NA
CaGMS1533	(TA)7	(TA)6	AGTTGATTGATCAAATTTTCCG	GAGAGCTAAATTGTTTTTCTGTTGA	58.1	250	NA	NA	NA	NA	NA	NA	NA	NA
CaGMS1534	(AT)8	(AT)7	TTTCTCAAAAATATGGTTGATTATCG	GGGGAGCATGAATGAGACAT	59.7	210	NA	NA	NA	NA	NA	NA	NA	NA
CaGMS1535	(AC)7	(AC)6	TTTCAAAATGAGTTATGGAATTTCTTT	TTTATGTTCGAAAGGGGTCG	59.7	256	NA	NA	NA	NA	NA	NA	NA	NA
CaGMS1536	(TA)7	(TA)6	TTTTtGCTTTGCTTTGATTTGA	TGGATGTAGAAATATAAAAATTGGGG	59.9	270	NA	NA	NA	NA	NA	NA	NA	NA
CaGMS1537	(AT)8	(AT)7	AAACAAATCGCCTGAAAAGG	GTCATGTGCGAGTTTGCATC	59.2	271	NA	NA	NA	NA	NA	NA	NA	NA
CaGMS1538	(AT)11	(AT)10	AAaaGaTAATGTAGAAGACTCCATGTG	GATTGAGCAACGGTGGAACT	58.4	230	NA	NA	NA	NA	NA	NA	NA	NA
CaGMS1539	(AT)11	(AT)10	GATGAGGGTTTCCAAGCTCA	ACACAAGTTTGGCATCTCCC	60.2	258	NA	NA	NA	NA	NA	NA	NA	NA
CaGMS1540	(TTA)15	(TTA)6	TGCCATTAATTGTTTtGGGAT	AAAATATAAATCGGCCCAACA	59.2	241	NA	NA	NA	NA	NA	NA	NA	NA
CaGMS1541	(AT)9	(AT)8	ACTTCTTGATAAGGATAGCAATCTAAC	CCAAAAGACTCACACTTAAGCC	57.1	167	NA	NA	NA	NA	NA	NA	NA	NA
CaGMS1542	(AG)7	(AG)6	TACAATTTGTTGACACCGGC	CTCACAGAGGGGTCACCAGT	59.4	170	NA	NA	NA	NA	NA	NA	NA	NA
CaGMS1543	(AT)9	(AT)8	CTTGTGACGGAAGGTAGATCG	TCAACATTGGAATTTGCCTG	59.7	206	NA	NA	NA	NA	NA	NA	NA	NA
CaGMS1544	(AT)7	(AT)6	CCCCTTCCTTGTAAATCGAC	ACACTTTCAATGAATCACAATCA	58.5	151	NA	NA	NA	NA	NA	NA	NA	NA
CaGMS1545	(TC)12	(TC)11	TGGTGCTGATCTCTCAAACG	TCCAAATCGGTGAGAAAACC	60.0	258	NA	NA	NA	NA	NA	NA	NA	NA
CaGMS1546	(AG)10	(AG)9	ATGGGAATTGACCTGGGTTT	TGCATGCACTCACACACACT	60.4	101	NA	NA	NA	NA	NA	NA	NA	NA
CaGMS1547	(TA)8	(TA)7	TGTCACATCATTCACTCCTCAA	TTTTGTGTTGAATTGTTTAGGTCTTT	59.1	280	NA	NA	NA	NA	NA	NA	NA	NA
CaGMS1548	(AT)7	(AT)6	AaTTTGTTCAAAATTtATCTAGACGAT	TGCTCTTTCCAGCTTGTCCT	57.2	253	NA	NA	NA	NA	NA	NA	NA	NA
CaGMS1549	(TTA)14	(TTA)5	AAGATTTAAACGTGATCCAAGCA	GGACGTGACATGATTAAGAGCA	60.0	256	NA	NA	NA	NA	NA	NA	NA	NA
CaGMS1550	(TA)7	(TA)6	CACGTGGGGGAATCAATAAT	CCTAAAACTTTCCAGTTGCATCA	59.5	229	NA	NA	NA	NA	NA	NA	NA	NA
CaGMS1551	(TC)8	(TC)7	TCATTTCATCATTTTCACGCA	TGCATATGTGAGTGTGCCTGt	60.1	189	NA	NA	NA	NA	NA	NA	NA	NA
CaGMS1552	(CA)7	(CA)6	CTGAAGAAAATGTTCCTGCACT	TGAGTTTTGATAGAATTCCGGTG	58.5	261	NA	NA	NA	NA	NA	NA	NA	NA
CaGMS1553	(CT)8	(CT)7	TGGGtgTTTCCATAGTGGGT	TAGCTTTCCCCTTGAATTGG	60.1	187	NA	NA	NA	NA	NA	NA	NA	NA
CaGMS1554	(GA)7	(GA)6	TTGGTTCGAAATGGAAAGAGA	GTGATCACCCCACAAATTCA	59.7	216	NA	NA	NA	NA	NA	NA	NA	NA
CaGMS1555	(TA)10	(TA)9	tGGGtGAAAAaCaTATACGACTG	TTGGAACGTGAAACAATGACA	58.9	275	NA	NA	NA	NA	NA	NA	NA	NA
CaGMS1556	(AG)7	(AG)6	TGCCATGGATTTCAATTGTC	CGGGAAGCTTGTTTGTTTGT	59.3	243	NA	NA	NA	NA	NA	NA	NA	NA
CaGMS1557	(TA)9	(TA)8	TCTCATTTTCCCAATGAGGC	GACAAAAGTTGCCAAGAGTTCA	60.0	162	NA	NA	NA	NA	NA	NA	NA	NA
CaGMS1558	(AG)7	(AG)6	TCAAGGCTGAAGGaGAAAACA	CaAGCACAAtTTAACTccCCC	60.0	229	NA	NA	NA	NA	NA	NA	NA	NA
CaGMS1559	(AT)7	(AT)6	CAAATCAGGAATTGAGGAATCA	AGCCCATACTTGGATTATTCAG	59.0	170	NA	NA	NA	NA	NA	NA	NA	NA
CaGMS1560	(TAA)16	(TAA)7	CCCAATCCACCTGGATGTAA	ACGGACCATGGTGTTCTTGT	60.6	277	NA	NA	NA	NA	NA	NA	NA	NA

*Markers identity	Microsatellite repeat-motifs in ICC4958	Microsatellite repeat-motifs in PI489777	Forward primer sequences (5'-3')	Reverse primer sequences (5'-3')	Actual annealing temperature (0C)	Size (bp) of alleles amplified	Linkage groups (LGs)	Genetic positions (cM)	Markers used for polymorphism survey	Polymorphism information content (PIC)	Number of alleles amplified	Markers used in Figures	Markers types	GC%
CaGMS1561	(AT)9	(AT)8	ATATTtCACATTGATCACATTTCTG	CCATAGCCAAAATTGCAGGT	57.2	138	NA	NA	NA	NA	NA	NA	NA	NA
CaGMS1562	(AT)8	(AT)7	TGTATCGGCATCCAAAATGA	ATGCTTTGGGTGCTTGTTTC	59.9	225	NA	NA	NA	NA	NA	NA	NA	NA
CaGMS1563	(GT)9	(GT)8	AGTGATGGAATGAGGCGTTC	TCCCATAAGCAGCTAAAAGCA	60.1	268	NA	NA	NA	NA	NA	NA	NA	NA
CaGMS1564	(AG)7	(AG)6	TGAAGAACATGCGAAACCTG	CCTTCTCAACCTCCACTTCG	59.8	266	NA	NA	NA	NA	NA	NA	NA	NA
CaGMS1565	(AT)8	(AT)7	TGAGTTGAAAGTGGGGATGG	AATTTCAATGATGAGAAAAACTAAGAT	60.9	118	NA	NA	NA	NA	NA	NA	NA	NA
CaGMS1566	(AG)7	(AG)6	TGAAAGCAAGGACTAGCGAAA	TCCATCGATTCCTATGGCTC	60.1	184	NA	NA	NA	NA	NA	NA	NA	NA
CaGMS1567	(TA)9	(TA)8	CGAGAGCTCTGATACCATCTCA	CACCCCATTTGCTTTATTGC	59.6	269	NA	NA	NA	NA	NA	NA	NA	NA
CaGMS1568	(AT)8	(AT)7	TGTGTTTCAATCGTTTAAATCCT	TTTCTGAGTTTTAATTGTTGAGGAAA	57.8	223	NA	NA	NA	NA	NA	NA	NA	NA
CaGMS1569	(TAA)22	(TAA)13	TCAAAATGAAGGATCAAAACTGT	AAAATGGTGtCACAGACGCA	57.8	204	NA	NA	NA	NA	NA	NA	NA	NA
CaGMS1570	(AT)7	(AG)9	AGATTGGGTTGGCGATTTC	TTTGCACTAGTCGCATTTCA	59.9	119	NA	NA	NA	NA	NA	NA	NA	NA
CaGMS1571	(TA)7	(TA)6	CGCCTAGGGCACCAAATATC	TCATCCTAGTGATAAACGGCA	61.7	101	NA	NA	NA	NA	NA	NA	NA	NA
CaGMS1572	(TA)7	(TA)6	AGCGCGAGTTGATAGTGTGT	TTTTTCTTGGCTGATGTGATT	58.6	119	NA	NA	NA	NA	NA	NA	NA	NA
CaGMS1573	(GA)8	(GA)7	ACGAAATTGGGAGAAAaGGG	ACATCGTTTTCCTTTGTCCG	60.3	253	NA	NA	NA	NA	NA	NA	NA	NA
CaGMS1574	(AC)7	(AC)6	ACATGCCCAAACCACCTAAG	TTGAAGGAGCTAGGGTGGAA	59.9	159	NA	NA	NA	NA	NA	NA	NA	NA
CaGMS1575	(TTA)8	(AT)7	TGGATGGATTGGTTGAATGA	TGAAGGAGGGACCAAAACtG	59.7	195	NA	NA	NA	NA	NA	NA	NA	NA
CaGMS1576	(AT)9	(AT)8	CCAGCTAGGACCAAAGCAAT	TTTGTTCCTGAGATATTTTCACAA	59.3	280	NA	NA	NA	NA	NA	NA	NA	NA
CaGMS1577	(TA)7	(TA)6	CAACTATATTTGAAACTTCCTGAAAAA	TTCAAACACTCTTTTGTTTTGTTGA	58.4	192	NA	NA	NA	NA	NA	NA	NA	NA
CaGMS1578	(AC)7	(AC)6	ACCTGAATCCTTACATTAGATCTTG	GATGCACCGATGAACTTGTG	57.1	102	NA	NA	NA	NA	NA	NA	NA	NA
CaGMS1579	(TA)7	(TA)6	ATGAGCGCAAGTGTCAAACA	AAACCACTTAACCTCGCCAA	60.5	235	NA	NA	NA	NA	NA	NA	NA	NA
CaGMS1580	(TA)8	(TA)7	AGAATGAACCATTGGCTtGG	ATCGTGAGTTTGAGGGGAGA	59.9	158	NA	NA	NA	NA	NA	NA	NA	NA
CaGMS1581	(TC)14	(TC)13	TCGCTCTCTCGCTCTCTTTC	TCAATGATGGTGAAATTTGGAA	60.1	157	NA	NA	NA	NA	NA	NA	NA	NA
CaGMS1582	(TTA)17	(TTA)8	TGgATTTTgtCTGCACACCT	ACTTGCACGTCATCCAAACA	59.1	270	NA	NA	NA	NA	NA	NA	NA	NA
CaGMS1583	(TG)7	(TG)6	TTAACcAAACGAGTGGCACA	CTCGGTTGATCATCCCTTGT	60.1	169	NA	NA	NA	NA	NA	NA	NA	NA
CaGMS1584	(AT)10	(AT)9	AAAGTGCATGCAGTGTACCG	GAGTCCTTGAGTTGGTGCAA	59.8	149	NA	NA	NA	NA	NA	NA	NA	NA
CaGMS1585	(T)10	(TA)8	TTTtGTTCTtgTTGTTGTCaTCG	GCCCaTGTAAAAAtAATTGGCT	60.1	274	NA	NA	NA	NA	NA	NA	NA	NA
CaGMS1586	(TA)10	(TC)12	TCATGTTTTtGGTCATGGAGA	TAACATCAGGGGAGGACCAG	59.0	138	NA	NA	NA	NA	NA	NA	NA	NA
CaGMS1587	(TA)8	(AT)6	TTTAACACGCTTGAATACCGA	CTCGTTTGATTTCAGCACCA	58.4	248	NA	NA	NA	NA	NA	NA	NA	NA
CaGMS1588	(AT)7	(ATA)5	ACGGAAaCAAACGGACGAT	TGGTGTTGGAGTCATGCTTT	60.4	210	NA	NA	NA	NA	NA	NA	NA	NA
CaGMS1589	(AC)8	(TA)7	TGCAAACCGTTTTATTtGTCC	TGTGCAAGAGAGTGGTTGaAA	59.9	244	NA	NA	NA	NA	NA	NA	NA	NA
CaGMS1590	(ATA)22	(ATA)13	TCTTTTTGTCTAAATGCAATGCTC	GTCGAGACGAAAGCCAACTC	59.8	216	NA	NA	NA	NA	NA	NA	NA	NA
CaGMS1591	(AT)15	(TGT)6	TCAAAAACCCCAATTGCCTA	CACAAAGGCCACTTCCAtTT	60.3	113	NA	NA	NA	NA	NA	NA	NA	NA
CaGMS1592	(AT)13	(AT)7	GTGTATGAAACTGGGGgAGG	CATGCaCaAAAATTatTgCtctTTA	59.3	190	NA	NA	NA	NA	NA	NA	NA	NA
CaGMS1593	(TC)8	(TA)6	ACAAAAGGGTTCGAACGTGA	TACCTTTGGAATGGGGAACA	60.5	188	NA	NA	NA	NA	NA	NA	NA	NA
CaGMS1594	(TA)7	(TA)6	TTGATCAAAATTGGCATTCA	TTTTGGTTGTGTGGGATTGA	57.5	269	NA	NA	NA	NA	NA	NA	NA	NA
CaGMS1595	(TA)10	(TA)9	CGTCACTTTGGAAAGTCAACAA	TTCGTTGAAAATGTTACCGTTG	60.2	251	NA	NA	NA	NA	NA	NA	NA	NA
CaGMS1596	(CT)7	(CT)6	TGCCTGAGGCGAATAAGTTT	TACGAATCCGAGTGGTGACA	59.8	188	NA	NA	NA	NA	NA	NA	NA	NA
CaGMS1597	(AT)7	(AT)6	AATCCAACCTGTTTTCTCATTATT	TGCAACATCCATGTATTCCG	57.3	254	NA	NA	NA	NA	NA	NA	NA	NA
CaGMS1598	(TA)8	(TA)7	AGGAGGGCATGGTGAGTATG	GGTCTTCCTTATTAATGCCGC	60.0	273	NA	NA	NA	NA	NA	NA	NA	NA
CaGMS1599	(A)10	(TTA)5	TGTGTCATTGGTGTTGTGGA	CTACtTTTTGAAACGcCCCA	59.4	243	NA	NA	NA	NA	NA	NA	NA	NA
CaGMS1600	(AT)10	(ATT)6	TGAGCTGAATGGTCGAGTTG	AGTTGGCGTATTCCAACAAA	60.0	132	NA	NA	NA	NA	NA	NA	NA	NA
CaGMS1601	(TTA)20	(TTA)11	GAGGCTCTGGATGCAGTGAT	GGGTGAAGATTTACGGAAGC	60.4	248	NA	NA	NA	NA	NA	NA	NA	NA
CaGMS1602	(TA)9	(CTT)6	GCTTTGAACATTTTGAGGCA	CACTTGAGATCAATTTTCACGG	58.9	102	NA	NA	NA	NA	NA	NA	NA	NA
CaGMS1603	(TA)8	(TA)7	TCCGATTAAATAAAAACTCCATCA	CAAGTGTTCAAAGAcCCCGT	59.0	139	NA	NA	NA	NA	NA	NA	NA	NA
CaGMS1604	(AT)9	(AT)8	TTGGTTTCCTTTGTGTTTTGAA	GGCGACGAGCACATTATTTC	59.5	232	NA	NA	NA	NA	NA	NA	NA	NA
CaGMS1605	(AT)7	(AT)6	ATGAATGACTTCCGCCAAAC	GGGCAGACACTCTCTCGAAC	59.9	265	NA	NA	NA	NA	NA	NA	NA	NA
CaGMS1606	(AT)8	(AT)7	TTGTCGCAACTCAATTGACAG	TGTATTTGTGTTGAATTGTTTATGCT	59.9	238	NA	NA	NA	NA	NA	NA	NA	NA
CaGMS1607	(TA)8	(TA)7	TGATGCGTCCAATGTTCAAT	CAAAAGCGGCAAAAATTAGG	59.9	240	NA	NA	NA	NA	NA	NA	NA	NA
CaGMS1608	(GT)7	(GT)6	CAGTGGCATTGAGACATTGAC	TCGCTCCAATTTTCATTGTG	59.2	277	NA	NA	NA	NA	NA	NA	NA	NA
CaGMS1609	(TTA)15	(TTA)6	CACTCGGTTTTAAtCTCCATCA	TGTGTGTGAGTTTGATCTGGTG	59.1	232	NA	NA	NA	NA	NA	NA	NA	NA
CaGMS1610	(TA)8	(TA)7	CATGTTCATGTATGTTGGTGCT	TGTCTGAAATTAGCAAGAAGGGA	58.4	221	NA	NA	NA	NA	NA	NA	NA	NA
CaGMS1611	(TG)7	(TG)6	CCACACTAAAACAACACTGCAA	CCATAGAACAATGCATAAACACC	58.8	219	NA	NA	NA	NA	NA	NA	NA	NA
CaGMS1612	(AT)7	(AT)6	GGAGGAAGAAAGGTTCTCGC	AATGCGTTACGGGTGAAATG	60.3	276	NA	NA	NA	NA	NA	NA	NA	NA
CaGMS1613	(GA)9	(GA)8	TTACAAAACaCGCAAGTGGG	CAGAAACACGCAGGAACTCA	59.6	259	NA	NA	NA	NA	NA	NA	NA	NA

*Markers identity	Microsatellite repeat-motifs in ICC4958	Microsatellite repeat-motifs in PI489777	Forward primer sequences (5'-3')	Reverse primer sequences (5'-3')	Actual annealing temperature (0C)	Size (bp) of alleles amplified	Linkage groups (LGs)	Genetic positions (cM)	Markers used for polymorphism survey	Polymorphism information content (PIC)	Number of alleles amplified	Markers used in Figures	Markers types	GC%
CaGMS1614	(CT)8	(CT)7	CGACCGATAGATTTGGCATT	TTGCAGCCTTGAAATCACAC	59.9	280	NA	NA	NA	NA	NA	NA	NA	NA
CaGMS1615	(AG)7	(AG)6	GAAGATGCTTGAACGCAACA	ACCAAACACACTCCTCTGCC	60.0	186	NA	NA	NA	NA	NA	NA	NA	NA
CaGMS1616	(AG)8	(AG)7	ATGTTGGTTTGATTCCAAAAA	CAAAACACATTTAAACCTTTCaAAAA	57.0	127	NA	NA	NA	NA	NA	NA	NA	NA
CaGMS1617	(AT)9	(AT)8	tCAAAACTCAACTTCTAAATACGCTC	TGTCGTATCACCGTCGACAT	59.5	266	NA	NA	NA	NA	NA	NA	NA	NA
CaGMS1618	(TG)8	(TG)7	AACTTGGTGGCTGAAAATGG	CAATCACTCAGCTCCCCTTC	60.0	173	NA	NA	NA	NA	NA	NA	NA	NA
CaGMS1619	(TA)7	(TA)6	CTTCAACTCATGATGTACCGGA	GAACTTCATTGGTGGGTGCT	60.0	193	NA	NA	NA	NA	NA	NA	NA	NA
CaGMS1620	(ATA)6	(AT)6	TGCATGCAGTCAAAAGTCAA	AGCTAGCATTCCCATGCACT	59.0	208	NA	NA	NA	NA	NA	NA	NA	NA
CaGMS1621	(AT)8	(AT)7	TGTGAGTGAAGTGATGTGCG	AAGAAGAAGAAGGGGTTCGC	59.4	177	NA	NA	NA	NA	NA	NA	NA	NA
CaGMS1622	(TA)9	(TA)8	TGATTGATTATTGAAACTCAAAAAGA	AAGGaAAAACTTGGAGGGACA	58.4	237	NA	NA	NA	NA	NA	NA	NA	NA
CaGMS1623	(TA)7	(TA)6	TTTCTTGCCTTCTTTTCATGC	AGCACTTGTGCCACACCATA	59.5	249	NA	NA	NA	NA	NA	NA	NA	NA
CaGMS1624	(TA)9	(TA)8	GGGGCATGGAGTAGTTGAAA	GACAGGTGGAATTGCTTGGT	59.9	253	NA	NA	NA	NA	NA	NA	NA	NA
CaGMS1625	(TA)9	(TA)8	CACTAACCTGAAGTTGGCTGAA	CCACCGCCTTATCCTAAACA	59.4	144	NA	NA	NA	NA	NA	NA	NA	NA
CaGMS1626	(TA)7	(TA)6	TTTCGTTCAACGGTTGTTTG	GCACTCCCAAATCCCAtTTT	59.6	220	NA	NA	NA	NA	NA	NA	NA	NA
CaGMS1627	(AT)7	(AT)6	TCCCATACTTTGCACTAGGATTT	TGGGGGAGTAAAGAATTTGAG	59.1	177	NA	NA	NA	NA	NA	NA	NA	NA
CaGMS1628	(TA)7	(TA)6	TCACCAAATTTGAATGCATGA	GTCGCAGTACGAAGATGcAA	59.9	124	NA	NA	NA	NA	NA	NA	NA	NA
CaGMS1629	(CA)9	(CA)8	CAACGTATCGACACACCCTG	GGAACACTGTGATTTCGGCT	60.0	187	NA	NA	NA	NA	NA	NA	NA	NA
CaGMS1630	(TA)9	(TA)8	GCCACGATTTATACCGCTTT	TGAATCCCAAAAGCATTGAA	59.1	163	NA	NA	NA	NA	NA	NA	NA	NA
CaGMS1631	(ATT)18	(ATT)9	TTGTTCGATTTCTATGGACAAA	aAgAATCaATAATCTGCAAAATGAA	57.3	258	NA	NA	NA	NA	NA	NA	NA	NA
CaGMS1632	(AT)8	(AT)7	TTAAAGAGATTCGCAGCGgT	GCAAGCATTTTGTGTAAGTGTGA	60.0	110	NA	NA	NA	NA	NA	NA	NA	NA
CaGMS1633	(AT)7	(AT)6	TGCAATCTCAACAATGGTAAAA	CACCTCCATTGAAGCTGTTG	58.2	258	NA	NA	NA	NA	NA	NA	NA	NA
CaGMS1634	(AT)8	(AT)7	TTTGATACACACCACACCACA	GTGATGCGATCTGAAGGTCA	58.3	127	NA	NA	NA	NA	NA	NA	NA	NA
CaGMS1635	(AT)7	(AT)6	TTGGAAGGGTTTATTTGGGTC	TCACACTCGTATATCTCGCAAAA	60.0	280	NA	NA	NA	NA	NA	NA	NA	NA
CaGMS1636	(TC)10	(TC)9	TGACCTCCTTGACCTTGGTT	GGGTTGTGTGCACTTGTACCT	59.5	218	NA	NA	NA	NA	NA	NA	NA	NA
CaGMS1637	(TC)8	(TC)7	CAAACAAAAGAATCGAGGGC	CAAATAAAGCGGTTCTTTCAGG	59.7	196	NA	NA	NA	NA	NA	NA	NA	NA
CaGMS1638	(AG)14	(AG)13	AAGTGGAGTCATAACGGGGT	GAGCGCAATTCTCTTCAAGG	58.4	220	NA	NA	NA	NA	NA	NA	NA	NA
CaGMS1639	(CT)8	(CT)7	TCTGCCCGTTCTTATACCCA	AGTGGCAAAGACGGAGAGTG	60.5	100	NA	NA	NA	NA	NA	NA	NA	NA
CaGMS1640	(AT)7	(AT)6	GCCACATCATTATTTGAGCG	TTCAAAATGTACTTATTTACCCTCCA	59.1	211	NA	NA	NA	NA	NA	NA	NA	NA
CaGMS1641	(AT)9	(AT)8	TTGCCACTTGGCCATACATA	CAAAGCATACGTACTTTGGCA	60.0	265	NA	NA	NA	NA	NA	NA	NA	NA
CaGMS1642	(AAT)17	(AAT)8	TGTtATTGGTTATCCCAACCG	GCTTTTGAATCAAGTGGTTATTTTC	59.6	237	NA	NA	NA	NA	NA	NA	NA	NA
CaGMS1643	(TA)7	(TA)6	AtCAAAGAACTCGGCAGGTG	TCCATAAGCGTGACTTTGACtT	60.3	115	NA	NA	NA	NA	NA	NA	NA	NA
CaGMS1644	(TG)8	(TG)7	CATAGGAGTTGTGTATTTCCAAGC	GTGCCAACAATCTTCCCATT	59.1	236	NA	NA	NA	NA	NA	NA	NA	NA
CaGMS1645	(AT)7	(AT)6	TGCAAAGTGGTGAGAGGAAA	GCTTGTCAAACCTTTGGGAG	59.4	239	NA	NA	NA	NA	NA	NA	NA	NA
CaGMS1646	(AT)7	(AT)6	CAGTGTAGCTACGAGGTATCGG	GAAATGTGAAGATGAGGTGATGA	59.0	218	NA	NA	NA	NA	NA	NA	NA	NA
CaGMS1647	(CA)7	(CA)6	CATGGCAACATGCCAATTAC	CGCaATCTTTGAGTTCGACA	59.8	184	NA	NA	NA	NA	NA	NA	NA	NA
CaGMS1648	(AT)9	(AT)8	CGTGTGTAATGGTACGTCGG	CGAGCGAAATGCATGTATGA	59.9	263	NA	NA	NA	NA	NA	NA	NA	NA
CaGMS1649	(TG)9	(TG)8	CAAAAAGCAAATGCCAACCT	ATGGGAAAGCCCAAAGAGAT	60.1	248	NA	NA	NA	NA	NA	NA	NA	NA
CaGMS1650	(AT)9	(AT)8	CATGGACTTGACAAAAATCCAA	TTCTTTGTCTGCGATACCTTCA	59.8	233	NA	NA	NA	NA	NA	NA	NA	NA
CaGMS1651	(AT)7	(AT)6	TTTCCAAAGTACAACGCATCA	TTTCCCCTTGGTGAAGAAAA	59.2	203	NA	NA	NA	NA	NA	NA	NA	NA
CaGMS1652	(AT)8	(AT)7	GTGTCAACAAACAAATCGGG	CCTTTGGGATGAAATTGACG	58.9	263	NA	NA	NA	NA	NA	NA	NA	NA
CaGMS1653	(GT)13	(GT)12	CCACAATTTTTGGGTTCATTT	AAGAGTTGTTGAGATGGAAGAAA	58.7	266	NA	NA	NA	NA	NA	NA	NA	NA
CaGMS1654	(AT)7	(AT)6	TTGAGTTGCAACGAGAGTGG	AAGAACGAGAATGGGTTTCG	60.0	250	NA	NA	NA	NA	NA	NA	NA	NA
CaGMS1655	(TG)10	(TG)9	GAACAAAATGAAACAAAACCACA	TGGCAAGTAAAGAATTCAGCC	58.9	217	NA	NA	NA	NA	NA	NA	NA	NA
CaGMS1656	(AT)7	(AT)6	GGGgCTCAATTTGTTTGTTT	GGCCAAGGTTAGGGTGAGAT	58.9	216	NA	NA	NA	NA	NA	NA	NA	NA
CaGMS1657	(TA)7	(TA)6	ATGTTCACAACCTCGCATGA	GTTGAAAATCATTCCCTCGC	60.1	190	NA	NA	NA	NA	NA	NA	NA	NA
CaGMS1658	(TA)8	(TA)7	ATTAATGAAGATCCGCGGTG	TTTGGATTTATGCGGATTCA	59.9	277	NA	NA	NA	NA	NA	NA	NA	NA
CaGMS1659	(TC)8	(TC)7	GTCCGAAAAGAAGGATGCAG	GAGAATGAGGAAGCAAACGC	59.8	185	NA	NA	NA	NA	NA	NA	NA	NA
CaGMS1660	(TA)10	(TA)9	CCATAGAGCCTCCCTTGTGA	CCGATCCCATTGTCCACTTA	60.2	270	NA	NA	NA	NA	NA	NA	NA	NA
CaGMS1661	(TA)7	(TA)6	TTCTTGCTCTTGCTGCTGAA	CTTTCCAATCAGGACCACCA	60.0	230	NA	NA	NA	NA	NA	NA	NA	NA
CaGMS1662	(AT)8	(AT)7	TGTGAGGTTGAGGTTTCTTGTTT	TTTCTTTGACATCAATGAGATGA	60.1	123	NA	NA	NA	NA	NA	NA	NA	NA
CaGMS1663	(TA)7	(TA)6	GTCGCCAGTGTGACTGAAAA	GATTTTAAGCGGCCAAACaA	59.9	259	NA	NA	NA	NA	NA	NA	NA	NA
CaGMS1664	(AT)8	(AT)7	TtCTTTTtCCCTCCAACCAA	ATGCCGTTTGGAATTACTCG	59.5	175	NA	NA	NA	NA	NA	NA	NA	NA
CaGMS1665	(AT)10	(AT)9	CCTTCTCTTTTCCCTCCACA	CGGTGAATCCTTTACGGAGA	59.3	146	NA	NA	NA	NA	NA	NA	NA	NA
CaGMS1666	(ATT)8	(AT)6	CAATTTCCTCCTATTTGTTTTGACA	CGAGGATGCGACCTTGtACT	60.6	243	NA	NA	NA	NA	NA	NA	NA	NA

*Markers identity	Microsatellite repeat-motifs in ICC4958	Microsatellite repeat-motifs in PI489777	Forward primer sequences (5'-3')	Reverse primer sequences (5'-3')	Actual annealing temperature (0C)	Size (bp) of alleles amplified	Linkage groups (LGs)	Genetic positions (cM)	Markers used for polymorphism survey	Polymorphism information content (PIC)	Number of alleles amplified	Markers used in Figures	Markers types	GC%
CaGMS1667	(TTA)11	(TTA)5	TGATTGATAAATTGACGAACTACAAA	AAAAATACGTGCCAGCCTAAA	59.1	208	NA	NA	NA	NA	NA	NA	NA	NA
CaGMS1668	(ATT)11	(ATT)5	TGAGTTGGACTCGTGAATCG	CGAAACCAAGGTAACGTGAAA	59.8	245	NA	NA	NA	NA	NA	NA	NA	NA
CaGMS1669	(AAT)11	(AAT)5	CCGATTGGAAACAAACCTGT	TTcCCCtTTTATTcTCCaAACa	59.8	164	NA	NA	NA	NA	NA	NA	NA	NA
CaGMS1670	(ATA)17	(ATA)11	TTGATGAAaCCGACCcAACT	TCCAAGGATGGCTGAtTTTC	60.4	126	NA	NA	NA	NA	NA	NA	NA	NA
CaGMS1671	(AAG)19	(AAG)13	GGGATGACCGCTATTGTGAA	CtTTCACCGTTCCTCCTCTG	60.9	259	NA	NA	NA	NA	NA	NA	NA	NA
CaGMS1672	(TTA)11	(TTA)5	CACGGAGAGAAACACTTGTTCA	CAACCCCAGACTTTTGAGGA	60.3	249	NA	NA	NA	NA	NA	NA	NA	NA
CaGMS1673	(AG)7	(AT)6	ATAGTGGACCCCACAATCCA	TGTGAGAAAATTAAGGAAGAGTTTGA	60.1	268	NA	NA	NA	NA	NA	NA	NA	NA
CaGMS1674	(TTA)10	(TTA)5	CTTAAAATCCCGACCTCACG	TTCCATTGGACTCGTTGTGA	59.6	145	NA	NA	NA	NA	NA	NA	NA	NA
CaGMS1675	(TTA)17	(TTA)12	CTCCGACTCATTGAATTGACA	TGTTTATGTATTTCAACATATTCAGCA	58.7	149	NA	NA	NA	NA	NA	NA	NA	NA
CaGMS1676	(TTA)12	(TTA)7	GACATTTCCAACCAAGCCAT	GGCTCACTCATGTTGACGTTT	59.8	275	NA	NA	NA	NA	NA	NA	NA	NA
CaGMS1677	(TAT)18	(TAT)13	TGTCAATTGTACTAAATCACATCAAA	TGGTTTCCATCAACAACAACA	57.8	185	NA	NA	NA	NA	NA	NA	NA	NA
CaGMS1678	(AAT)46	(AAT)9	TCGCTGAACAGTCGTGCTAA	TTTTAGTCATTGCAAAAATTaTTCATC	60.7	270	NA	NA	NA	NA	NA	NA	NA	NA
CaGMS1679	(AT)11	(AT)6	CCATGTCCATTTAATTGCTGA	AACCCCACACACACACACAC	58.5	233	NA	NA	NA	NA	NA	NA	NA	NA
CaGMS1680	(ATT)41	(ATT)5	AGTGGATGTAACCATGCATTC	TTGTTATTGTTAtTTTTGGGTGAAA	57.4	271	NA	NA	NA	NA	NA	NA	NA	NA
CaGMS1681	(TTA)9	(TTA)6	AAGTTGAGGAAGGGGATGGT	TAGTGGGCTTCGTATTTGGG	59.8	274	NA	NA	NA	NA	NA	NA	NA	NA
CaGMS1682	(TTC)8	(TTC)5	TTTCGTTTtCCACTGCTCTG	TCTTGTTCATCGCTTCATTCA	59.0	194	NA	NA	NA	NA	NA	NA	NA	NA
CaGMS1683	(AAG)10	(AAG)7	CCAGCCATGGTAATTTGGAC	TTCTACAACCGTTTTTCTTCTCTG	60.2	204	NA	NA	NA	NA	NA	NA	NA	NA
CaGMS1684	(CTA)8	(CTA)5	TGAGTTGACACGCAGAATGA	CCCCTATGGCACAAAATCTC	59.0	163	NA	NA	NA	NA	NA	NA	NA	NA
CaGMS1685	(AAT)8	(AAT)5	TGTGTGTGTTTTGTGGCCTT	CGTATCGAATTTTGTAAGCATTTG	60.0	248	NA	NA	NA	NA	NA	NA	NA	NA
CaGMS1686	(AT)9	(AT)6	AACTCGCAAGTTACGCGA	TTCAACATTTAGCATATCCATCG	58.2	239	NA	NA	NA	NA	NA	NA	NA	NA
CaGMS1687	(TTA)13	(TTA)10	CCATAATCTTACGTGGTTTGTCA	TGAAAATCTTCGTCATCTTTTTCTT	58.9	168	NA	NA	NA	NA	NA	NA	NA	NA
CaGMS1688	(AGG)10	(AGG)7	ACGCAAAGCCGAGTTCTAAA	CGTTCCCTTTCTCCTTCTCC	60.0	153	NA	NA	NA	NA	NA	NA	NA	NA
CaGMS1689	(TTA)9	(TTA)6	GCAAACATGTTGGGATAGCA	GAGACAAGTGTCACCACACGA	59.5	188	NA	NA	NA	NA	NA	NA	NA	NA
CaGMS1690	(ATA)8	(ATA)5	ATTTGTGGGATGACACCGTT	GTACCCCCACCGTAGGATCT	60.1	278	NA	NA	NA	NA	NA	NA	NA	NA
CaGMS1691	(AAT)9	(AAT)6	GGACGAAAAGGTGTCCACATA	TTTGAATTCGTGTGTTCGGA	59.8	168	NA	NA	NA	NA	NA	NA	NA	NA
CaGMS1692	(TCT)8	(TCT)5	TGCCAACTCAACAATCTCCA	CCAGTCCTCCAACTACTCCAA	60.2	114	NA	NA	NA	NA	NA	NA	NA	NA
CaGMS1693	(TTA)10	(TTA)7	GTGGGGTTGCTGAGAGAGAG	ACAAACAAGCGGTGATGACA	60.0	222	NA	NA	NA	NA	NA	NA	NA	NA
CaGMS1694	(AAT)16	(AAT)13	TGGAAAaCTAAATGGTGTTTGG	TCACCGGTGTCACGATAGAT	58.9	274	NA	NA	NA	NA	NA	NA	NA	NA
CaGMS1695	(TTC)8	(TTC)5	TCACACGTACGGGATTTTGA	GCCCTTCCTCCTAGACATCC	60.0	115	NA	NA	NA	NA	NA	NA	NA	NA
CaGMS1696	(TTA)9	(TTA)6	TGCACACAATCACTGCTGAC	TTGAAATGGGGTTGAAGGAC	59.4	276	NA	NA	NA	NA	NA	NA	NA	NA
CaGMS1697	(TAT)15	(TAT)12	GGCCTAAGATAATAGCAATtCCA	TTTTTACGTGTCTTTTTGTTGTCTC	58.7	159	NA	NA	NA	NA	NA	NA	NA	NA
CaGMS1698	(CTA)8	(CTA)5	TGTTGCTCcTTTCTTTGCCT	GAGCATGTGGTGAAGCAGaA	60.0	152	NA	NA	NA	NA	NA	NA	NA	NA
CaGMS1699	(AT)10	(AT)6	GCAATTTGATTTTCATTTTGGA	AACTTCTCGAGGACGCAAAA	59.0	239	NA	NA	NA	NA	NA	NA	NA	NA
CaGMS1700	(TA)10	(TA)6	TGGATGAGCCcTTCTTGAAC	TGAAATTTAAATGGGTGCCA	60.2	182	NA	NA	NA	NA	NA	NA	NA	NA
CaGMS1701	(AT)10	(AT)6	CATCGAGGCAAAATCTAAAAA	TTCAGTGATTAATAAATTGGATTTGG	57.1	272	NA	NA	NA	NA	NA	NA	NA	NA
CaGMS1702	(AG)11	(AG)7	GAGAGGGTAGGTGCATTGGA	CGAAATTGAAGGTTGGAGGA	60.1	207	NA	NA	NA	NA	NA	NA	NA	NA
CaGMS1703	(TA)10	(TA)6	TCACACAATTATAAAATGATCCACA	TGGTTGGATTACCTGCACAA	58.4	224	NA	NA	NA	NA	NA	NA	NA	NA
CaGMS1704	(AT)10	(AT)6	AACAAGTCTGCAACAACCTTCT	CACAAAGTGAAGGGGGTCAT	58.0	270	NA	NA	NA	NA	NA	NA	NA	NA
CaGMS1705	(AT)10	(AT)6	CCTtCAATCCcATAAAAagTTG	ATTGATGCAGTGCCTTTTCA	57.7	260	NA	NA	NA	NA	NA	NA	NA	NA
CaGMS1706	(AT)10	(AT)6	TGATAACGAATTAACTCACATCCA	CTTGCCTTTTTACACACCCC	58.6	231	NA	NA	NA	NA	NA	NA	NA	NA
CaGMS1707	(AG)10	(AG)6	GTTCCGAATTTGCGATCACT	TTTGCCGTTCATGGTGATAA	60.1	200	NA	NA	NA	NA	NA	NA	NA	NA
CaGMS1708	(TA)11	(TA)7	AAAAACGCAAACACCaCTCC	GTGaAATAATTAGCTCTCTGATACCAT	60.0	237	NA	NA	NA	NA	NA	NA	NA	NA
CaGMS1709	(TTTA)8	(TTTA)6	GTCATCTCATCAAGACGCCA	TCCAAGTCACTCAGTTACAAAACC	59.8	162	NA	NA	NA	NA	NA	NA	NA	NA
CaGMS1710	(AT)12	(AT)8	TGCTTAAAGAATTTGAAACGATGA	CATTAGCCTCGTTGGCATTT	60.1	210	NA	NA	NA	NA	NA	NA	NA	NA
CaGMS1711	(AAT)24	(AAT)7	TGGTGTTCCAAGAAACCATTT	TTATGTTCGGATCCAGTGTCC	59.3	216	NA	NA	NA	NA	NA	NA	NA	NA
CaGMS1712	(TTA)23	(TTA)6	CGaacaaaCAAAAaGACGAAAA	GCCGTcACATTGTTATTCCA	59.3	273	NA	NA	NA	NA	NA	NA	NA	NA
CaGMS1713	(ATT)26	(ATT)9	TCCTCGCGAATCTAACAACA	TTGAATAAGAAAGAGAGAGAAATTGA	59.4	274	NA	NA	NA	NA	NA	NA	NA	NA
CaGMS1714	(AG)16	(AG)13	CTCGGCGCTGAGATAAGAGT	GGTAGTGGTGGAGGGTCAAA	59.7	266	NA	NA	NA	NA	NA	NA	NA	NA
CaGMS1715	(TA)9	(TA)6	TTGCTTATCCACATTGCACC	GGACCTTTGCTCTGCAAGAC	59.5	102	NA	NA	NA	NA	NA	NA	NA	NA
CaGMS1716	(CT)9	(CT)6	TGCTTGCTCGGTTATGTGAG	CCAGTTGGAGGAGAGAGGAA	60.0	193	NA	NA	NA	NA	NA	NA	NA	NA
CaGMS1717	(TA)11	(TA)8	TCCAAAAATTTAGCAGGACCA	caAGTAAAAATGCGATCTTCaAA	59.6	155	NA	NA	NA	NA	NA	NA	NA	NA
CaGMS1718	(AT)9	(AT)6	TGTAAAaTATTTGATTTTAGTGTCCTG	TTTCACCAACCGTTCATTCA	57.2	260	NA	NA	NA	NA	NA	NA	NA	NA
CaGMS1719	(ATT)22	(ATT)5	CAACCAAATTTCTTCTtCTTCACA	CAGCTGTTATCCATATTTCGTGG	59.7	189	NA	NA	NA	NA	NA	NA	NA	NA

*Markers identity	Microsatellite repeat-motifs in ICC4958	Microsatellite repeat-motifs in PI489777	Forward primer sequences (5'-3')	Reverse primer sequences (5'-3')	Actual annealing temperature (0C)	Size (bp) of alleles amplified	Linkage groups (LGs)	Genetic positions (cM)	Markers used for polymorphism survey	Polymorphism information content (PIC)	Number of alleles amplified	Markers used in Figures	Markers types	GC%
CaGMS1720	(TA)9	(TA)6	TTTCTGAAAACTGTTGAATTCTTATTT	TGAAATATCGATCAAATCACGTaAA	58.1	115	NA	NA	NA	NA	NA	NA	NA	NA
CaGMS1721	(ATA)10	(ATA)8	TGGTTTCAGGTGAACTATGGG	TCTGCGCTGATCTAAAGGaAA	59.8	219	NA	NA	NA	NA	NA	NA	NA	NA
CaGMS1722	(AT)10	(AT)7	GCATTACCCTCCCAGTAAAGC	GGGAACATAGACAGGGCAAA	60.0	123	NA	NA	NA	NA	NA	NA	NA	NA
CaGMS1723	(AT)11	(AT)8	TCAATTCATGTAATTTTCGCCA	TTGACATGCAACATTCtTTTTG	60.3	129	NA	NA	NA	NA	NA	NA	NA	NA
CaGMS1724	(TA)10	(TA)7	AATTTCTCGACAGAATCAGCTAAC	TGAGCCACTGTGCTTTTGAA	58.2	280	NA	NA	NA	NA	NA	NA	NA	NA
CaGMS1725	(AT)10	(AT)7	TTTTGGTATATAATTCTCAAAAGGGA	TGTCGTCCGTCAATGGTCTA	58.6	280	NA	NA	NA	NA	NA	NA	NA	NA
CaGMS1726	(AT)9	(AT)6	CCCCCTCTCCTCTCTCTCAC	TTCCCAAACATAACCCCAGA	60.3	211	NA	NA	NA	NA	NA	NA	NA	NA
CaGMS1727	(TTA)29	(TTA)12	TCAAAGTCGGCAAATCAAAa	GCTTACTAAATTCCCTTaAAAAGCA	59.3	260	NA	NA	NA	NA	NA	NA	NA	NA
CaGMS1728	(TTA)22	(TTA)5	TGTCACCGAAGTTACCCTAAAA	TAGGACCGACCTGACCTGAG	58.7	265	NA	NA	NA	NA	NA	NA	NA	NA
CaGMS1729	(AAT)24	(AAT)7	TGGTAAGAGGACACGTGGAA	TTAGTTTGCAGTTGTTCGGA	59.1	208	NA	NA	NA	NA	NA	NA	NA	NA
CaGMS1730	(TATTTT)7	(TATTTT)6	TGTCAGGTAAAGAATCGGGC	CATCCTTGTTGGAAAGGGAA	60.1	267	NA	NA	NA	NA	NA	NA	NA	NA
CaGMS1731	(TTA)26	(TTA)9	CGATACTAAAACCGTAAAAaCCCA	CCAACCAACTTTAtcAACTTTCC	60.5	273	NA	NA	NA	NA	NA	NA	NA	NA
CaGMS1732	(TA)9	(TA)6	TTGAATTTtCTTTGCCACTAAAC	TTTTGATAATTTAACGGTGCAA	57.6	236	NA	NA	NA	NA	NA	NA	NA	NA
CaGMS1733	(AT)9	(AT)6	CAAATTTCTTTGTATCAATGAGCC	TGTTGAAACAAATCATACaTCAAA	59.1	217	NA	NA	NA	NA	NA	NA	NA	NA
CaGMS1734	(TAA)7	(TAA)5	AAAAGTAGTCCTAAGGAGCATAAAAA	CCGATCGAATCAGTTAGTCCA	57.6	232	NA	NA	NA	NA	NA	NA	NA	NA
CaGMS1735	(TA)7	(AT)7	TCATTTAACGGTCACATGCAA	GGGTGGAAATAGTAAAACGAAA	60.0	163	NA	NA	NA	NA	NA	NA	NA	NA
CaGMS1736	(ATT)30	(ATT)7	GCCACCTCATCACAAATGAA	AAGGACTGTgGATTAAAtAAAgGG	59.5	263	NA	NA	NA	NA	NA	NA	NA	NA
CaGMS1737	(TATTT)6	(TATTT)5	TGACCATACGTTCCAATTGAT	AAGATAGCATCTTGGAGATGAACA	57.8	225	NA	NA	NA	NA	NA	NA	NA	NA
CaGMS1738	(TTA)21	(TTA)6	AACTGAATCTCATTAACCTTTTGAA	AAAAACCTcAAAATAGTGTAGGTTGAA	57.6	251	NA	NA	NA	NA	NA	NA	NA	NA
CaGMS1739	(AT)8	(AT)6	GGAaTTAGCCATTAACCGCA	GTTGCAATGCCAAGGTTGTA	59.9	280	NA	NA	NA	NA	NA	NA	NA	NA
CaGMS1740	(AT)8	(AT)6	TGGATAACTTTCAACTGAGGTGG	TCCCACAAATACAGGGATTCA	60.4	276	NA	NA	NA	NA	NA	NA	NA	NA
CaGMS1741	(TA)8	(TA)6	CAAAAAGCCACGTACCACCT	CGTACTCCTCCTAATTGGTTTGA	60.0	227	NA	NA	NA	NA	NA	NA	NA	NA
CaGMS1742	(GA)10	(GA)8	CACAACACAACGGAATGGAG	ATTAAAGAAAGCGCAGGGAA	60.0	179	NA	NA	NA	NA	NA	NA	NA	NA
CaGMS1743	(TTA)7	(TTA)14	CCCTCTTCTCTACGACACCG	CGGTGGaCATCCTCGTATCT	59.9	279	NA	NA	NA	NA	NA	NA	NA	NA
CaGMS1744	(TA)10	(TA)8	AAAAGTTCTTGTGTTGCACGA	ACATGAAATTTTAATTCTTTACCATTC	58.5	205	NA	NA	NA	NA	NA	NA	NA	NA
CaGMS1745	(AT)10	(AT)8	ATGCACTTAGATTCGCCGTT	TGACTCAAATGCATGCaAGa	59.7	131	NA	NA	NA	NA	NA	NA	NA	NA
CaGMS1746	(TA)9	(TA)7	GGTTGTTGGTAAATCAAGTGTGG	GGACTTTGAGGAGAATCGAGG	60.5	202	NA	NA	NA	NA	NA	NA	NA	NA
CaGMS1747	(TA)8	(TA)6	AACAACAACAAAATGGAAGGAA	TTTGATACTTTCAAGTTTTACCGA	58.5	178	NA	NA	NA	NA	NA	NA	NA	NA
CaGMS1748	(TTA)20	(TTA)5	TCACGTCGTTAaAAATATGTCAA	AGGATTGAGTAGATGTGCCTAAA	57.4	269	NA	NA	NA	NA	NA	NA	NA	NA
CaGMS1749	(TG)8	(TG)6	ACAGGAAGTCCCCATCAACA	TCAACGGTTGGATTCGTAAA	60.4	222	NA	NA	NA	NA	NA	NA	NA	NA
CaGMS1750	(TA)8	(TA)6	CCTGGCTCAGAGTATTTCGC	TTGAATGTCCATGGCTGTGT	60.0	232	NA	NA	NA	NA	NA	NA	NA	NA
CaGMS1751	(TTTA)6	(TTTA)5	ACCATGGGAAGTGTCCAGAG	GCTTATATTTGATTCCGAATGATG	60.0	262	NA	NA	NA	NA	NA	NA	NA	NA
CaGMS1752	(TA)8	(TA)6	TTGAACAGCATTGCCATCAT	ATCCCTGCATGCATCTCTCT	60.1	208	NA	NA	NA	NA	NA	NA	NA	NA
CaGMS1753	(TCTT)7	(TCTT)6	TTTGGtCCCACACGACACT	ATTACCGAGCAACCTGCAAT	60.0	223	NA	NA	NA	NA	NA	NA	NA	NA
CaGMS1754	(AT)14	(TA)7	AACGTGAATATTGTGAGTCTGTTCA	ATGTGACATGAAGGCCACTG	60.0	217	NA	NA	NA	NA	NA	NA	NA	NA
CaGMS1755	(AG)8	(AG)6	GGATGATACGCTTTAGGGcA	GCGATTGCGATGGTTTTAAT	60.1	262	NA	NA	NA	NA	NA	NA	NA	NA
CaGMS1756	(TA)8	(TA)6	AAGAACCAAAGATTCAATCACTTTT	TTTTGCAGTGTGTACTTGTGC	58.7	197	NA	NA	NA	NA	NA	NA	NA	NA
CaGMS1757	(TA)8	(TA)6	ACTCCATCTTTTCCCTGCAA	CTTTAGGAATTGGCCCTCAC	59.7	111	NA	NA	NA	NA	NA	NA	NA	NA
CaGMS1758	(ATA)22	(ATA)7	TCTAAGGAaCAATGGCCAGG	TTTTTAGAAGAAACAGTCATGGG	60.1	233	NA	NA	NA	NA	NA	NA	NA	NA
CaGMS1759	(TA)10	(TA)8	AAAGATTGGAGGCGTGATGT	CACATGTGTCAACACCGACA	59.6	197	NA	NA	NA	NA	NA	NA	NA	NA
CaGMS1760	(TA)8	(TA)6	ACCTCTAGATTTGTCTTTGTCATGT	TGTTTGCACAAATGTCTTTCG	57.5	275	NA	NA	NA	NA	NA	NA	NA	NA
CaGMS1761	(TG)9	(TG)7	TGAACAATCCAAATGAGGAGA	TATCATGCAGCCTGAAGCAG	58.1	277	NA	NA	NA	NA	NA	NA	NA	NA
CaGMS1762	(AT)8	(AT)6	TTATGCATCCTGTTGCTCCA	GCGTTCTTCAGCCACTTTCT	60.2	235	NA	NA	NA	NA	NA	NA	NA	NA
CaGMS1763	(GA)12	(GA)10	GCCGGAGAAGGAAGAGAAAG	AAAAGGTACCCCACCCACTC	60.5	238	NA	NA	NA	NA	NA	NA	NA	NA
CaGMS1764	(AT)9	(AT)7	AAATTTTCAATGCACTTGTTTCAA	CCAGAACAATCTCTCTCGATCTC	59.9	243	NA	NA	NA	NA	NA	NA	NA	NA
CaGMS1765	(AG)10	(AG)8	CCACAGCTTCAACGACAAGA	CAATGGTAAAAGGGGCTTGA	60.0	128	NA	NA	NA	NA	NA	NA	NA	NA
CaGMS1766	(AT)8	(AT)6	TGTCGTGATTGATACCACCA	TTGTTAATTTGTGGAGAATCCGT	58.3	149	NA	NA	NA	NA	NA	NA	NA	NA
CaGMS1767	(AT)12	(AT)10	AGGTGATTGATTGTGTTCATCA	TGCATCTTTTAGGCATTCCC	57.9	235	NA	NA	NA	NA	NA	NA	NA	NA
CaGMS1768	(TTA)20	(TTA)5	GCGCAAGACCAAACTATTTTCT	CACTACTACCGTTGAAATCTGCAT	59.8	280	NA	NA	NA	NA	NA	NA	NA	NA
CaGMS1769	(TA)8	(TA)6	TCATGGGATGGTGAATGCTA	AACTTTTCAGCCACATCTCAAA	59.9	178	NA	NA	NA	NA	NA	NA	NA	NA
CaGMS1770	(AT)8	(AT)6	CACCAATTCTTTCTTCTTGTGC	AATCCGTTTATTTCAAAGGACA	58.9	262	NA	NA	NA	NA	NA	NA	NA	NA
CaGMS1771	(TTAT)7	(TTAT)6	CAGTACCTACCTCACCTTGCAC	AAATGCCTTATTACCTACGCTTT	58.8	259	NA	NA	NA	NA	NA	NA	NA	NA
CaGMS1772	(TA)8	(TA)6	TGgCACAACTAAGAAAAATAATGAA	CCCAAAAGACATACGGCTAAA	59.2	228	NA	NA	NA	NA	NA	NA	NA	NA

*Markers identity	Microsatellite repeat-motifs in ICC4958	Microsatellite repeat-motifs in PI489777	Forward primer sequences (5'-3')	Reverse primer sequences (5'-3')	Actual annealing temperature (0C)	Size (bp) of alleles amplified	Linkage groups (LGs)	Genetic positions (cM)	Markers used for polymorphism survey	Polymorphism information content (PIC)	Number of alleles amplified	Markers used in Figures	Markers types	GC%
CaGMS1773	(AG)14	(AG)12	AAATGGGCAATGGATTGAAG	TTCtCAGATGGAGtTTCCCG	59.8	137	NA	NA	NA	NA	NA	NA	NA	NA
CaGMS1774	(TA)8	(TA)6	GGGATGATATATGAGATTGCATGA	aaaaagaAATTTTTACACCTGTTCA	60.0	247	NA	NA	NA	NA	NA	NA	NA	NA
CaGMS1775	(AT)12	(AT)6	ACAAGGGTAAAGAGGTGCCC	TTCATTATACGTGGCCGTGA	60.4	262	NA	NA	NA	NA	NA	NA	NA	NA
CaGMS1776	(AT)8	(AT)6	AAATCACTCCTATCCATTATTGCTG	AAGAAACCAATTAGCATTTGCC	59.8	277	NA	NA	NA	NA	NA	NA	NA	NA
CaGMS1777	(TAT)20	(TAT)5	TTTCTCACTTATTAATTTTCTTTCACG	CTCTTGCTTCTTTCATTCCCA	58.6	216	NA	NA	NA	NA	NA	NA	NA	NA
CaGMS1778	(AT)10	(AT)8	TCGAAAACTTTTAACGACACCA	tTTCAACCATTATAGATGTCGTCTC	59.7	225	NA	NA	NA	NA	NA	NA	NA	NA
CaGMS1779	(AT)8	(AT)6	TCCAAAATTTAAAACACTCGCA	AAAAGAAGCTTTGTATACCTTTTCTGA	59.6	195	NA	NA	NA	NA	NA	NA	NA	NA
CaGMS1780	(AG)8	(AG)6	GGAGGGAGGAGGAAGAGAAG	TGGGATCATTCATTTTTCTCG	59.4	237	NA	NA	NA	NA	NA	NA	NA	NA
CaGMS1781	(CT)13	(CT)11	TTTGTTGCAGTGGTTTCTGG	CAGAGTgGGaGAAAGCAGGA	59.7	170	NA	NA	NA	NA	NA	NA	NA	NA
CaGMS1782	(CT)8	(CT)6	TACTTGGGGACTTGCTTTGG	TGAGGCTAAAAATCAGCAACC	60.1	113	NA	NA	NA	NA	NA	NA	NA	NA
CaGMS1783	(AT)9	(AT)7	GGGTTAGGGAAGTTTTtGCC	TCACTTGCGAATACCAAGCA	59.8	190	NA	NA	NA	NA	NA	NA	NA	NA
CaGMS1784	(AG)8	(AG)6	TGAAAATGAGTGTTTGGGGG	TTCACCTTCCATTCCTTTCG	60.7	253	NA	NA	NA	NA	NA	NA	NA	NA
CaGMS1785	(AT)12	(AT)10	GCTGAGGTGTtCAGgAAACAA	AGATCGAAAAACTTCACACGC	60.3	149	NA	NA	NA	NA	NA	NA	NA	NA
CaGMS1786	(AAT)23	(AAT)8	AGCTTTGTAATTTTGTCAATCCA	TTCTGTATCAATTTTTATTTGACATGA	57.9	276	NA	NA	NA	NA	NA	NA	NA	NA
CaGMS1787	(AT)13	(AT)11	TGCAATAAAGAATTATCAAGAGTGTCA	TCAAAATTTCACAAGCATTAGCA	60.4	154	NA	NA	NA	NA	NA	NA	NA	NA
CaGMS1788	(CT)9	(CT)7	CTCAATTTCTTCGACCTCCG	GGCTCAAAATGGTTCAAGGA	59.8	214	NA	NA	NA	NA	NA	NA	NA	NA
CaGMS1789	(AT)9	(AT)7	AATCACTCACTCTCCTATGTATGGTT	TATGCCCGCAATACATGAAA	58.6	161	NA	NA	NA	NA	NA	NA	NA	NA
CaGMS1790	(TA)8	(TA)6	CGACGCGAGTGTCAAAAATA	CGAGGATCGACGTTTCTATTG	59.9	232	NA	NA	NA	NA	NA	NA	NA	NA
CaGMS1791	(AT)8	(AT)6	AAACACCCTTTCCAAGTGTCA	GGAGGAAGTAGTCGGAGAGGA	59.5	108	NA	NA	NA	NA	NA	NA	NA	NA
CaGMS1792	(ATTT)6	(ATTT)5	ACTAAGCGACAGTTGGGGTG	GCTGCCATGAGAATCACAAA	60.2	216	NA	NA	NA	NA	NA	NA	NA	NA
CaGMS1793	(TA)9	(TA)7	AACAAAAAGAAGCTTGGAGCA	GCATGCATTTTAAtGcCAAA	59.1	214	NA	NA	NA	NA	NA	NA	NA	NA
CaGMS1794	(CA)8	(TTAT)5	GGCTCCTTCATTCCTTCCTT	CACATCTGCAATGTTGCAcTC	59.7	272	NA	NA	NA	NA	NA	NA	NA	NA
CaGMS1795	(TTAT)7	(TA)11	TTtGGATAAAGAAATTAAAGTAaTGGA	ATGCACAAAGCATTCAATCA	57.9	183	NA	NA	NA	NA	NA	NA	NA	NA
CaGMS1796	(TA)8	(TA)6	TTGATCGTTCTCCCGAGACT	TGCCACCCACATGtTTTATC	59.8	169	NA	NA	NA	NA	NA	NA	NA	NA
CaGMS1797	(ATT)26	(ATT)11	TAGCAaCAAAAATGGGGgAG	CTCATAGTGCTGCGTGTCGT	59.9	235	NA	NA	NA	NA	NA	NA	NA	NA
CaGMS1798	(TA)10	(TA)8	TGCTTGTTTCCAATTGCTGA	CTCAAAATCGAGAGGCTGCT	60.4	268	NA	NA	NA	NA	NA	NA	NA	NA
CaGMS1799	(TA)9	(TA)7	CCTTCAACTCCTCCACAGGT	cATTGCAttTGCTGTGACAA	59.1	247	NA	NA	NA	NA	NA	NA	NA	NA
CaGMS1800	(AG)9	(AG)7	TTTAATCCGAATCCTTCCCC	TTTTGGATGAAGAAATGGGC	60.1	202	NA	NA	NA	NA	NA	NA	NA	NA
CaGMS1801	(TA)8	(TA)6	CTGGGTTCTGTCCTGGTGAT	CCCACCAGATCACCTTTCTT	60.0	163	NA	NA	NA	NA	NA	NA	NA	NA
CaGMS1802	(TG)10	(TG)8	TAGGGGATGCTATGGTTGGT	TTCGTAAGCACCATTACCCC	59.3	257	NA	NA	NA	NA	NA	NA	NA	NA
CaGMS1803	(AT)8	(AT)6	GTTCGTCACCACCTACCTGC	GGGAGTGAgGGGGTTGTAAT	60.6	268	NA	NA	NA	NA	NA	NA	NA	NA
CaGMS1804	(AT)9	(AT)7	TCAATTGCTTAGGCTTTTACCC	TCTTGTCACAACTCAATTGACAAA	59.6	234	NA	NA	NA	NA	NA	NA	NA	NA
CaGMS1805	(AT)9	(AT)7	AGCGCCGTAGTGATGAGTCT	TGCGACTTATTTATTATGCGTGA	60.0	269	NA	NA	NA	NA	NA	NA	NA	NA
CaGMS1806	(AT)8	(AT)6	TTGCCTCCACGTTCAATTATC	TTGTGTTGCAGCtTTGTTGA	60.0	216	NA	NA	NA	NA	NA	NA	NA	NA
CaGMS1807	(TA)8	(TA)6	TTCCTTCCTTTTATTCCCcTTT	TGATCTGTgGGAAAATGCAG	59.4	266	NA	NA	NA	NA	NA	NA	NA	NA
CaGMS1808	(AG)11	(AG)9	AAAAaTGAaCGGAAGCAACG	ATCGCTCCAAACCCCTTTAT	60.1	205	NA	NA	NA	NA	NA	NA	NA	NA
CaGMS1809	(AT)8	(AT)6	GATGCACATGATATAAAGAGACATGA	TGACATGTGTATCAAATACATCACaAA	59.4	160	NA	NA	NA	NA	NA	NA	NA	NA
CaGMS1810	(TA)8	(TA)6	TGGTGTTGCATGTGTGAATG	GGTCCGATTTCTAGTCCGGT	60.0	219	NA	NA	NA	NA	NA	NA	NA	NA
CaGMS1811	(TAAC)7	(TAAC)6	CACTTGTTGGCATTTTACTTGC	AACGTTCTTGGtTTGTGACTGA	59.7	278	NA	NA	NA	NA	NA	NA	NA	NA
CaGMS1812	(TA)9	(TA)7	GGGTTGATTAGACACCGCAT	CTTCCACGTGCTCTTCAATTC	59.8	256	NA	NA	NA	NA	NA	NA	NA	NA
CaGMS1813	(AT)11	(AT)9	GGTGGTTTGATGAGAGGTCAA	CTTTCGTGCCGAATAAGCAT	60.0	264	NA	NA	NA	NA	NA	NA	NA	NA
CaGMS1814	(TA)8	(TA)6	CAATACATGGTGTTTATTGGgA	TTAAGCCAACGCAAACATCA	57.3	240	NA	NA	NA	NA	NA	NA	NA	NA
CaGMS1815	(TA)8	(TA)6	TTGAAAACTTCCTGCATGTG	CAAATATATTAAtTCATCACaCCCaAA	57.3	209	NA	NA	NA	NA	NA	NA	NA	NA
CaGMS1816	(GA)9	(GA)7	CAATGGCCGACCACTTATCT	TGTGGTCCATGGTTCAAAAT	60.0	126	NA	NA	NA	NA	NA	NA	NA	NA
CaGMS1817	(GA)9	(GA)7	TGGACATTCTAGAAGAGAAGCAAA	ACTTTTCCCCTTCCCTGTGT	59.6	196	NA	NA	NA	NA	NA	NA	NA	NA
CaGMS1818	(TA)8	(TA)6	GGTCTTATGGATCTTGAATGTATCA	TAAACGGGTTGGGCACTTTA	58.5	251	NA	NA	NA	NA	NA	NA	NA	NA
CaGMS1819	(TG)10	(TG)8	TGGTTGCTCAGAGTCAGTGG	GGATTAAGGAAGCAAGGTGAAA	60.0	218	NA	NA	NA	NA	NA	NA	NA	NA
CaGMS1820	(AT)9	(AT)7	TCCAACAACTAATTCTTGAGCAAC	GAGAAATCGGCACTCACTCC	59.7	226	NA	NA	NA	NA	NA	NA	NA	NA
CaGMS1821	(AC)8	(AC)6	AATCTCACAAAGCGTGACGA	TTTgGGGTTTAgGGTGTTACAT	59.4	205	NA	NA	NA	NA	NA	NA	NA	NA
CaGMS1822	(AT)8	(AT)6	TGTGCATCATCTCCACCAAT	TGGGAAGTTGGATTGAATAGC	59.9	140	NA	NA	NA	NA	NA	NA	NA	NA
CaGMS1823	(AT)8	(AT)6	ATCAAAAAGCGTCCTTCACG	AGTTTGGCGCTTGACTTTGT	60.2	258	NA	NA	NA	NA	NA	NA	NA	NA
CaGMS1824	(AT)10	(AT)8	GCATGCCCAATTTTTGACTT	CGGCGTTTTGGTAATCACTT	59.9	259	NA	NA	NA	NA	NA	NA	NA	NA
CaGMS1825	(TA)9	(TA)7	ATGCATGGATGTGGCGTAT	TAGCATTTCCCAACCTTTGC	59.8	260	NA	NA	NA	NA	NA	NA	NA	NA

*Markers identity	Microsatellite repeat-motifs in ICC4958	Microsatellite repeat-motifs in PI489777	Forward primer sequences (5'-3')	Reverse primer sequences (5'-3')	Actual annealing temperature (0C)	Size (bp) of alleles amplified	Linkage groups (LGs)	Genetic positions (cM)	Markers used for polymorphism survey	Polymorphism information content (PIC)	Number of alleles amplified	Markers used in Figures	Markers types	GC%
CaGMS1826	(TA)8	(TA)6	GCATTGTCCAAATATGACAAACA	AACTTTATGTTAATCCATATTAAACCG	59.8	268	NA	NA	NA	NA	NA	NA	NA	NA
CaGMS1827	(AT)8	(AT)6	TCGTGGTGATTCCCAATACA	TGtTTTGGGTGGtGGTTCTC	59.8	157	NA	NA	NA	NA	NA	NA	NA	NA
CaGMS1828	(AT)9	(AT)7	ACtCAAAACCGTTGTCCAAA	CCTTTGCATATGGCGAATTT	58.1	176	NA	NA	NA	NA	NA	NA	NA	NA
CaGMS1829	(AT)6	(AT)6	CGAGAGCTCTGATACCATCTCA	AACCTTATTCACCCCATTTGC	59.6	278	NA	NA	NA	NA	NA	NA	NA	NA
CaGMS1830	(AT)8	(AT)6	CGTGTGTTTCTGTTTTTGCG	CTGGGTCCCACATTAGCTGT	60.3	244	NA	NA	NA	NA	NA	NA	NA	NA
CaGMS1831	(AATA)6	(AATA)5	TCCTCGAGATCCAAAAGCAT	GTCCAATTCCCCCTTTCATT	59.8	273	NA	NA	NA	NA	NA	NA	NA	NA
CaGMS1832	(AC)9	(AC)7	TTAAGTCTGGCCCATCAAGG	TGCTTTGTCAACCTTGCTACA	60.1	239	NA	NA	NA	NA	NA	NA	NA	NA
CaGMS1833	(TA)9	(TA)7	TCACTGTCTTTTCCTCCAGC	TAGGCTTCCCTTGGTTGTTG	58.0	249	NA	NA	NA	NA	NA	NA	NA	NA
CaGMS1834	(TA)9	(TA)7	CGAGGTATGCTAGAGTATGTTtGTTT	TGAACTTTTGATCCCTCGGT	59.3	231	NA	NA	NA	NA	NA	NA	NA	NA
CaGMS1835	(AT)12	(AT)10	TCATCGTTAAAAGAATAGTGCTATCAG	AGTGTCTAACACGTGCGTCG	59.4	148	NA	NA	NA	NA	NA	NA	NA	NA
CaGMS1836	(CA)8	(CA)6	CGATCCTTTCCAACTCCAAA	ATTTGAAGCGATGGTGTTCC	60.0	186	NA	NA	NA	NA	NA	NA	NA	NA
CaGMS1837	(CT)10	(CT)8	CGCCGTTtCCATTtCTTTTA	GTTATGGAGGAGGGCACTGA	60.1	116	NA	NA	NA	NA	NA	NA	NA	NA
CaGMS1838	(GA)8	(GA)6	TTTCCAAATCACACATACACACA	GTTCAAAAGGTGGCAACACA	58.9	187	NA	NA	NA	NA	NA	NA	NA	NA
CaGMS1839	(TA)9	(TA)7	TGCCTAGTGACTTTAGTTtATTTTTGG	TGGTTGCTATCCCCACCTAT	60.3	224	NA	NA	NA	NA	NA	NA	NA	NA
CaGMS1840	(AT)9	(AT)7	CATCTCAACCCCTGTTCACC	TCGATCTAATTAATAATCGCATTAAA	60.4	192	NA	NA	NA	NA	NA	NA	NA	NA
CaGMS1841	(TA)8	(TA)6	TTTtGTTCTtgTTGTTGTCaTCG	GCCCaTGTAAAAAtAATTGGCT	60.1	274	NA	NA	NA	NA	NA	NA	NA	NA
CaGMS1842	(TC)13	(TG)6	ACAAGTCCATGTGGCAATGA	CGGGTGGTGAAATAAAATGG	60.0	112	NA	NA	NA	NA	NA	NA	NA	NA
CaGMS1843	(TG)8	(TA)6	AAACATAAGCTTTTGATAAAGAATCCA	AGAAAGTGCAACAACCGGAC	59.9	264	NA	NA	NA	NA	NA	NA	NA	NA
CaGMS1844	(AATA)8	(TTA)5	GTAAATCGCAACCATCGGTC	TTGTTGATTGATCCATTTGATTG	60.3	260	NA	NA	NA	NA	NA	NA	NA	NA
CaGMS1845	(AT)8	(TC)6	CCATGTTAACAATGAAAAGAGGAA	TCTGAGGGGATTTGAATTTTG	59.4	280	NA	NA	NA	NA	NA	NA	NA	NA
CaGMS1846	(AT)8	(AT)6	GACATGGCAGTCATTGATGG	AAGTTCAGGGGTTTGGGTTT	59.9	200	NA	NA	NA	NA	NA	NA	NA	NA
CaGMS1847	(TA)8	(TA)6	TGTGGACTGGCTTGTGTCAT	CAAAAGGGTCACAAAAATGC	60.2	245	NA	NA	NA	NA	NA	NA	NA	NA
CaGMS1848	(CT)8	(CT)6	GATGGATAGGCTGTGACGCT	TTATTTGCAGCGGAATTTCA	60.2	216	NA	NA	NA	NA	NA	NA	NA	NA
CaGMS1849	(AG)8	(AG)6	TTCTTGAATCTTTGGGTGGC	GAGAAACCCTAGGAGGGACG	60.1	167	NA	NA	NA	NA	NA	NA	NA	NA
CaGMS1850	(AT)9	(AT)7	GGCATGTTTGGAGCAAGATT	TGGTTGTTTTGTGTGTGGTG	60.1	228	NA	NA	NA	NA	NA	NA	NA	NA
CaGMS1851	(TTTA)6	(TTTA)5	TTGTCGCAAACTATGGATGC	TGATTGTAAAGTCAAGTGTGCCT	59.7	244	NA	NA	NA	NA	NA	NA	NA	NA
CaGMS1852	(TA)8	(TA)6	CCCTTCTCGACGAATGAAGA	TTCATAACCTGAAGTGTTGGTG	60.3	175	NA	NA	NA	NA	NA	NA	NA	NA
CaGMS1853	(AT)8	(AT)6	AACAAAGCCACAGGAAAACG	GCCATTCTCATGAGGGAAAA	60.1	202	NA	NA	NA	NA	NA	NA	NA	NA
CaGMS1854	(AT)8	(AT)6	ATCCCAAAAGCCTTTACCGT	TCATCTGAAGTCAAGTGGCG	59.8	258	NA	NA	NA	NA	NA	NA	NA	NA
CaGMS1855	(AT)8	(AT)6	TCCACTTTGATCATTTTGATTTG	TGGTTTTGTGCACACGTAAGA	59.0	196	NA	NA	NA	NA	NA	NA	NA	NA
CaGMS1856	(AATA)7	(AATA)6	AAAATAGGGGATTAAAACTGCAT	TTGATTTTGCTTTGAGACATGAA	57.3	224	NA	NA	NA	NA	NA	NA	NA	NA
CaGMS1857	(CA)9	(CA)7	TCTGGTGTCCGATGATGACT	TGAAGTTTCACGTTGTTTAATTTTG	59.1	262	NA	NA	NA	NA	NA	NA	NA	NA
CaGMS1858	(TA)9	(TA)7	GCTCCATAAATATTGAATTGTTTGC	AtTATCCCAATCGGACTTCG	60.1	232	NA	NA	NA	NA	NA	NA	NA	NA
CaGMS1859	(AT)8	(AT)6	GAACGGCGACTTCCTAAATG	TGTCAAAATGTTACAGCCAGC	59.7	157	NA	NA	NA	NA	NA	NA	NA	NA
CaGMS1860	(AT)10	(AT)8	TGCGACACACTAAaCCcAAA	CAtTTCTcCCCCTGtTCCAT	60.1	134	NA	NA	NA	NA	NA	NA	NA	NA
CaGMS1861	(AT)8	(AT)6	TCGAATATTACCGATAATTCTACTGAA	ATATGTGCATgGGGAgTGC	58.7	198	NA	NA	NA	NA	NA	NA	NA	NA
CaGMS1862	(TA)8	(TA)6	TGTTTTCAGCAAACTTTGTGTACT	AGGGTCAATCAAGCAAGACG	58.1	264	NA	NA	NA	NA	NA	NA	NA	NA
CaGMS1863	(AT)8	(AT)6	TCCTGGAAATGGAGACCAAC	CAAAAGGTCAGATTTCAAAgGC	59.9	138	NA	NA	NA	NA	NA	NA	NA	NA
CaGMS1864	(TA)9	(TA)7	AGGTTTCATGTGATGGTGGAG	GGAAGAAAGCAAAAGGAGTGC	59.8	277	NA	NA	NA	NA	NA	NA	NA	NA
CaGMS1865	(AT)12	(AT)10	CAACGTTCCAAAGTAGACAAAGAA	TTCTCCCAACTTTGTTCCAA	59.7	209	NA	NA	NA	NA	NA	NA	NA	NA
CaGMS1866	(AT)9	(AT)7	TGGACAATCCGTGGTGTAGA	TGAATGATTTCACCATTTCCAA	60.0	251	NA	NA	NA	NA	NA	NA	NA	NA
CaGMS1867	(AT)8	(AT)6	CATCCGAACACATGGTGTGA	AGGGTgATCCTTGACATTGAG	61.5	272	NA	NA	NA	NA	NA	NA	NA	NA
CaGMS1868	(TA)8	(TA)6	TCACCTTCAAGTACACCACCA	TGAAAAAGTTCATTAAACATCATTG	59.0	250	NA	NA	NA	NA	NA	NA	NA	NA
CaGMS1869	(AT)9	(AT)7	GAGTGGGATTTTGGAGGTGA	TTTGAAGGGTTTTAGAGGACTGA	59.9	187	NA	NA	NA	NA	NA	NA	NA	NA
CaGMS1870	(TA)9	(TA)7	GCATGAATTAAACCCTACTGAAACA	TAGCGAGTGCGATTTTCAAG	60.6	279	NA	NA	NA	NA	NA	NA	NA	NA
CaGMS1871	(TA)8	(TA)6	TGTGTTGAATTGTTGTGACGTT	CGTCGCAACTCTATTGACAAAT	59.0	254	NA	NA	NA	NA	NA	NA	NA	NA
CaGMS1872	(TA)9	(TA)7	GACTTGTCGAACTTGCGTGA	CACCTGAAACAGATTTGTGGG	60.0	168	NA	NA	NA	NA	NA	NA	NA	NA
CaGMS1873	(TC)10	(TC)8	GCTTTGGGGTGGATTGTCTA	TGGTAAAGGCAACGATGACA	59.9	123	NA	NA	NA	NA	NA	NA	NA	NA
CaGMS1874	(AT)10	(AT)8	CCCATtGAGCTTTTCATTTG	CCACGGTTCGATTCTTGATT	58.2	221	NA	NA	NA	NA	NA	NA	NA	NA
CaGMS1875	(AT)8	(AT)6	GGGGAATATTTTGTTGTCGG	ATACTTGGGCATCCAACCAA	59.1	267	NA	NA	NA	NA	NA	NA	NA	NA
CaGMS1876	(ATAA)7	(ATAA)6	CTGCATGAAGATGAGCAAGA	TTCGAGTTCTCCGTCTTTTTCT	57.6	175	NA	NA	NA	NA	NA	NA	NA	NA
CaGMS1877	(TA)8	(TA)6	ATCCTATGCAACTCATCCGC	CAACTCAATGGTTGATTTTGAA	60.1	201	NA	NA	NA	NA	NA	NA	NA	NA
CaGMS1878	(AT)8	(AT)6	TGAACTAATTTACTTGCCATATGTTTG	AACCAGTAAGGCTTCCAGCA	59.8	211	NA	NA	NA	NA	NA	NA	NA	NA

*Markers identity	Microsatellite repeat-motifs in ICC4958	Microsatellite repeat-motifs in PI489777	Forward primer sequences (5'-3')	Reverse primer sequences (5'-3')	Actual annealing temperature (0C)	Size (bp) of alleles amplified	Linkage groups (LGs)	Genetic positions (cM)	Markers used for polymorphism survey	Polymorphism information content (PIC)	Number of alleles amplified	Markers used in Figures	Markers types	GC%
CaGMS1879	(TA)13	(TA)11	TCTGACATCATGtATGAAGTTGGTT	GTACCCTTTGATCCAGCGaA	59.8	116	NA	NA	NA	NA	NA	NA	NA	NA
CaGMS1880	(TTA)19	(TTA)6	AAAGGAGTTTGGAGATTTGAAAAa	TTTTTAAAAGaAATCTCTCCAGCTA	59.6	240	NA	NA	NA	NA	NA	NA	NA	NA
CaGMS1881	(TA)10	(TA)8	CGTTTAAAAATTATGGTAGCATGG	TGTGCAACGAGTAATGGGAG	59.0	271	NA	NA	NA	NA	NA	NA	NA	NA
CaGMS1882	(AATA)7	(AATA)6	AAAATTTGCAAACGTTTATGTTTTA	ACTATAGTACCCAcCCCGCC	58.3	202	NA	NA	NA	NA	NA	NA	NA	NA
CaGMS1883	(TA)10	(TA)8	TGAGGCACATTTCCATTGAT	TTGGCTaAAAAGCATCTGAGTTC	58.9	250	NA	NA	NA	NA	NA	NA	NA	NA
CaGMS1884	(TA)8	(TA)6	TCTCTTTCCCTTTCACTCCTTG	GGTGGTGTGGACCAGAAAGT	59.9	249	NA	NA	NA	NA	NA	NA	NA	NA
CaGMS1885	(AT)9	(AT)7	TGAAGATTTCAATTGATTTCCAA	GGGAAGGATTGAGATGGACC	58.6	247	NA	NA	NA	NA	NA	NA	NA	NA
CaGMS1886	(AT)8	(AT)6	TCTCGTCATAACTGAATTGACAAA	TTTGTGTTGAATTGCTTAGGCT	58.8	247	NA	NA	NA	NA	NA	NA	NA	NA
CaGMS1887	(AT)8	(AT)6	CTCGTTGTCTTGTCTCTGCG	CCATAGTTCCAAGTTGCATCA	59.8	234	NA	NA	NA	NA	NA	NA	NA	NA
CaGMS1888	(AT)9	(AT)7	TTGCAATGAACAATCAAACG	CGAAAGTGGATATTTTGTCTTCA	58.2	201	NA	NA	NA	NA	NA	NA	NA	NA
CaGMS1889	(TTA)18	(TTA)5	TTtCCTTtcATTTTAATGCATTTtC	TCAACGAGGTTGCATACCAG	59.8	256	NA	NA	NA	NA	NA	NA	NA	NA
CaGMS1890	(ATA)7	(ATA)6	GGACCTCAAaCCCAAAACAA	GTGTAAACATTCGTGCGGTG	59.8	257	NA	NA	NA	NA	NA	NA	NA	NA
CaGMS1891	(TAT)9	(TAT)8	ATTTTGGCCACACTGGAGTC	GGAATAGAAATTGTTGGCGTTT	60.0	220	NA	NA	NA	NA	NA	NA	NA	NA
CaGMS1892	(TAT)7	(TAT)6	TCTGGTAAGCAGCATAGTGTTTTT	TGCCTATATTCtTTTTCTTGTGAGA	59.4	280	NA	NA	NA	NA	NA	NA	NA	NA
CaGMS1893	(TTG)6	(TTG)5	TTTCGTGTAGACCATCTGTTTGA	GTCGAGGACCCATTGAACAT	59.7	273	NA	NA	NA	NA	NA	NA	NA	NA
CaGMS1894	(A)10	(AT)8	CTTCGATGTGTGCTTTGACG	ATCATGCACGTCATCTTGCT	60.4	185	NA	NA	NA	NA	NA	NA	NA	NA
CaGMS1895	(TAT)6	(TAT)5	GCATAATTGCTCATGTGATCCT	TTTTGTTTGAAAATTTGGATGAA	59.1	175	NA	NA	NA	NA	NA	NA	NA	NA
CaGMS1896	(AAT)21	(AAT)20	TTGAGTTTGCTTCTCACCTTCA	TCAAAATTGGaAAATTTGTAACGA	60.0	261	NA	NA	NA	NA	NA	NA	NA	NA
CaGMS1897	(TGT)6	(TGT)5	CTGCTTCCTCCTCCTTGTTG	TGACCCAACCAACAACCTCT	60.0	210	NA	NA	NA	NA	NA	NA	NA	NA
CaGMS1898	(ATA)6	(ATA)5	AGGGACCACAGCATTGGTAG	TCATGTGATTGTGGGCCTAA	60.0	173	NA	NA	NA	NA	NA	NA	NA	NA
CaGMS1899	(AAT)6	(AAT)5	CCCTCCAAAAGGATTCTTCC	TCCAATGGTAACGCCTCTTC	59.9	260	NA	NA	NA	NA	NA	NA	NA	NA
CaGMS1900	(TGG)8	(TGG)7	GAAGGATGAGGAGGAGGGAC	AATAACCGAAGCAATGACGG	60.0	116	NA	NA	NA	NA	NA	NA	NA	NA
CaGMS1901	(CTG)6	(CTG)5	TAGTTTATTGCCTGCCCCTG	ATCAGCATCATCACCATCCA	60.1	178	NA	NA	NA	NA	NA	NA	NA	NA
CaGMS1902	(TTA)6	(TTA)5	AAAAaGTACAGAAAaTTCAAGGAAAAa	CAATTTTCTCCTATTTGGCAGG	59.2	150	NA	NA	NA	NA	NA	NA	NA	NA
CaGMS1903	(TAT)6	(TAT)5	AAAaTTTTGCTCAAAGTTAGCACC	TGGTggGGAAACTGACTAGC	60.1	134	NA	NA	NA	NA	NA	NA	NA	NA
CaGMS1904	(TTA)8	(TTA)7	GAATGAGGTTGGGAGTTGGA	AGTTTGTGAATTTCGCCGTT	59.9	267	NA	NA	NA	NA	NA	NA	NA	NA
CaGMS1905	(AGA)7	(AGA)6	CCTTGAGAAGGTTTTTGTTGATG	GGATGCTTTTGCTGGGAATA	60.0	177	NA	NA	NA	NA	NA	NA	NA	NA
CaGMS1906	(TAT)6	(TAT)5	TTCAAATGTTACTTACACCTCTACTCG	TGAATTGATTTTATTGGCGCT	59.3	252	NA	NA	NA	NA	NA	NA	NA	NA
CaGMS1907	(ATT)21	(ATT)8	TTGGACAGCGCACTTAAAGA	TCGTTCTGATACAACTTGATGGA	59.6	272	NA	NA	NA	NA	NA	NA	NA	NA
CaGMS1908	(AGA)6	(AGA)5	CTTGGATCCATCTCAGTGCT	TGAGGATTTGGTGAAGGGTT	57.8	243	NA	NA	NA	NA	NA	NA	NA	NA
CaGMS1909	(AGA)7	(AGA)6	GTTTGGGACTTTTGGGGTTT	AGTTTAAGGCGGCTACGGAT	60.1	232	NA	NA	NA	NA	NA	NA	NA	NA
CaGMS1910	(TGA)6	(TGA)5	AGGCTTGAGATACGGTGGTG	CGTCCCTTATTTCCCCATTT	60.1	186	NA	NA	NA	NA	NA	NA	NA	NA
CaGMS1911	(TAT)6	(TAT)5	TGAACAGCAAAGCTAAAGAAAAGA	TGAATCATTTTGCAATAACATGAATA	59.7	177	NA	NA	NA	NA	NA	NA	NA	NA
CaGMS1912	(AAC)6	(AAC)5	TCAGAATCACCCAATGACGA	TTGGCCTTTCTGTGCATCTA	60.0	201	NA	NA	NA	NA	NA	NA	NA	NA
CaGMS1913	(TAT)6	(TAT)5	ATGCAACTTTCAAACCCACC	CATGCCGAGGAACAATCAAT	59.8	270	NA	NA	NA	NA	NA	NA	NA	NA
CaGMS1914	(TTA)7	(TTA)6	AGCAGATAAGGGGAGGCAAT	GCATACACATATCTCgGTATTCCtt	60.1	130	NA	NA	NA	NA	NA	NA	NA	NA
CaGMS1915	(CTT)9	(CTT)8	CCGATGTCAACAGTTTTACCG	GCGTGGTTTAGGAGGCATTA	60.4	234	NA	NA	NA	NA	NA	NA	NA	NA
CaGMS1916	(AGA)8	(AGA)7	GAGGATTTTGATGGCGAAGA	GTCTGAGACGAATCCGGAAA	60.2	205	NA	NA	NA	NA	NA	NA	NA	NA
CaGMS1917	(ATA)6	(ATA)5	AAAAGTGAATGCAAATCAACAA	GGCTTTCATTTAGGGATGTGG	57.4	271	NA	NA	NA	NA	NA	NA	NA	NA
CaGMS1918	(ATA)18	(ATA)5	TCAAGGGTTTGCTACTTGGG	TTTAACAAtTTTCTCATGTTTTGTAGA	60.1	256	NA	NA	NA	NA	NA	NA	NA	NA
CaGMS1919	(TTA)6	(TTA)5	ATCTCGCTGCTGAGGTTTGT	CCAAAAGCTTGCTGAGTTCC	60.0	278	NA	NA	NA	NA	NA	NA	NA	NA
CaGMS1920	(ATA)9	(ATA)8	GCTTCAATCGGCTACCTTTG	TTTGATAAATGAATTTAGTGGGGA	59.8	199	NA	NA	NA	NA	NA	NA	NA	NA
CaGMS1921	(AAT)9	(AAT)8	CAGCCCAAATTGTTTAATTTCA	CCAAGCAGAGCAAGAAGAAAA	59.0	279	NA	NA	NA	NA	NA	NA	NA	NA
CaGMS1922	(TAT)6	(TAT)5	TACAACAACCCAAATGCCAA	TGTAAAAGGCTTCCAATGGC	59.8	238	NA	NA	NA	NA	NA	NA	NA	NA
CaGMS1923	(TC)13	(TC)12	GCTTCGATACGGATACGGTC	CAAGGAAGGAACGAACGAGA	59.6	125	NA	NA	NA	NA	NA	NA	NA	NA
CaGMS1924	(ATG)6	(ATG)5	ATGTCCTTTCCAAGTGGTGC	AAAGGAACCTATAAGGCCACAA	60.0	264	NA	NA	NA	NA	NA	NA	NA	NA
CaGMS1925	(AAC)6	(AAC)5	TACTTGGCCAACATCAGCAG	GATACTCCGTGACTGCGACA	59.9	148	NA	NA	NA	NA	NA	NA	NA	NA
CaGMS1926	(AAT)6	(AAT)5	TCGTCATCGGGGAATAGAAG	TCTCACGCTTACTGTGTCCG	60.0	156	NA	NA	NA	NA	NA	NA	NA	NA
CaGMS1927	(ATA)19	(ATA)6	GCTAAAAaGCGTGGGAACAG	ATTTGCGGCGTTTGTAATTT	59.9	276	NA	NA	NA	NA	NA	NA	NA	NA
CaGMS1928	(AT)7	(AT)6	TGATATCCATTTCCACCCGT	TCGAATTTCtTTTTGTTTTGGAA	60.0	270	NA	NA	NA	NA	NA	NA	NA	NA
CaGMS1929	(ACT)6	(ACT)5	AAGCTTGAATTGCGCTGTTT	GCCTCCATGGTGTCCTTAAA	60.0	261	NA	NA	NA	NA	NA	NA	NA	NA
CaGMS1930	(TTA)6	(TTA)5	TCAGTTTCCTTTTTGTTGAACTC	AAAGAAATGAACTAAAACTGTCGACTT	57.6	229	NA	NA	NA	NA	NA	NA	NA	NA
CaGMS1931	(TAA)6	(TAA)5	TGTTCGTCTTGACCGTACTCA	CCCGTGCATTACAAGTTAGG	59.4	123	NA	NA	NA	NA	NA	NA	NA	NA

*Markers identity	Microsatellite repeat-motifs in ICC4958	Microsatellite repeat-motifs in PI489777	Forward primer sequences (5'-3')	Reverse primer sequences (5'-3')	Actual annealing temperature (0C)	Size (bp) of alleles amplified	Linkage groups (LGs)	Genetic positions (cM)	Markers used for polymorphism survey	Polymorphism information content (PIC)	Number of alleles amplified	Markers used in Figures	Markers types	GC%
CaGMS1932	(TAA)6	(TAA)5	CAATTCAACGCAACAATGCT	TCACGGTGAGTCGTTGTCAT	59.7	235	NA	NA	NA	NA	NA	NA	NA	NA
CaGMS1933	(GTT)7	(GTT)6	TGTTTGGATCATAACCCCTG	CAATAGCAAAAATGGCCAAGA	58.3	241	NA	NA	NA	NA	NA	NA	NA	NA
CaGMS1934	(TCA)7	(TCA)6	CCATTGAAAGCAAAGGCTTC	CTATCTTTTCCCCCAAAgGC	59.8	116	NA	NA	NA	NA	NA	NA	NA	NA
CaGMS1935	(TTA)18	(TTA)5	TGTTGGCACGTTTACTCAAAT	TCAAAGTTAACGCGGTTGAG	58.2	280	NA	NA	NA	NA	NA	NA	NA	NA
CaGMS1936	(TAT)6	(TAT)5	TTCTGCATTGTCCCTTTGGT	AGCTGTCCATGTGAATGTGA	60.5	258	NA	NA	NA	NA	NA	NA	NA	NA
CaGMS1937	(TTA)7	(TTA)6	CTCTTTCGCTCTCTCTGCAA	GCAAGTTGCAAAACAAAACC	58.6	239	NA	NA	NA	NA	NA	NA	NA	NA
CaGMS1938	(TAA)14	(AAT)5	TGTTtGGTCGATTAATAAGCAACTT	AGCATGAAGTCGTTAACAAAAAG	60.3	207	NA	NA	NA	NA	NA	NA	NA	NA
CaGMS1939	(TTA)10	(TTA)9	TTtGTTTTGTGACACGCTTTC	CCAGTTGCCCTGTCCATACT	58.9	153	NA	NA	NA	NA	NA	NA	NA	NA
CaGMS1940	(ATT)6	(ATT)5	TTGTTTGGTCTCCCCAAAAG	CACCGCACCATTTTATGAAG	59.9	269	NA	NA	NA	NA	NA	NA	NA	NA
CaGMS1941	(TGT)8	(TGT)7	CGTTAGGGTTACGGCTTTGA	AAACCTGTGTCATCATCGCA	60.1	120	NA	NA	NA	NA	NA	NA	NA	NA
CaGMS1942	(TAA)8	(TAA)7	CGAACAGAAAAAGTAAGCATTTATCA	TCCTTTTCGGTTGACTTGCT	60.1	207	NA	NA	NA	NA	NA	NA	NA	NA
CaGMS1943	(CTT)6	(CTT)5	CCCATCTTTCAGGGAAAACA	CTCTTCCTTTGGTGGAGCTG	59.9	182	NA	NA	NA	NA	NA	NA	NA	NA
CaGMS1944	(TTA)27	(TTA)14	CAAGCTCAAAATTCCAAATTCA	CTCCTAACAAcCCCAGATTGA	59.2	273	NA	NA	NA	NA	NA	NA	NA	NA
CaGMS1945	(ATA)7	(ATA)6	GTGGGGgTGAAGTGGAAGTA	CGGTTTAATTCCGTCCCAAT	59.8	198	NA	NA	NA	NA	NA	NA	NA	NA
CaGMS1946	(TTA)6	(TTA)5	TGTCAAAGATTATTCTTGTTGCTTC	TTGGAACATTCTTCTTGGGC	58.9	192	NA	NA	NA	NA	NA	NA	NA	NA
CaGMS1947	(AAT)6	(AAT)5	CCTGGACCTCCAGTTAACCA	CACATTCTACAAATCTTGATAGTGCC	60.0	271	NA	NA	NA	NA	NA	NA	NA	NA
CaGMS1948	(ATT)6	(ATT)5	GTCCCTGCAAGAAATGGAAG	ATCTTGCCTCGTTCTCCTGA	59.7	113	NA	NA	NA	NA	NA	NA	NA	NA
CaGMS1949	(ATA)7	(ATA)6	TTTCTGTCGATCAAGGCTCA	AGCTTTAATGTGGTGGTGCC	59.5	202	NA	NA	NA	NA	NA	NA	NA	NA
CaGMS1950	(AAT)6	(AAT)5	CCCTTTGTAAATCATAGCCTTTT	TGGATGCTTTATTCATTTGGTG	57.9	245	NA	NA	NA	NA	NA	NA	NA	NA
CaGMS1951	(ATC)6	(ATC)5	CTGTGTTTTGCTCCCCAAAT	ATGGTTTTCCATTCCTCCAA	60.0	250	NA	NA	NA	NA	NA	NA	NA	NA
CaGMS1952	(TTC)19	(TTC)6	CGCTGTAGAGAACCTCCCAC	CCGAGCCAAAATCCAAAATA	59.9	173	NA	NA	NA	NA	NA	NA	NA	NA
CaGMS1953	(AAT)9	(AAT)8	TTTAAAACTGCGATACAAATGACTT	AAACCGAATTATAAATGCAAAAA	58.5	280	NA	NA	NA	NA	NA	NA	NA	NA
CaGMS1954	(CTC)8	(CTC)7	CCTcTTCCCCATCAACTTCA	AGGCAGGCAACAAAAGAAAA	60.0	174	NA	NA	NA	NA	NA	NA	NA	NA
CaGMS1955	(TAT)6	(TAT)5	CCCTTTGTCCTACAGTGAACG	TTGTTTTCATTTTACAACGAGTGAA	59.6	166	NA	NA	NA	NA	NA	NA	NA	NA
CaGMS1956	(TAT)6	(TAT)5	TTCGAATGGAGGGTGAAAAG	AACGCAACCATATACGCCTC	60.0	160	NA	NA	NA	NA	NA	NA	NA	NA
CaGMS1957	(AAC)6	(AAC)5	CGCAATATTCATCAAATGGG	TTAGGAACAAGCCATCCAGC	58.8	139	NA	NA	NA	NA	NA	NA	NA	NA
CaGMS1958	(TTA)6	(TTA)5	TGAGAAATGAGAATGGGATGC	CAATAAAAGAAGAAAAATGCATACAAA	60.0	233	NA	NA	NA	NA	NA	NA	NA	NA
CaGMS1959	(TAT)6	(TAT)5	AACAGTGATTGACGTTGATGTTT	TTTTCTTGGCAGATACCCACT	58.6	261	NA	NA	NA	NA	NA	NA	NA	NA
CaGMS1960	(AAT)7	(AAT)6	GGGTCCTTCCGCtAGAAAAA	TTGTAACGCCCCAGATTTTG	60.6	250	NA	NA	NA	NA	NA	NA	NA	NA
CaGMS1961	(AAT)6	(AAT)5	TCAGCGTAACATTGACTATTTCA	TATTTTGCGGGAGTTCAACC	57.6	248	NA	NA	NA	NA	NA	NA	NA	NA
CaGMS1962	(TCT)6	(TCT)5	CATTTCGCCTCCATTTCACT	TGTTCGGAAACCGAAAGAAC	60.1	231	NA	NA	NA	NA	NA	NA	NA	NA
CaGMS1963	(TAT)8	(TAT)7	CAAGTGGAACCCACCAATTT	TCTCTGGCAACCGATCTTCT	59.7	172	NA	NA	NA	NA	NA	NA	NA	NA
CaGMS1964	(ATT)13	(ATT)12	TGATAAAACTATCAATATTCCTCCAA	TGTGGTTTATTGTAATGGAAGTGG	57.4	264	NA	NA	NA	NA	NA	NA	NA	NA
CaGMS1965	(TAT)6	(TAT)5	TTATTTCACCGTCGTGTCCA	CAACGTCCGTTAAAACGGTTA	60.0	278	NA	NA	NA	NA	NA	NA	NA	NA
CaGMS1966	(AAT)6	(AAT)5	TACATTTGACACCCCTGCTG	TGGGAAGACTATGGGAGGAA	59.6	234	NA	NA	NA	NA	NA	NA	NA	NA
CaGMS1967	(TGT)7	(AATA)7	TTGGGGACAAGAGTCCAAAC	CATCATTTCGCACTCCTCAA	59.9	260	NA	NA	NA	NA	NA	NA	NA	NA
CaGMS1968	(CCA)6	(CCA)5	TGAAGGAGGTGATGAAAAGGA	GTGGAACTTCCCACACCAAC	59.7	131	NA	NA	NA	NA	NA	NA	NA	NA
CaGMS1969	(TAT)6	(TAT)5	TGGTACAGAGGCCTATTCCG	TCTCCCTCATCAGTGTCACG	60.1	279	NA	NA	NA	NA	NA	NA	NA	NA
CaGMS1970	(ATT)7	(TA)8	CAAGACTTCTCTTCTGGTCGG	CCCCAGATTATCATTTCACTCC	59.1	241	NA	NA	NA	NA	NA	NA	NA	NA
CaGMS1971	(TTA)7	(GA)9	CTCATTCCCCAAGTGAAACC	TCGTATTTACGTTGCCTCCC	59.4	224	NA	NA	NA	NA	NA	NA	NA	NA
CaGMS1972	(AAT)10	(AAT)9	TGGGCTATTTGATTCGGATT	ACCTCCCTATGCCACTCCTT	59.4	278	NA	NA	NA	NA	NA	NA	NA	NA
CaGMS1973	(CAA)6	(CAA)5	CCATCAGTGCATTCTTGTGG	TCTCTTTGGTTTGCTTCTGTGA	60.1	246	NA	NA	NA	NA	NA	NA	NA	NA
CaGMS1974	(TTA)7	(TTA)6	CATGGTAGAATGAGGAATTAGATCG	TCTCCCATTTTAATAAGATTGATGG	60.2	233	NA	NA	NA	NA	NA	NA	NA	NA
CaGMS1975	(TAT)15	(TAT)14	TTCACATACCGCCACGTTAC	CTTGCTCCTACGTATCCCCa	59.5	280	NA	NA	NA	NA	NA	NA	NA	NA
CaGMS1976	(ATA)6	(ATA)5	CCCCGTTTGAAGAAGGAACT	CAAGGACTAAATTGATGCCCA	60.5	263	NA	NA	NA	NA	NA	NA	NA	NA
CaGMS1977	(ATT)12	(ATT)11	GCTGTCTGCAGAGTGCATTT	GAACGTCATCATACTACGTGGC	59.2	114	NA	NA	NA	NA	NA	NA	NA	NA
CaGMS1978	(ATA)6	(ATA)5	TTGATCTCCAACAAAACTGTCAA	TCATTCTCATGAATAGTTGTGATGTC	59.6	174	NA	NA	NA	NA	NA	NA	NA	NA
CaGMS1979	(ATG)11	(ATG)10	CATTCAGCCCcAAAaCcTAa	TGTTTGGGCTTTTTGCTAAC	59.9	218	NA	NA	NA	NA	NA	NA	NA	NA
CaGMS1980	(ACC)7	(ACC)6	ATGGTCCAACATTTGCCACT	GATTCTCCGCTGGTGGAATA	60.2	217	NA	NA	NA	NA	NA	NA	NA	NA
CaGMS1981	(ATA)7	(ATA)6	CGTAACTTTGATTTCTCCATCG	CTTGGTTCCAACAAAGGGAA	58.8	267	NA	NA	NA	NA	NA	NA	NA	NA
CaGMS1982	(TCC)6	(TCC)5	CGACTTCGAGTTCCGTCTTC	AGAGAAGAGAGAGGGGTCGG	60.0	184	NA	NA	NA	NA	NA	NA	NA	NA
CaGMS1983	(TAT)8	(TAT)7	ATTGTGGAAATCGACCGTGT	TTTTTGTCGATTTGCTTTAATTT	60.2	117	NA	NA	NA	NA	NA	NA	NA	NA
CaGMS1984	(ATT)6	(ATT)5	CAACCAACCTTTTTAGCCACA	GAAGGaAAATGAATaAAAATCAACC	60.0	208	NA	NA	NA	NA	NA	NA	NA	NA

*Markers identity	Microsatellite repeat-motifs in ICC4958	Microsatellite repeat-motifs in PI489777	Forward primer sequences (5'-3')	Reverse primer sequences (5'-3')	Actual annealing temperature (0C)	Size (bp) of alleles amplified	Linkage groups (LGs)	Genetic positions (cM)	Markers used for polymorphism survey	Polymorphism information content (PIC)	Number of alleles amplified	Markers used in Figures	Markers types	GC%
CaGMS1985	(CTT)6	(CTT)5	TCATTTCCCTAACTTGCGTG	CCGGTAAAAACTTCGCTCAA	58.8	201	NA	NA	NA	NA	NA	NA	NA	NA
CaGMS1986	(TTC)9	(TTC)8	TTTtGTTtACTGCTCCACTCTTTG	TCAAAGGGGAGACTTTTGGA	59.9	118	NA	NA	NA	NA	NA	NA	NA	NA
CaGMS1987	(CCG)6	(CCG)5	CATAGGCCTTGCTTCCTCAA	CGAGTAGGTCGGAAGAGTCG	60.3	116	NA	NA	NA	NA	NA	NA	NA	NA
CaGMS1988	(ATT)7	(ATT)6	AAACCAGGGGAGTTCTGCAT	GTGTTGTTCCGCCATTTTCT	60.9	183	NA	NA	NA	NA	NA	NA	NA	NA
CaGMS1989	(CCA)8	(CCA)7	AGCATGCAAAAGCATGTTGA	TGTGCCAAGTGATGtTGGTT	60.4	269	NA	NA	NA	NA	NA	NA	NA	NA
CaGMS1990	(ATA)6	(ATA)5	TGAATGCATTTAATGTCTCTCACTT	TAAAACGCAGGCCTTGTACC	59.2	188	NA	NA	NA	NA	NA	NA	NA	NA
CaGMS1991	(ATT)6	(ATT)5	GGAAAAGTATGTTTCGGAAAATTG	AATGCGCTGTGCTTTATTTTT	60.1	172	NA	NA	NA	NA	NA	NA	NA	NA
CaGMS1992	(ATA)7	(ATA)6	TTTCACACAAACCGTTAAGAAA	GACATAAATTTCGGGGACGA	57.4	268	NA	NA	NA	NA	NA	NA	NA	NA
CaGMS1993	(TTA)13	(TTA)12	CCAGCTGCACTTGTTGTTTT	GCTCATCCATACCCCGTTTA	59.0	278	NA	NA	NA	NA	NA	NA	NA	NA
CaGMS1994	(GAA)7	(GAA)6	CCAAAACGATTTCCCTTCAA	CTTGTGCATCCAATAGGTGTG	59.9	165	NA	NA	NA	NA	NA	NA	NA	NA
CaGMS1995	(ATA)6	(ATA)5	TGACAATGCAATACTAATATGCAAAA	GAGATAAGTTACAATATGTGTGCGCT	59.8	242	NA	NA	NA	NA	NA	NA	NA	NA
CaGMS1996	(AGG)6	(AGG)5	ATGTGAGCCCATGCATGATA	TCCATGGTCGACATACTTGC	59.9	170	NA	NA	NA	NA	NA	NA	NA	NA
CaGMS1997	(AT)7	(AT)6	TTCATGGGACCATTGAGTCAT	GGCGTCAACGAGAAACTCAT	60.2	264	NA	NA	NA	NA	NA	NA	NA	NA
CaGMS1998	(AT)8	(AT)7	GCAATTTTACTCTCCTTCGCAT	CCATTGATTAAAAGATAGTGTGATGG	59.8	210	NA	NA	NA	NA	NA	NA	NA	NA
CaGMS1999	(GA)7	(GA)6	TGCATTCCATCACATTCTAACA	GAGTTTATTTTCATATCAGATCACGG	59.0	210	NA	NA	NA	NA	NA	NA	NA	NA
CaGMS2000	(TC)9	(TC)8	GTCGTCAGTGATTCAGCCAA	AAcAAGAATTGTTAgGCgGG	59.8	146	NA	NA	NA	NA	NA	NA	NA	NA
CaGMS2001	(AT)9	(AT)8	TGTAAAACAATTCACATTGTCTGTG	CTGAGGATATGTTCCACCATGA	59.0	143	NA	NA	NA	NA	NA	NA	NA	NA
CaGMS2002	(GA)15	(GA)14	CACGTGGGTGAGTTATGGTG	CCTTCATCTTCTTcTcCCCC	59.9	246	NA	NA	NA	NA	NA	NA	NA	NA
CaGMS2003	(AG)9	(AG)8	TTGTTTGTTGTTTTGTGAGAGTGA	AAGTCCAGTCACCCTTTCCC	59.7	162	NA	NA	NA	NA	NA	NA	NA	NA
CaGMS2004	(AT)8	(AT)7	GAGAATTTAAGAGGGGCCAAA	CATCCATTCAACATCTCATTTTC	59.6	251	NA	NA	NA	NA	NA	NA	NA	NA
CaGMS2005	(TG)8	(TG)7	CAGCTATTGTTGCTCTAGGATTCA	TTcCCCCTCAATGCTATGTC	59.9	279	NA	NA	NA	NA	NA	NA	NA	NA
CaGMS2006	(AT)10	(AT)9	GCAGGAATGACTGCTTCGTT	TTTTGCCTGTTCTTGTTGTcC	60.4	276	NA	NA	NA	NA	NA	NA	NA	NA
CaGMS2007	(CA)8	(CA)7	TTGGAGCAGCTTCATTTGTG	CTCAGAACCTTTCCCATCCA	60.0	245	NA	NA	NA	NA	NA	NA	NA	NA
CaGMS2008	(AG)8	(AG)7	CCAAGGAAACTGGAACCTGA	GTGGTGGCTGAAGGAAGAAG	60.1	144	NA	NA	NA	NA	NA	NA	NA	NA
CaGMS2009	(TG)7	(TG)6	GTCGATCAAAAGTGGTGCCT	TCACCGAATGCTTTGAATTG	60.1	155	NA	NA	NA	NA	NA	NA	NA	NA
CaGMS2010	(AT)8	(AT)7	GCATGAGCATGGCATATGTT	GCCAAGACAGAACCCTACCA	59.5	253	NA	NA	NA	NA	NA	NA	NA	NA
CaGMS2011	(AT)11	(CT)6	CTTCGATGTGTGCTTTGACG	tGTGAATATTGCCTAAAGACTCG	60.4	235	NA	NA	NA	NA	NA	NA	NA	NA
CaGMS2012	(TC)16	(TA)6	TGGTTCCGTATAAAATGGACCT	GCAGATTGAAGAATCCGGAAC	59.6	238	NA	NA	NA	NA	NA	NA	NA	NA
CaGMS2013	(TA)7	(TA)6	CCGTGTGAAGGAAAGCAAAT	AAGCACATGTGTCAGCTTCCT	60.1	107	NA	NA	NA	NA	NA	NA	NA	NA
CaGMS2014	(CT)7	(CT)6	AAAATGCCCACAAgAGCAAG	TGCAACTCGACaAATCAATCA	60.2	218	NA	NA	NA	NA	NA	NA	NA	NA
CaGMS2015	(TA)9	(TA)8	GATTCATTTCTTCTTTCACCCA	TCGTTGCAGATCAACGTTTC	58.2	266	NA	NA	NA	NA	NA	NA	NA	NA
CaGMS2016	(CT)7	(CT)6	TGAGCTAATGCCTCATCTGG	TGTGGAAAATGACTCACCCTT	59.0	210	NA	NA	NA	NA	NA	NA	NA	NA
CaGMS2017	(AG)7	(AG)6	GGCGGAGATGGTGGTAGTTA	ACGGAGACGCACACATACAA	60.0	191	NA	NA	NA	NA	NA	NA	NA	NA
CaGMS2018	(TC)11	(TC)10	TATGGAGATGGAGGTCGAGG	ATCAAAAGCAGGGTCTCGAA	60.0	255	NA	NA	NA	NA	NA	NA	NA	NA
CaGMS2019	(TC)9	(TC)8	GCTGGTGGGTGGACTTAGAA	TCCTTCCTCAAACAAAGCAGA	60.1	246	NA	NA	NA	NA	NA	NA	NA	NA
CaGMS2020	(AT)7	(AT)6	ATTTTCCCAAATTGAACCCC	CACGATTGTTTAAAGGTTGGC	59.9	154	NA	NA	NA	NA	NA	NA	NA	NA
CaGMS2021	(AT)8	(AT)7	TTGATAATGACCCTCCACACA	CACTCGATAGTGGCTGCTGA	58.8	219	NA	NA	NA	NA	NA	NA	NA	NA
CaGMS2022	(AT)7	(AT)6	CCTGTTGCGATTTGAATGTG	GCGGGTTAAGGTTTCTGTGA	60.1	160	NA	NA	NA	NA	NA	NA	NA	NA
CaGMS2023	(AT)7	(AT)6	GCAACTTATTAACCTTCCATTGAATA	GGTGGCGGATCTTATTAACC	59.0	232	NA	NA	NA	NA	NA	NA	NA	NA
CaGMS2024	(AT)7	(AT)6	TCATTTCCCTCCCACCATTA	TGCTGGTGCATATCCCATTA	60.1	248	NA	NA	NA	NA	NA	NA	NA	NA
CaGMS2025	(AT)7	(AT)6	ACGGATTTTCTTGACACCCA	TTCCATGGGATGATAAACTCCT	60.4	148	NA	NA	NA	NA	NA	NA	NA	NA
CaGMS2026	(AT)7	(AT)6	CAATTCGGAGTGCGTTTTCT	AAGaCAAAATGGGCCGACTA	60.2	278	NA	NA	NA	NA	NA	NA	NA	NA
CaGMS2027	(TA)7	(TA)6	CACATATTCCTTTCATCAATGGTC	AAATCCAAAAGCATTGGCAG	59.6	129	NA	NA	NA	NA	NA	NA	NA	NA
CaGMS2028	(TA)9	(TA)8	TAGGAATTCGCATCGTGAGG	CTTTGGAGAATTCAAACATAAGG	61.1	176	NA	NA	NA	NA	NA	NA	NA	NA
CaGMS2029	(CT)8	(CT)7	CTACGCCACGTCAGCAGATA	AGAGGAACGTGTTTGACGCT	60.0	132	NA	NA	NA	NA	NA	NA	NA	NA
CaGMS2030	(AT)9	(AT)8	GGGGACCAATCTTACTCGGT	GGGAAAAACGaTTTTAGACGAA	60.2	278	NA	NA	NA	NA	NA	NA	NA	NA
CaGMS2031	(TA)7	(TA)6	AAATTGCAGGATTGCAAAGG	GCTTTGCTATTTGAGCCCAC	60.1	239	NA	NA	NA	NA	NA	NA	NA	NA
CaGMS2032	(TC)10	(TC)9	AGCGTAAGGGGAGGAGAGAG	TGATGGAAGGAGGAAAGGaA	60.0	182	NA	NA	NA	NA	NA	NA	NA	NA
CaGMS2033	(AT)8	(AT)7	CAAGTCACCTAGACCCTTTGC	CTACGGCGGAATGCATAAAt	58.9	264	NA	NA	NA	NA	NA	NA	NA	NA
CaGMS528	(TA)10	(TA)7	TTGATATCCTTTGGCTGAATG	CTAACCAGGATCCGGAGGA	58.0	250	NA	NA	NA	NA	NA	NA	NA	NA
CaGMS529	(AT)11	(AT)9	TCAGCAGGACTAAAACTGTTTGTT	GGGCTTGCATAAGTTGCATT	59.0	174	NA	NA	NA	NA	NA	NA	NA	NA
CaGMS531	(ATA)7	(ATA)5	CCAATGAATCTCATCAAGTGAA	GGTCATTACACAAACATTTAATCCA	58.0	243	NA	NA	NA	NA	NA	NA	NA	NA
CaGMS532	(AT)9	(AT)6	TGAAGATCGGTCAGAGGAAAA	TGATCAAAAGGTCAATTGCG	60.0	210	NA	NA	NA	NA	NA	NA	NA	NA

*Markers identity	Microsatellite repeat-motifs in ICC4958	Microsatellite repeat-motifs in PI489777	Forward primer sequences (5'-3')	Reverse primer sequences (5'-3')	Actual annealing temperature (0C)	Size (bp) of alleles amplified	Linkage groups (LGs)	Genetic positions (cM)	Markers used for polymorphism survey	Polymorphism information content (PIC)	Number of alleles amplified	Markers used in Figures	Markers types	GC%
CaGMS537	(TA)9	(TA)6	CCTCAATCCCTCTTTCTGGA	AACGCACAATAAACACAGAGAAA	59.0	275	NA	NA	NA	NA	NA	NA	NA	NA
CaGMS538	(AT)9	(AT)6	CCAACGTTGTCACCACTACCT	CCATTTTTGTTTATATTTCATGCTG	60.0	181	NA	NA	NA	NA	NA	NA	NA	NA
CaGMS541	(AGA)7	(AGA)5	TTTCACCAATTGAGGAAGAAGAA	TGGGCTAATTTGTCCTATTTTGA	60.0	269	NA	NA	NA	NA	NA	NA	NA	NA
CaGMS544	(TA)12	(TA)9	ATCGGATAACCGTGTGCATT	CAGTTGATTGCCTCTTATTTTATGG	60.0	238	NA	NA	NA	NA	NA	NA	NA	NA
CaGMS546	(TA)9	(TA)6	TCGATCCATCGATTCACTTG	GCTGCTTTGTGCAGTGAAAG	60.0	200	NA	NA	NA	NA	NA	NA	NA	NA
CaGMS548	(AG)11	(AG)8	GCATATCGAAAGGTGGTGGT	CCCTCCTTCCTTCACAATCA	60.0	217	NA	NA	NA	NA	NA	NA	NA	NA
CaGMS550	(TTA)8	(TTA)5	TGAAGGTTATAGATGATCGCGT	GCGACATATTGGGATGAGCTA	59.0	268	NA	NA	NA	NA	NA	NA	NA	NA
CaGMS551	(AAT)8	(AAT)5	CCACTGCTGTCCTACACTGC	TTGCTTTGAAGAAGGGGAAA	59.0	155	NA	NA	NA	NA	NA	NA	NA	NA
CaGMS552	(AT)10	(AT)7	TTGAATAAAGAAGTTTTATACCGGC	CAACGACAATATAAAAGAAACACAA	59.0	252	NA	NA	NA	NA	NA	NA	NA	NA
CaGMS553	(TTA)7	(TTA)5	CAGGTAAAAGGTTTGAAAAAGACA	ATGAAGTTCCCCATGAGTGC	59.0	148	NA	NA	NA	NA	NA	NA	NA	NA
CaGMS556	(AGA)8	(AGA)6	AAAGCGAAGAGGGAAAGGAG	ATTTGCAATGTTCTGTGCCA	60.0	265	NA	NA	NA	NA	NA	NA	NA	NA
CaTMS1501	(TA)7	(TA)6	CGGAGGAGAAAAACTGACTGA	AGCTGCATCATGGTTCACAA	59.5	227	NA	NA	NA	NA	NA	NA	NA	NA

*CaGMS: *Cicer arietinum* Genomic Microsatellite Markers

*CaTMS: *Cicer arietinum* Transcript-derived Microsatellite Markers

873 experimentally validated markers are marked with red colour
